# Supplementary material for: Isolation and characterization of bis(silylene)-stabilized antimony(I) and bismuth(I) cations
Source: Nat Commun. 2023 May 23;14:2968. doi: 10.1038/s41467-023-38606-2 (PMC10206093; doi:10.1038/s41467-023-38606-2)
Supplement: Supplementary file 1 — Supplementary Information [file 41467_2023_38606_MOESM1_ESM.pdf]

Supplementary Information for

Isolation and Characterization of Bis(silylene)-Stabilized Antimony(I) and Bismuth(I) Cations

Xuyang Wang,[1] Binglin Lei,[1] Zhaoyin Zhang,[2] Ming Chen,[1] Hua Rong,[1] Haibin Song,[1] Lili Zhao*[2] and Zhenbo Mo*[1]

[1]State Key Laboratory and Institute of Elemento-Organic Chemistry, College of Chemistry, Nankai University, Tianjin 300071, China

[2] Institute of Advanced Synthesis, School of Chemistry and Molecular Engineering, State Key Laboratory of Materials-Oriented Chemical Engineering, Nanjing Tech University, Nanjing 211816, China

E-mail: ias_llzhao@njtech.edu.cn; zhenbo.mo@nankai.edu.cn

Table of Contents

1.Supplementary Methods 2

Preparation of Compound 1: 2

Preparation of Compound 2: 5

Preparation of Compound 3: 10

Preparation of Compound 4: 14

Preparation of Compound 5: 18

Preparation of Compound 6: 22

Preparation of Compound 7: 27

Preparation of Compound 8: 31

Preparation of Compound 9: 36

2. Supplementary Discussion..41

X-Ray Crystallography Data: 41

Computational Studies: 54

3. Supplementary References..71

1. Supplementary Methods

Preparation of Compound 1: *n*-BuLi (5 mL of a 1.6 M solution in hexane, 8.0 mmol) was added dropwise to a solution of 1,8,10,9-triaminoborane (556 mg, 4.0 mmol) in THF (20 mL) at -30 °C. The mixture was warmed to room temperature and stirred for a further 16 h. The resulting white solution was precooled to -30 °C, and then a solution of [PhC(N^{t}Bu)$_2$]SiCl (2.36 g, 8.0 mmol) in THF (30 mL) was added slowly. The color of the mixture changed from colorless to dark red gradually. After warming to room temperature and stirring for 4 h, the solvent was removed in vacuum and the residue was extracted with hexane (30 mL). The extract was concentrated to 5 mL and crystallized overnight at -30 °C to afford compound **1** as a red solid (1.84 g, 2.8 mmol, 70%). Single crystals suitable for X-ray diffraction studies were obtained by recrystallization from a hexane solution at room temperature.

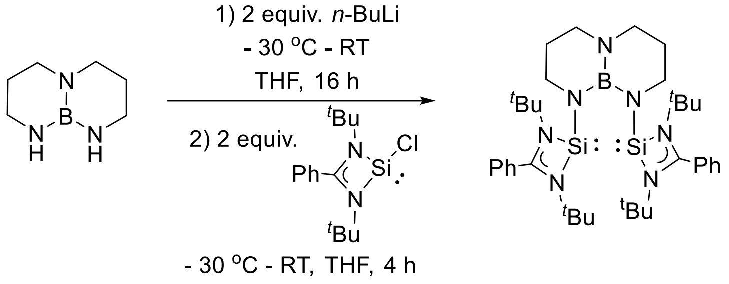

Supplementary Fig. 1. Synthesis of compound 1.

^{1}H NMR (400 MHz, C_6D_6, 298 K): δ 7.15-7.13 (m, 2H, Ar-*H*), 7.04-7.02 (m, 2H, Ar-*H*), 6.97-6.95 (m, 4H, Ar-*H*), 6.90-6.86 (m, 2H, Ar-*H*), 3.25 (m, 4H, NC*H*$_2$), 3.18 (t, 4H, NC*H*$_2$, J = 6.2 Hz), 2.07 (m, 4H, CH$_2$-C*H*$_2$-CH$_2$), 1.39 (s, 36H, C(C*H*$_3$)$_3$).

^{13}C NMR (101 MHz, C_6D_6, 298 K): δ 158.1 (s, N*C*N), 135.9 (s, Ar*C*), 130.7 (s, Ar*C*), 128.9 (s, Ar*C*), 128.8 (s, Ar*C*), 127.6 (s, Ar*C*), 127.5 (s, Ar*C*), 53.2 (s, *C*(CH$_3$)$_3$), 50.0

(s, N*C*H_2), 40.2 (s, N*C*H_2), 32.7 (s, C(*C*H_3)$_3$), 30.0 (s, CH_2-*C*H_2-CH_2).

^{11}B NMR (128 MHz, C_6D_6, 298 K): δ 27.2 ppm (br).

^{29}Si NMR (79 MHz, C_6D_6, 298 K): δ -22.3 ppm (s).

Anal. Calcd for $C_{36}H_{58}BN_7Si_2$: C, 65.93; H, 8.91; N, 14.95. Found: C, 65.65; H, 8.86; N, 14.6

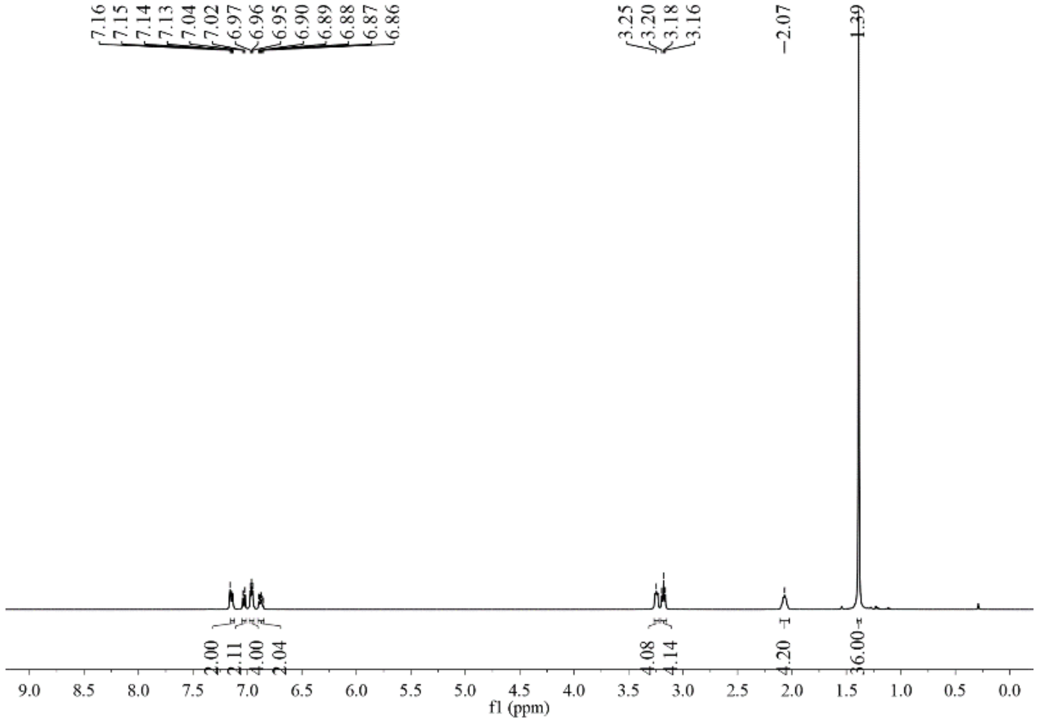

Supplementary Fig. 2. ^{1}H NMR spectrum of **1** in C_6D_6 at 298 K.

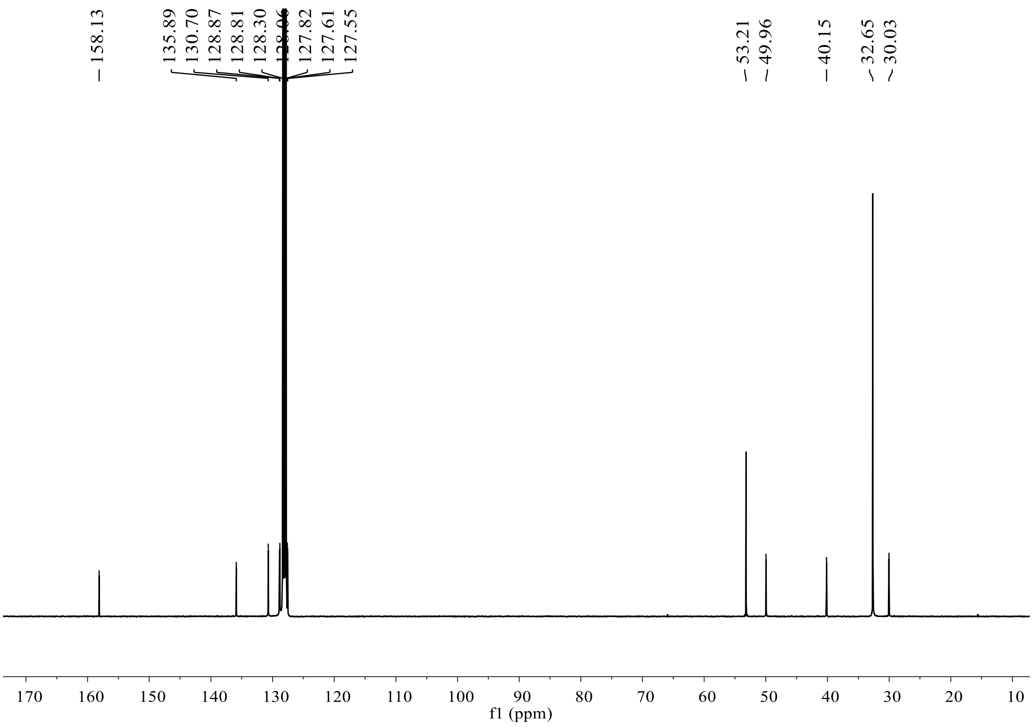

Supplementary Fig. 3. ^{13}C NMR spectrum of **1** in C_6D_6 at 298 K.

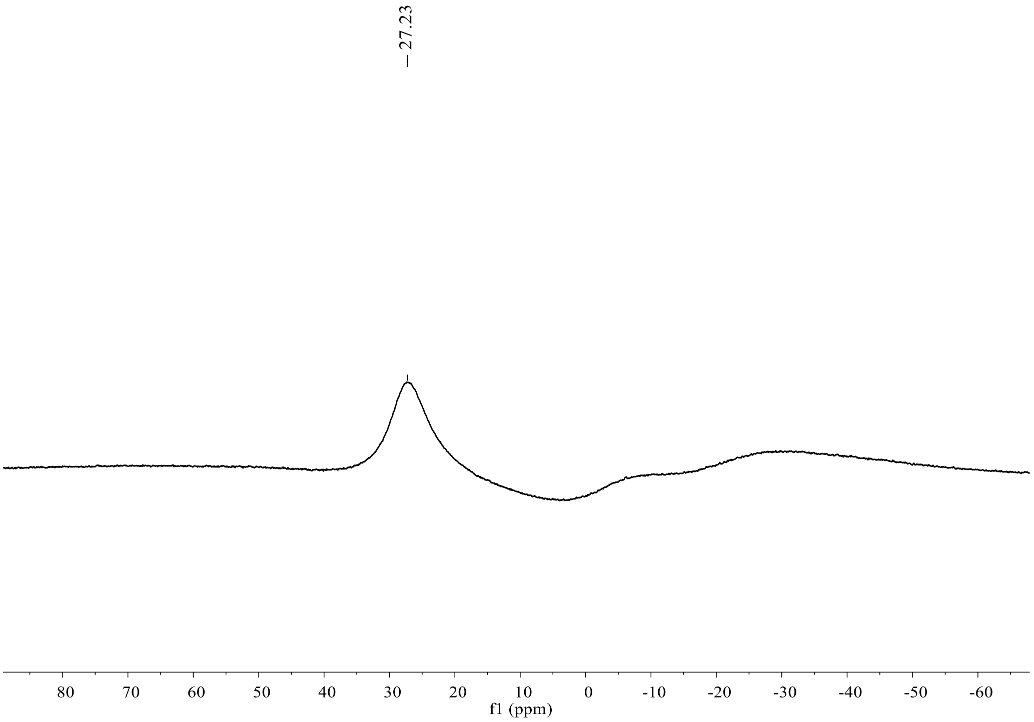

Supplementary Fig. 4. ^{11}B NMR spectrum of **1** in C_6D_6 at 298 K.

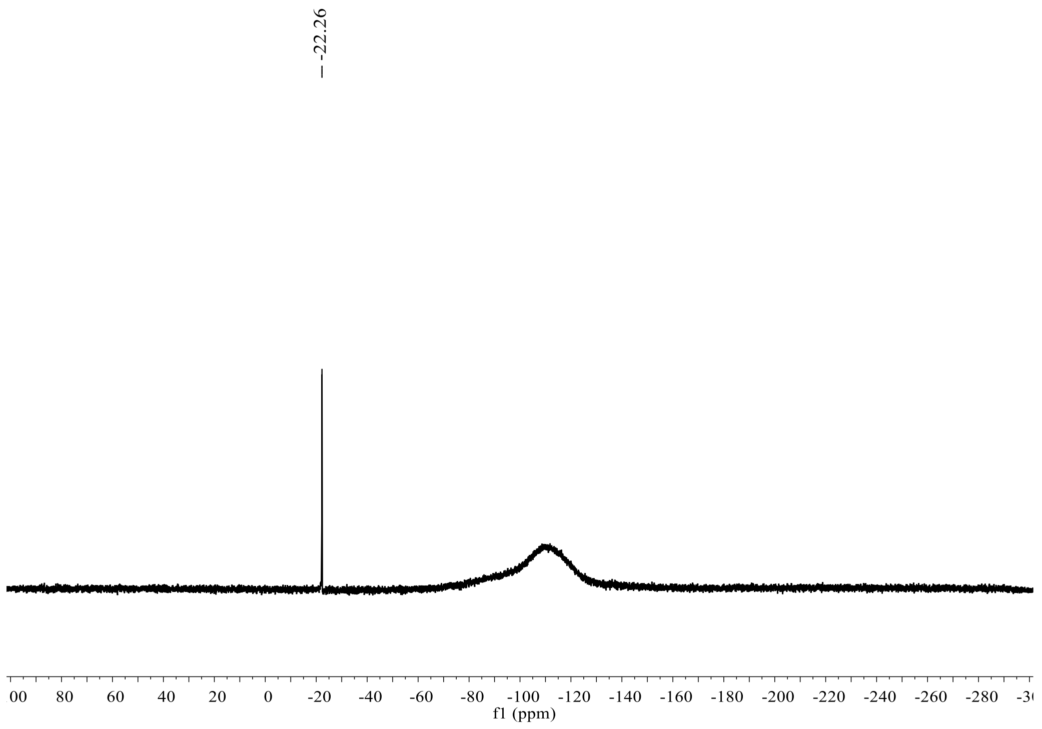

Supplementary Fig. 5. ^{29}Si NMR spectrum of **1** in C_6D_6 at 298 K.

Preparation of Compound 2: THF (15 mL) was cooled to -30 °C and added to a mixture of compound **1** (200 mg, 0.30 mmol), (IPr)$SbBr_3$ (225 mg, 0.30 mmol), KC_8 (81 mg, 0.60 mmol) and Na[BAr^F_4] (266 mg, 0.30 mmol). Then the mixture was warmed to room temperature and stirred for 3 h and filtered. The solvent was removed in vacuum, and the residue was washed with hexane (8 mL), diethyl ether (8 mL) and toluene (8 mL) to yield compound **2** as a yellow powder (265 mg, 0.16 mmol, 53%). Single crystals suitable for X-ray diffraction studies were obtained by slow diffusion of hexane into the saturated THF solutions of **2** at room temperature.

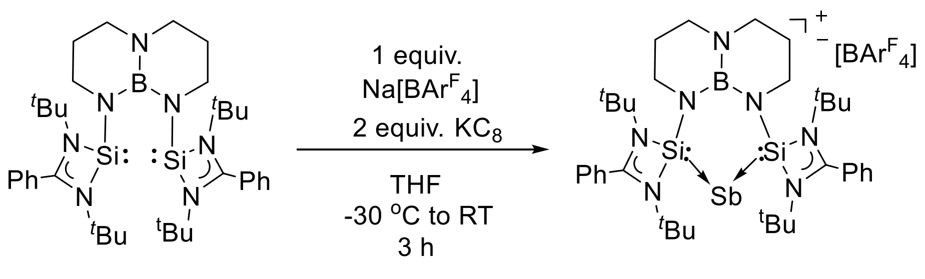

Supplementary Fig. 6. Synthesis of compound **2**.

^{1}H NMR (400 MHz, THF-d_8, 298 K): δ 7.79 (s, 8H, Barf-Ar-*H*), 7.65-7.55 (m, 14H, Ar-*H*), 3.28 (br, 4H, NCH_2), 3.02 (t, 4H, NCH_2, J = 6.9 Hz), 1.95 (br, 4H, CH_2-CH_2-CH_2), 1.30 (s, 36H, C(CH_3)$_3$).

^{13}C NMR (101 MHz, THF-d_8, 298 K): δ 176.8 (s, N*C*N), 162.8 (q, $J_{C\text{-}B}$= 51 Hz, Barf-Ar-*C*), 135.6 (s, Ar*C*), 132.4 (s, Ar*C*), 130.3 (s, Ar*C*), 130.2 (m, Barf-Ar-*C*), 129.9 (m, Barf-Ar-*C*), 129.5 (s, Ar*C*), 129.4 (s, Ar*C*), 128.4 (s, Ar*C*), 125.5 (q, $J_{C\text{-}F}$ = 274 Hz, Barf-*C*F_3), 118.2 (m, Barf-Ar-*C*), 56.7 (s, N*C*(CH_3)$_3$), 50.0 (s, N*C*H_2), 42.9 (s, N*C*H_2), 31.2 (s, C(*C*H_3)$_3$), 28.2 (s, CH_2-*C*H_2-CH_2).

^{11}B NMR (128 MHz, THF-d_8, 298 K): δ 26.6 ppm (br), -6.5 ppm (s, Barf-*B*).

^{19}F NMR (377 MHz, THF-d_8, 298 K): δ -63.4 ppm (s).

^{29}Si NMR (79 MHz, THF-d_8, 298 K): δ -8.7 ppm (s).

Anal. Calcd for $C_{68}H_{70}B_2SbF_{24}N_7Si_2$: C, 49.78; H, 4.30; N, 5.98. Found: C, 49.42; H, 4.51; N, 5.58.

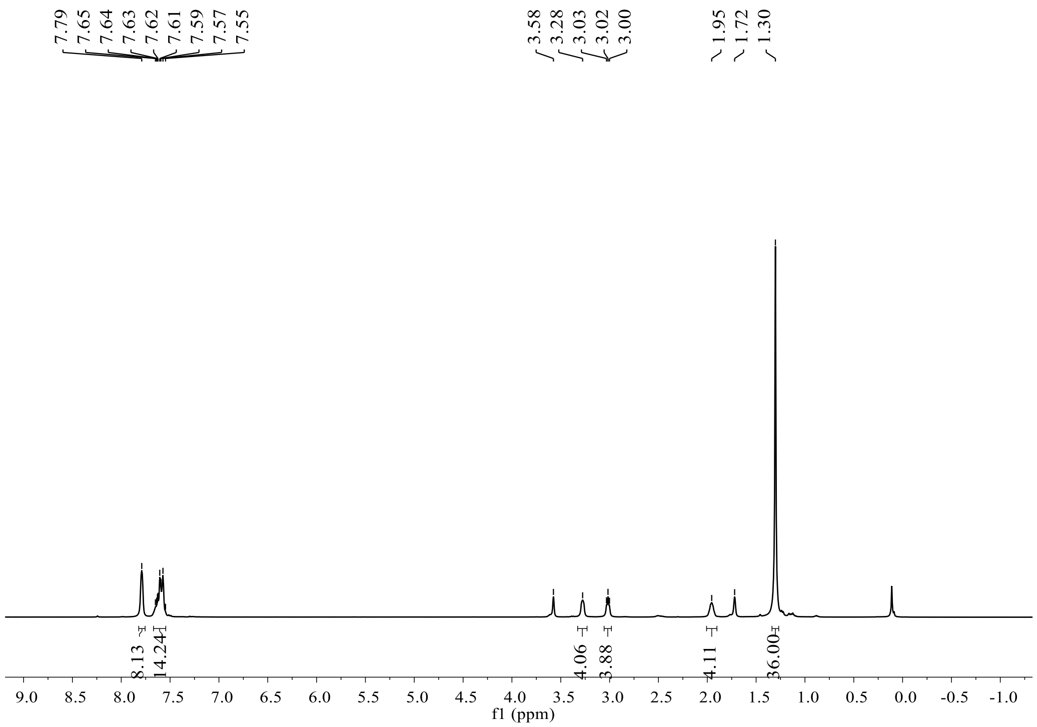

Supplementary Fig. 7. ^{1}H NMR spectrum of **2** in THF-d_8 at 298 K.

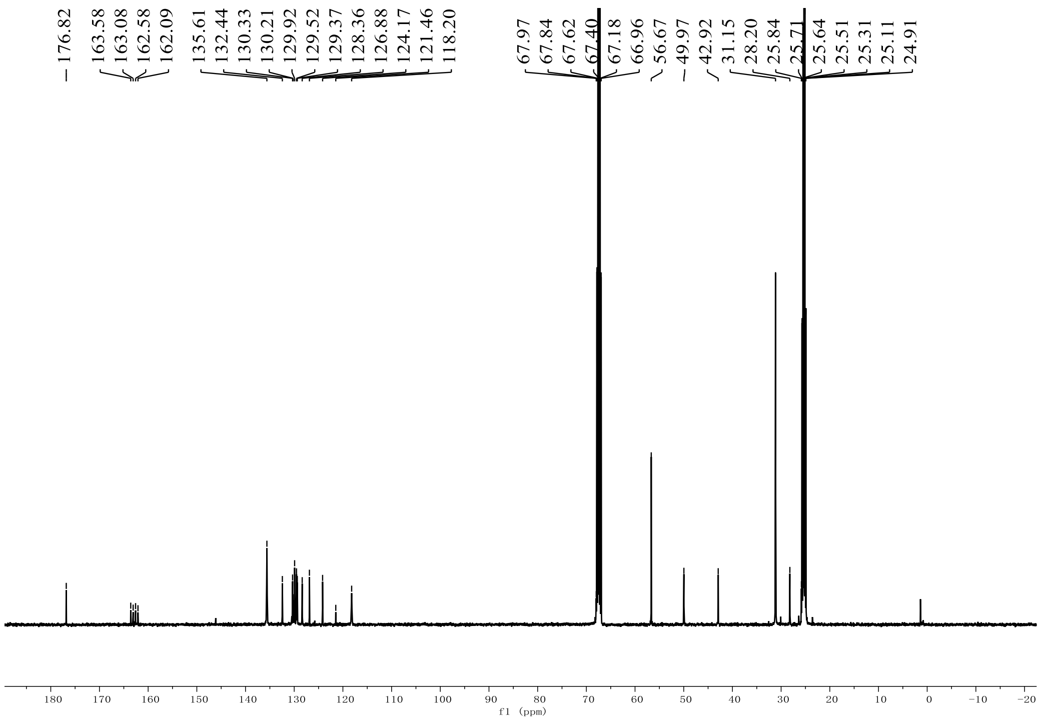

Supplementary Fig. 8. ^{13}C NMR spectrum of **2** in THF-d_8 at 298 K.

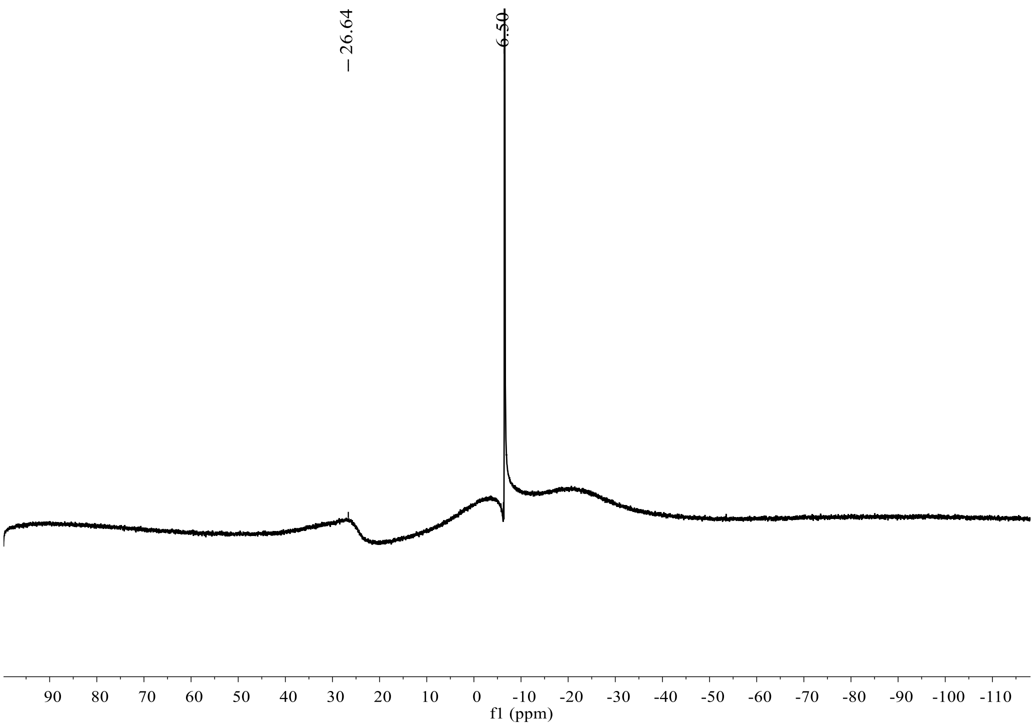

Supplementary Fig. 9. ^{11}B NMR spectrum of **2** in THF-d_8 at 298 K.

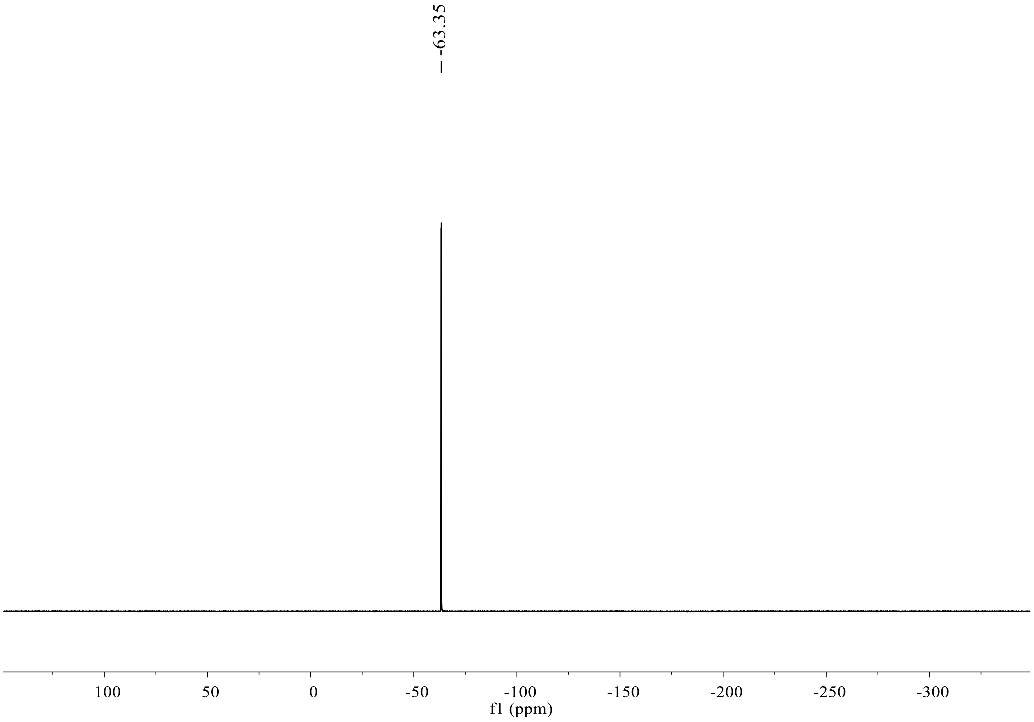

Supplementary Fig. 10. ^{9}F NMR spectrum of **2** in THF-d_8 at 298 K.

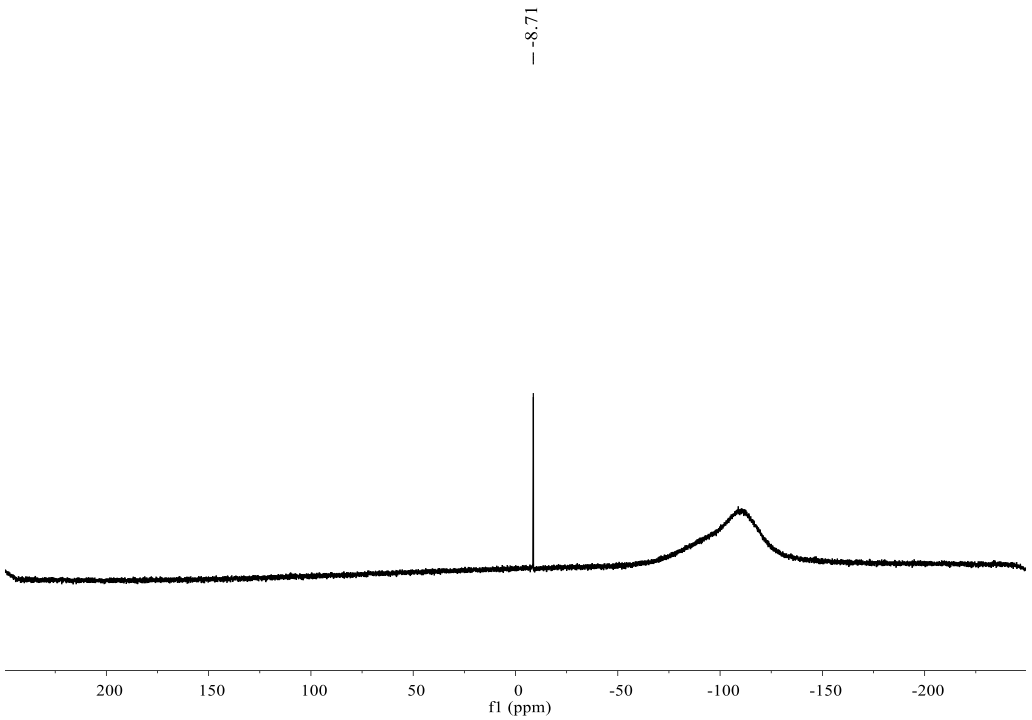

Supplementary Fig. 11. ^{29}Si NMR spectrum of **2** in THF-d_8 at 298 K.

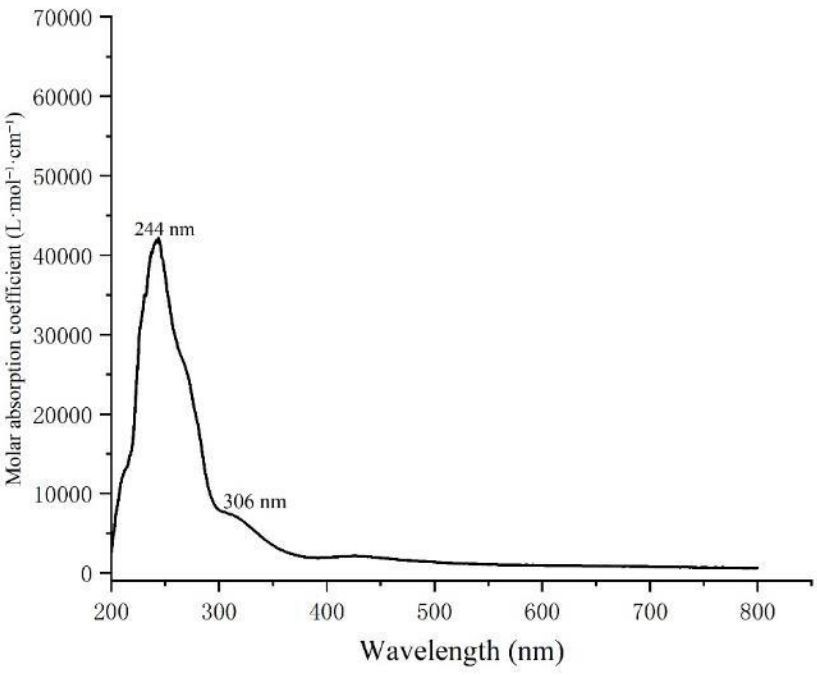

Supplementary Fig. 12. UV/Vis spectrum of compound **2** (6.094 × 10^{-5} mol/L in THF at 298 K).

Preparation of Compound 3: THF (15 mL) was cooled to -30 °C and added to a mixture of compound **1** (200 mg, 0.30 mmol), (IPr)$BiBr_3$ (251 mg, 0.30 mmol), KC_8 (81 mg, 0.60 mmol) and Na[BAr^F_4] (266 mg, 0.30 mmol). Then the mixture was warmed to room temperature and stirred for 3 h and filtered. The solvent was removed in vacuum, and the residue was washed with hexane (8 mL), diethyl ether (8 mL) and toluene (8 mL) to yield compound **3** as an orange powder (231 mg, 0.13 mmol, 44%). Single crystals suitable for X-ray diffraction studies were obtained by liquid phase diffusion of a solution of THF with hexane at room temperature.

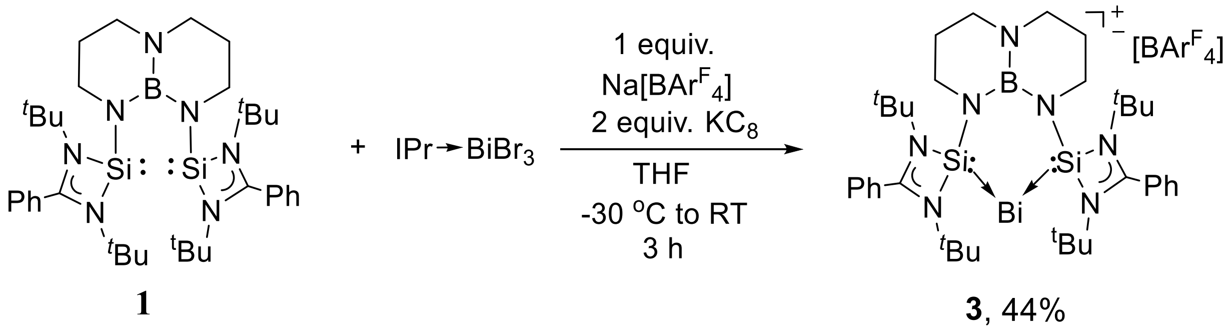

Supplementary Fig. 13. Synthesis of compound **3**.

^{1}H NMR (400 MHz, $CDCl_3$, 298 K): δ 7.71 (s, 8H, Barf-Ar-*H*), 7.59-7.47 (m, 9H, Ar-*H*), 7.39-7.32 (m, 5H, Ar-*H*), 3.18 (br, 4H, NCH_2), 2.94 (br, 4H, NCH_2), 1.91 (br, 4H, CH_2-CH_2-CH_2), 1.26 (s, 36H, C(CH_3)$_3$).

^{13}C NMR (101 MHz, $CDCl_3$, 298 K): δ 175.1 (s, N*C*N), 161.9 (q, $J_{C\text{-}B}$= 51 Hz, Barf-Ar-*C*), 135.0 (s, Ar*C*), 131.7 (s, Ar*C*), 130.4 (s, Ar*C*), 129.4 (s, Ar*C*), 129.2 (m, Barf-Ar-*C*), 128.8 (m, Barf-Ar-*C*), 128.7 (s, Ar*C*), 127.5 (s, Ar*C*), 124.7 (q, $J_{C\text{-}F}$ = 273 Hz, Barf-*C*F_3), 117.6 (m, Barf-Ar-*C*), 55.8 (s, N*C*(CH_3)$_3$), 49.1 (s, N*C*H_2), 42.6 (s, N*C*H_2), 31.6 (s, C(*C*H_3)$_3$), 27.5 (s, CH_2-*C*H_2-CH_2).

^{11}B NMR (128 MHz, THF-d_8, 298 K): δ 27.0 ppm (br), -6.5 ppm (s, Barf-*B*).

^{19}F NMR (377 MHz, THF-d_8, 298 K): δ -65.2 ppm (s).

^{29}Si NMR (79 MHz, THF-d_8, 298 K): δ -28.6 ppm (s).

Anal. Calcd for $C_{68}H_{70}B_2BiF_{24}N_7Si_2$: C, 47.26; H, 4.08; N, 5.67. Found: C, 47.55; H, 4.21; N, 5.10.

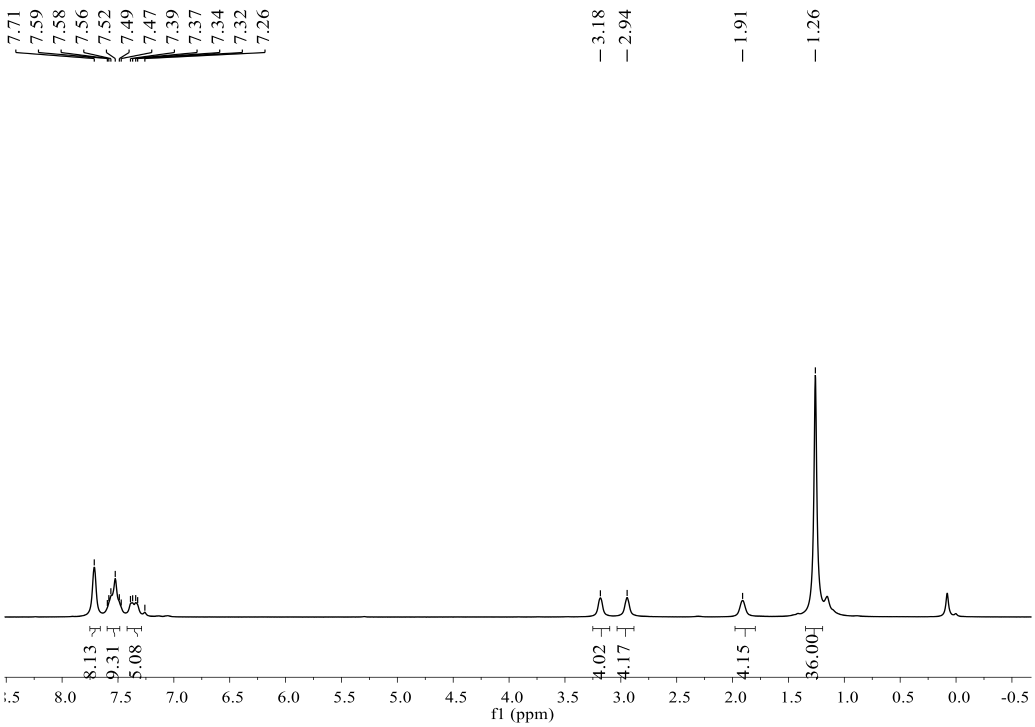

Supplementary Fig. 14. ^{1}H NMR spectrum of **3** in $CDCl_3$ at 298 K.

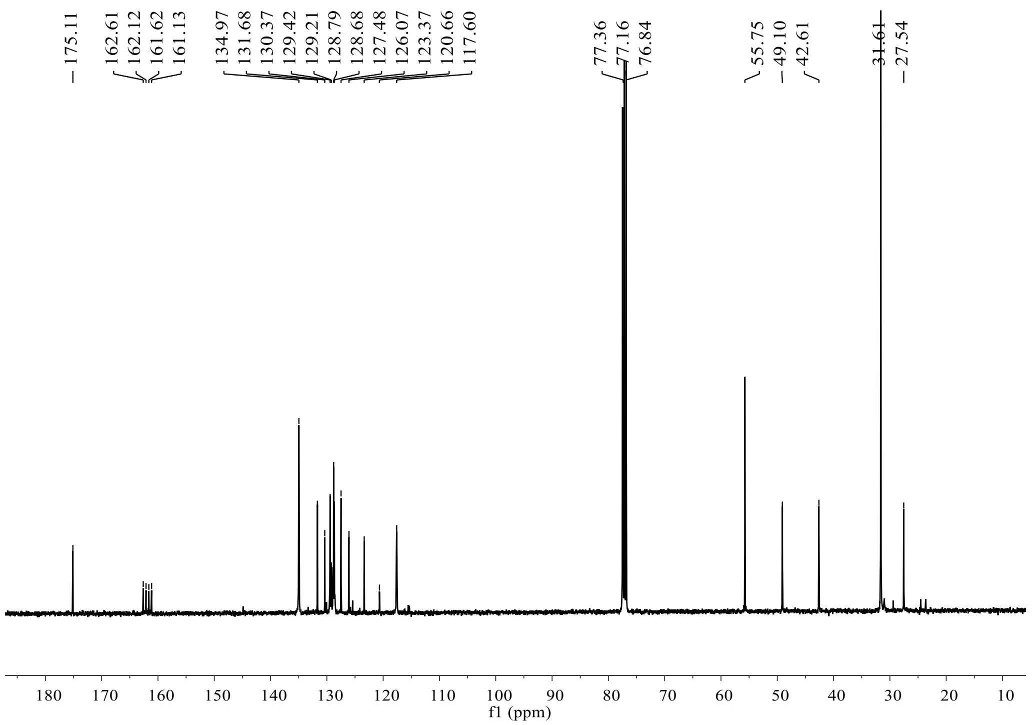

Supplementary Fig. 15. ^{13}C NMR spectrum of **3** in $CDCl_3$ at 298 K

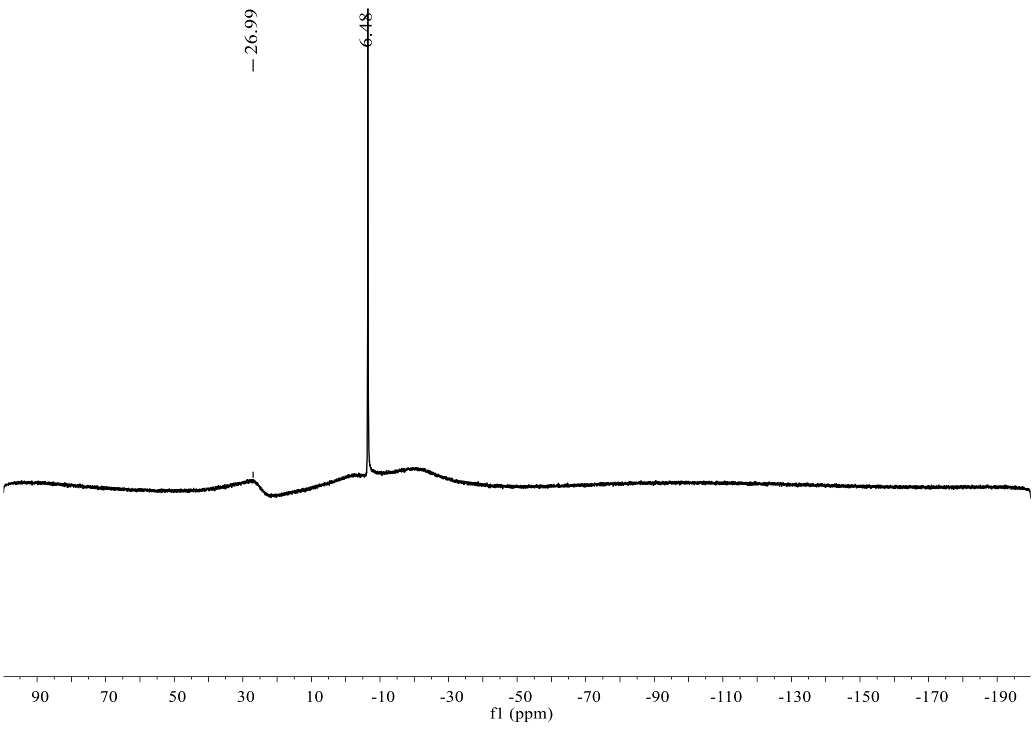

Supplementary Fig. 16. ^{11}B NMR spectrum of **3** in THF-d_8 at 298 K.

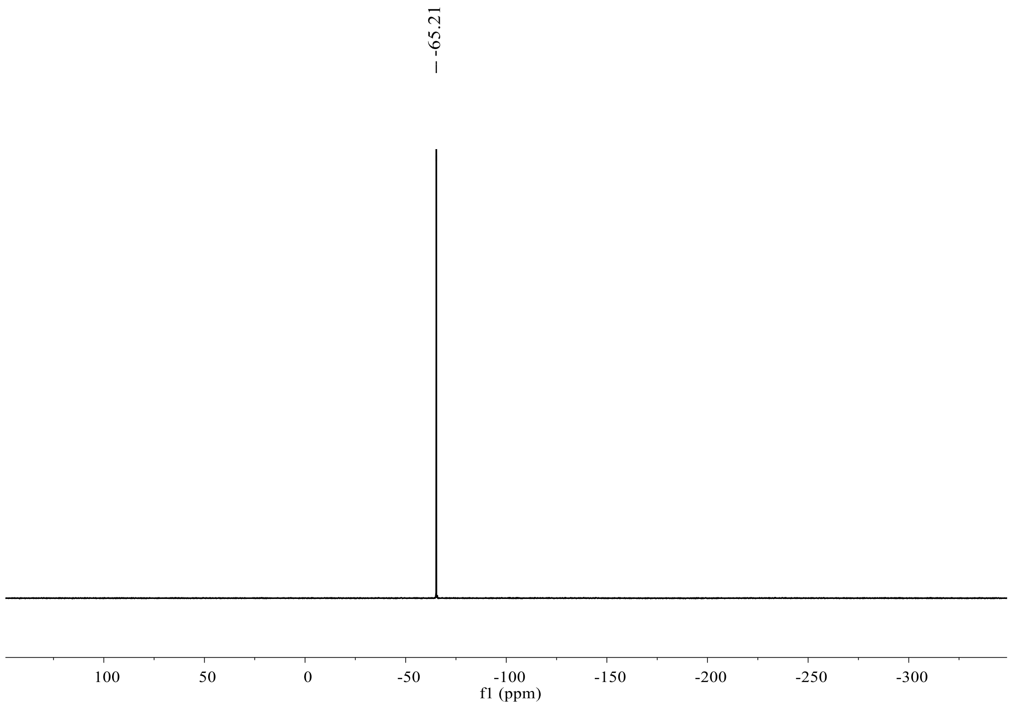

Supplementary Fig. 17. ^{19}F NMR spectrum of **3** in THF-d_8 at 298 K.

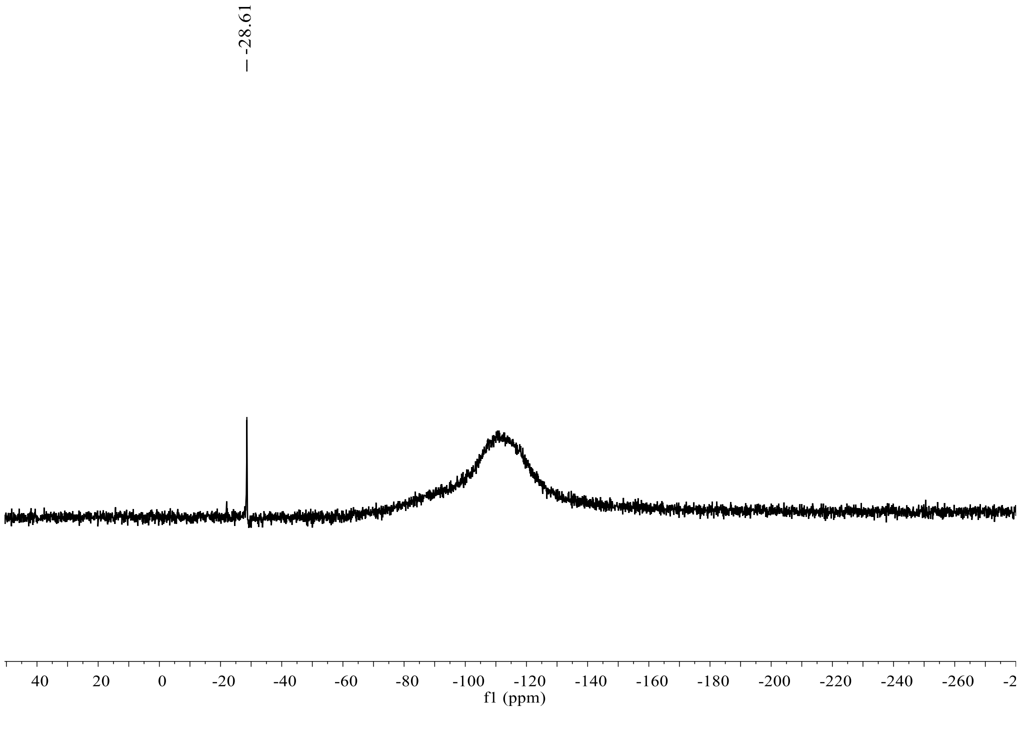

Supplementary Fig. 18. ^{29}Si NMR spectrum of **3** in THF-d_8 at 298 K.

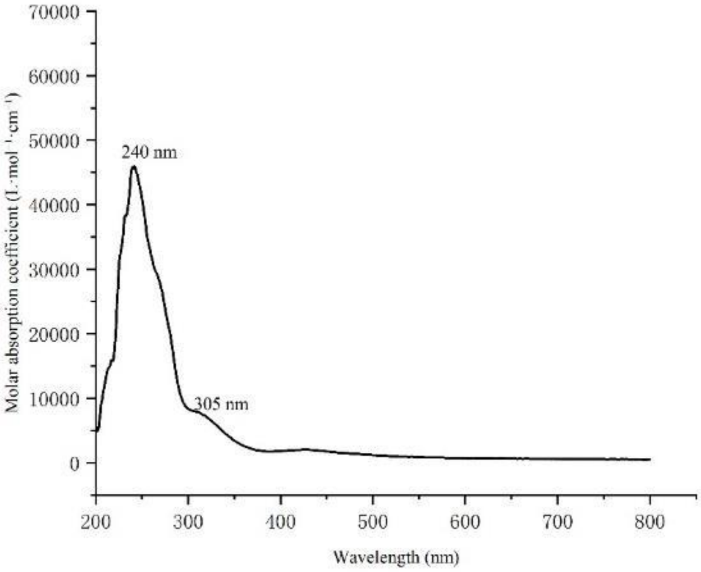

Supplementary Fig. 19. UV/Vis spectrum of compound **3** (5.786×10^{-5} mol/L in THF at 298 K).

Preparation of Compound 4: MeOTf (10.3 μL, 0.098 mmol) was added to **2** (160 mg, 0.098 mmol) in fluorobenzene (10 mL) at -30 ^{o}C. Then Na[BAr^{F}$_{4}$] (87 mg, 0.098 mmol) was added to this mixture immediately. The solution was stirred for 30 minutes and the color changed from yellow to white. The reaction solution was diluted with 5 mL of hexane with rigorous stirring to give a white precipitate. After decanting the supernatant, the precipitate was washed with diethyl ether (6 mL) and dried under vacuum to give **4** as a colorless, moisture sensitive solid (199 mg, 0.076 mmol, 78% yield).

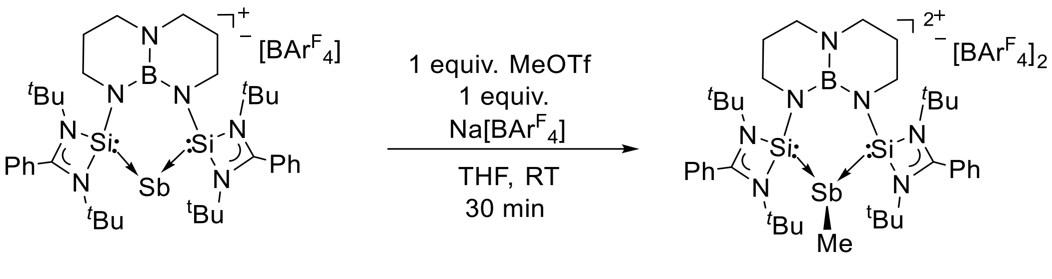

Supplementary Fig. 20. Synthesis of compound **4**.

^{1}H NMR (400 MHz, THF-d_8, 298 K): δ 7.78 (s, 16H, Barf-Ar-*H*), 7.72-7.70 (m, 4H, Ar-*H*), 7.65-7.61 (m, 6H, Ar-*H*), 7.57 (s, 8H, Barf-Ar-*H*), 3.42 (br, 4H, NCH_2), 3.08 (t, 4H, NCH_2, J = 6.3 Hz), 2.09 (br, 4H, CH_2-CH_2-CH_2),1.99 (s, 3H, Sb(CH_3), 1.32 (s, 18H, C(CH_3)$_3$), 1.29 (s, 18H, C(CH_3)$_3$).

^{13}C NMR (101 MHz, THF-d_8, 298 K): δ 181.5 (s, N*C*N), 162.6 (q, $J_{\text{C-B}}$= 49 Hz, Barf-Ar-*C*), 135.4 (s, (s, Ar*C*), 133.2 (s, Ar*C*), 130.0 (m, Barf-Ar-*C*), 129.7 (m, Barf-Ar-*C*), 129.6(s, Ar*C*), 129.4 (s, Ar*C*), 129.3(s, Ar*C*), 129.2(s, Ar*C*), 129.1 (s, Ar*C*), 128.1(s, Ar*C*), 125.3 (q, $J_{\text{C-F}}$ = 274 Hz, Barf-*C*F_3), 118.0 (m, Barf-Ar-*C*), 57.5 (s, N*C*(CH_3)$_3$), 56.4 (s, N*C*(CH_3)$_3$), 49.7 (s, N*C*H_2), 43.9 (s, N*C*H_2), 31.0(s, C(*C*H_3)$_3$), 30.7 (s, C(*C*H_3)$_3$), 28.4 (s, Sb(*C*H_3)), 27.5 (s, CH_2-*C*H_2-CH_2).

^{11}B NMR (128 MHz, THF-d_8, 298 K): δ 30.1 ppm (br), -6.5 ppm (s, Barf-*B*).

^{19}F NMR (377 MHz, THF-d_8, 298 K): δ -63.3 ppm (s).

^{29}Si NMR (79 MHz, THF-d_8, 298 K): δ -15.5 ppm (s).

Anal. Calcd for $C_{107}H_{90}B_3SbF_{49}N_7Si_2$: C, 49.14; H, 3.47; N, 3.75. Found: C, 48.89; H, 3.77; N, 3.34.

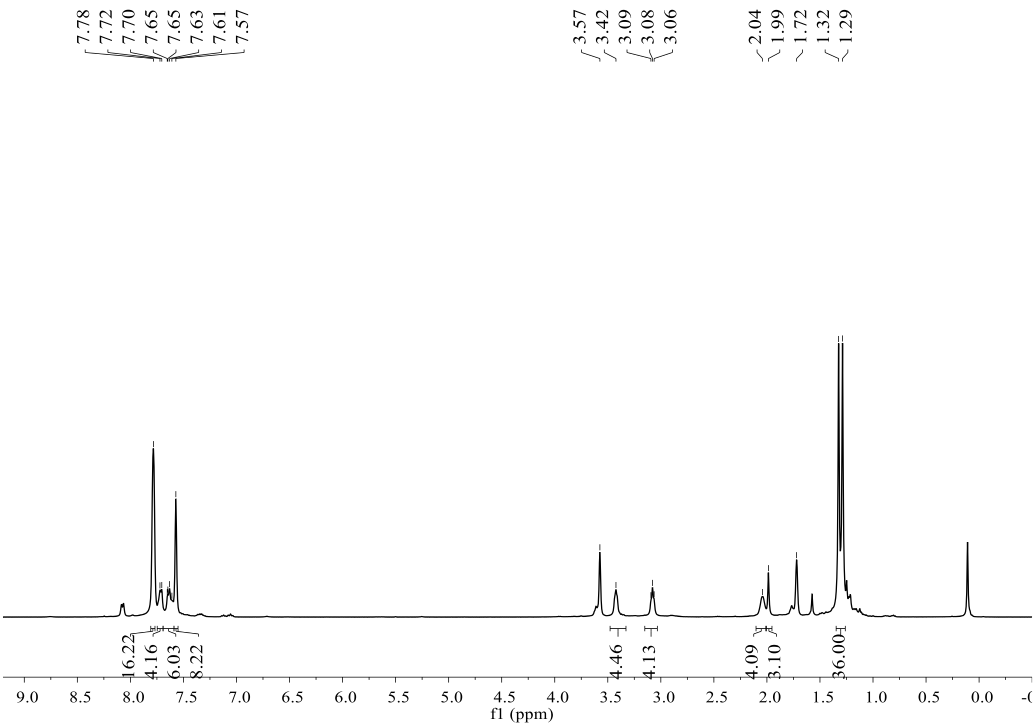

Supplementary Fig. 21. ^{1}H NMR spectrum of **4** in at THF-d_8 298 K.

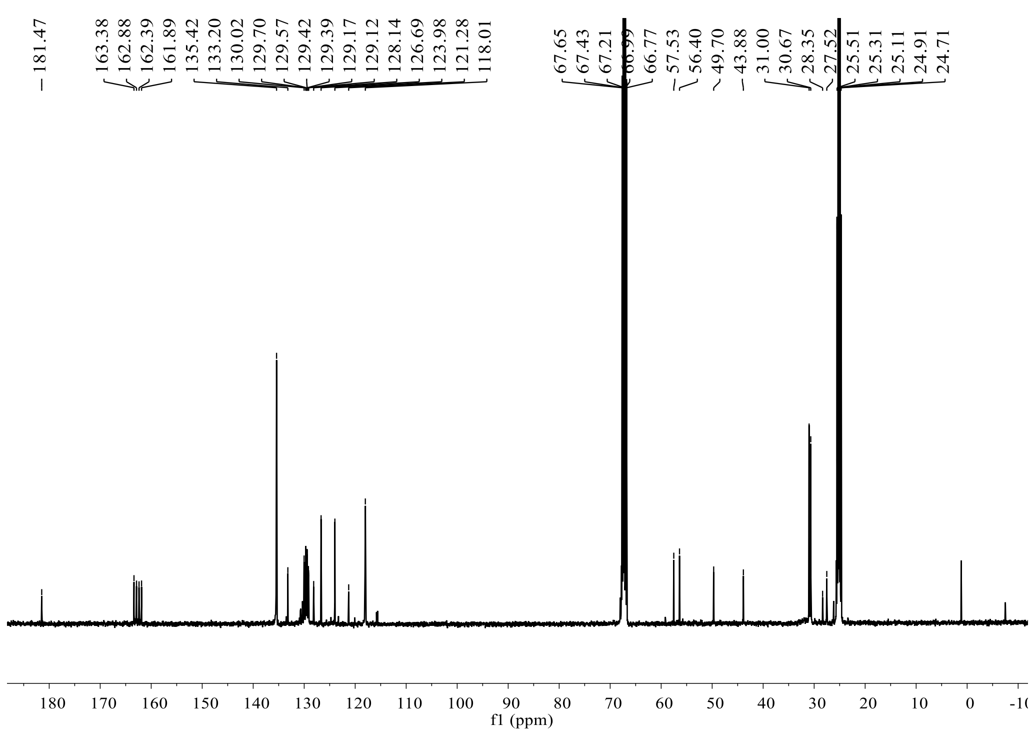

Supplementary Fig. 22. ^{13}C NMR spectrum of **4** in THF-d_8 at 298 K.

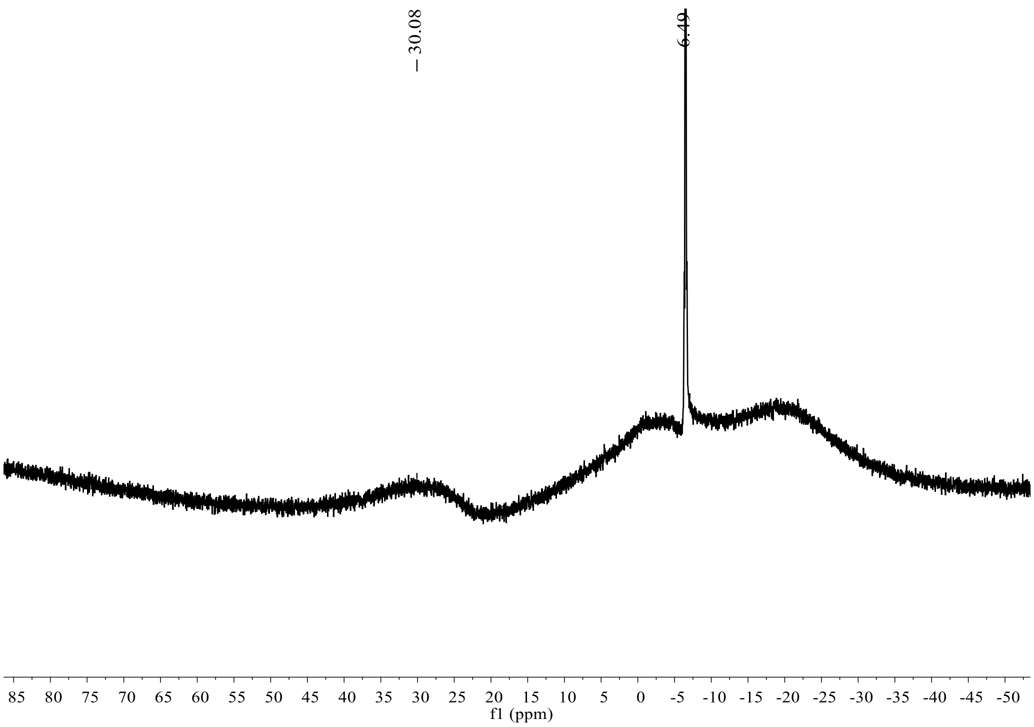

Supplementary Fig. 23. ^{11}B NMR spectrum of **4** in THF-d_8 at 298 K.

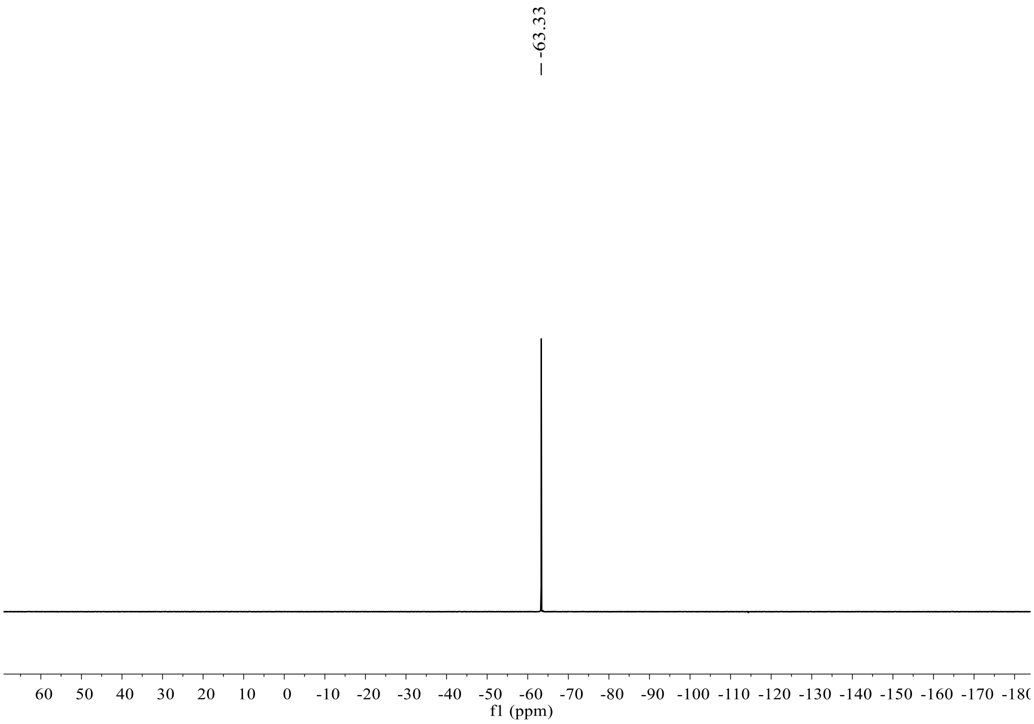

Supplementary Fig. 24. ^{19}F NMR spectrum of **4** in THF-d_8 at 298 K.

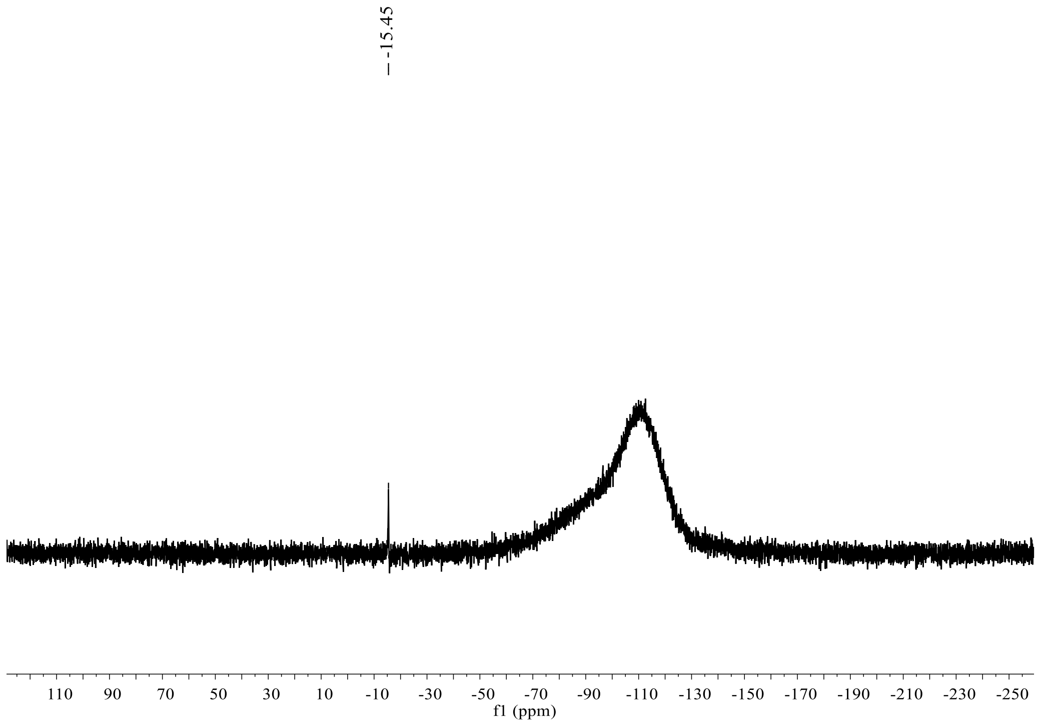

Supplementary Fig. 25. ^{29}Si NMR spectrum of **4** in THF-d_8 at 298 K.

Preparation of Compound 5: MeOTf (9.7 μL, 0.092 mmol) was added to **3** (160 mg, 0.092 mmol) in fluorobenzene (10 mL) at -30 °C. Then Na[BAr^F$_4$] (82 mg, 0.092 mmol) was added to this mixture immediately. The solution was stirred for 30 minutes and the color changed from yellow to white. The reaction solution was diluted with 5 mL of hexane with rigorous stirring to give a white precipitate. After decanting the supernatant, the precipitate was washed with diethyl ether (6 mL) and dried under vacuum to give **5** as a colourless, moisture sensitive solid (177 mg, 0.065 mmol, 71% yield). Single crystals suitable for X-ray diffraction studies were obtained by slow diffusion of hexane into the saturated fluorobenzene solutions of **5** at 5 °C.

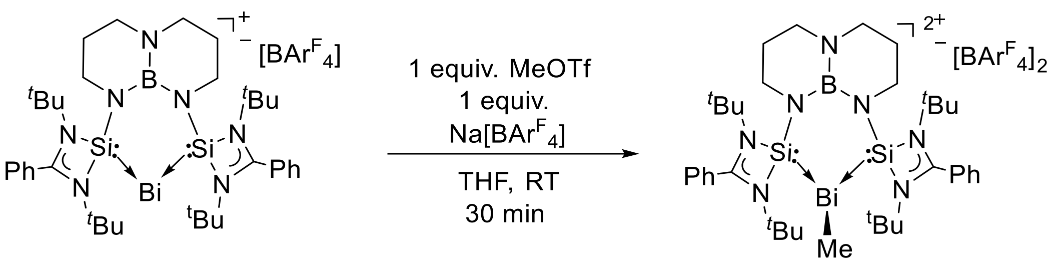

Supplementary Fig. 26. Synthesis of compound **5**.

^{1}H NMR (400 MHz, THF-d_8, 298 K): δ 7.79 (s, 16H, Barf-Ar-*H*), 7.75-7.65 (m, 10H, Ar-*H*), 7.58 (s, 8H, Ar-*H*), 3.47 (t, 4H, NCH_2, J = 4.8 Hz), 3.09 (m, 4H, NCH_2), 2.25 (s, 3H, Bi(CH_3)), 2.09 (m, 4H, CH_2-CH_2-CH_2), 1.31 (s, 18H, C(CH_3)$_3$), 1.30 (s, 18H, C(CH_3)$_3$).

^{13}C NMR (101 MHz, THF-d_8, 298 K): δ 179.7 (s, N*C*N), 162.6 (q, $J_{C\text{-}B}$= 51 Hz, Barf-Ar-*C*), 135.4 (s, Ar*C*), 132.9 (s, Ar*C*), 130.2 (m, Barf-Ar-*C*), 129.8. (s, Ar*C*), 129.7 (m, Barf-Ar-*C*), 129.6. (s, Ar*C*), 129.5 (s, Ar*C*), 129.3 (s, Ar*C*), 128.9 (s, Ar*C*), 128.8 (s, Ar*C*), 125.3 (q, $J_{C\text{-}F}$ = 273 Hz, Barf-*C*F_3), 118.0 (m, Barf-Ar-*C*), 57.0 (s, N*C*(CH_3)$_3$), 56.0 (s, N*C*(CH_3)$_3$), 49.9 (s, N*C*H_2), 44.1 (s, N*C*H_2), 31.3(s, C(*C*H_3)$_3$), 30.6 (s, C(*C*H_3)$_3$), 28.4 (s, Bi(*C*H_3)), 27.8 (s, CH_2-*C*H_2-CH_2),

^{11}B NMR (128 MHz, THF-d_8, 298 K): δ 29.3 ppm (br), -6.5 ppm (s, Barf-*B*).

^{19}F NMR (377 MHz, THF-d_8, 298 K): δ -65.2 ppm (s).

^{29}Si NMR (79 MHz, THF-d_8, 298 K): δ -31.2 ppm (s).

Anal. Calcd for $C_{107}H_{90}B_3BiF_{49}N_7Si_2$: C, 47.56; H, 3.36; N, 3.63. Found: C, 47.12; H, 3.70; N, 3.33.

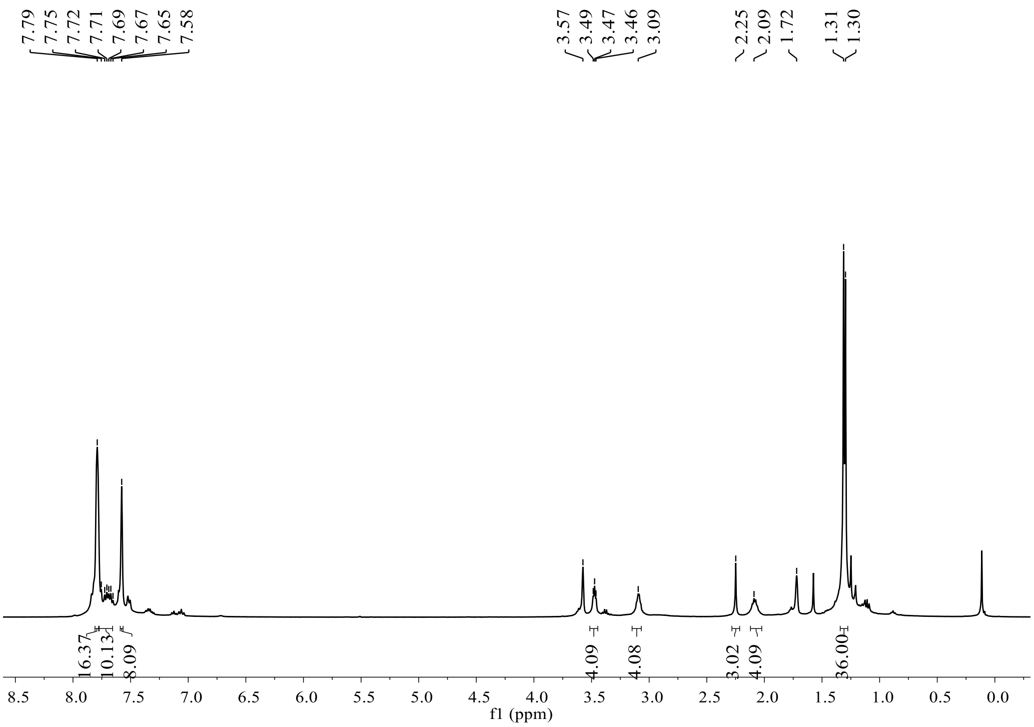

Supplementary Fig. 27. ^{1}H NMR spectrum of **5** in THF-d_8 at 298 K.

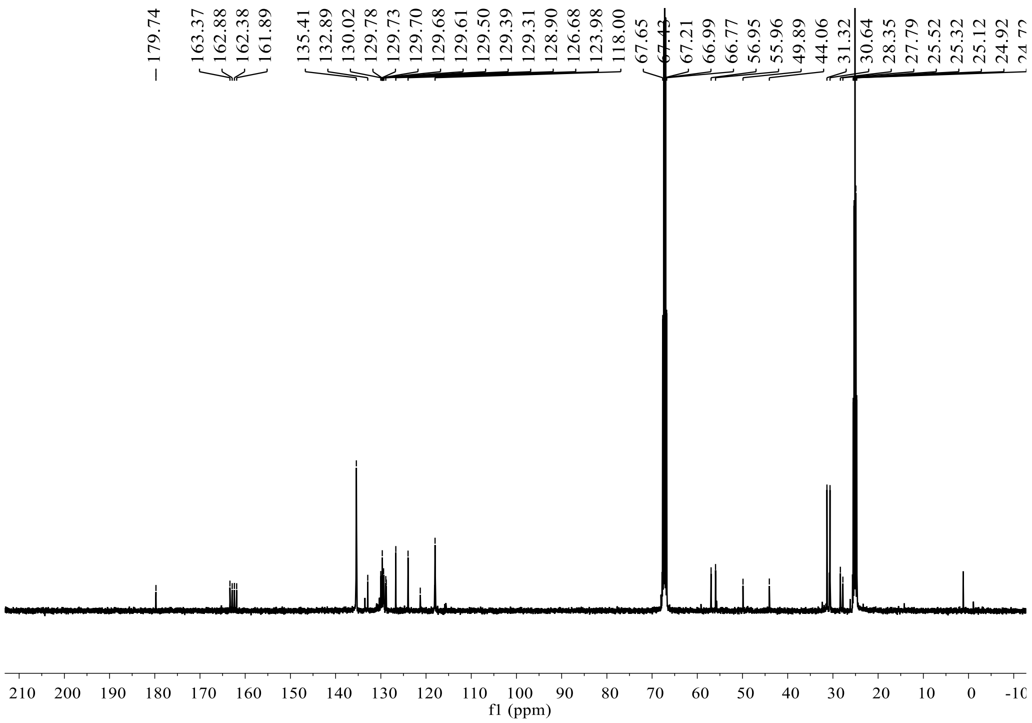

Supplementary Fig. 28. ^{13}C NMR spectrum of **5** in THF-d_8 at 298 K.

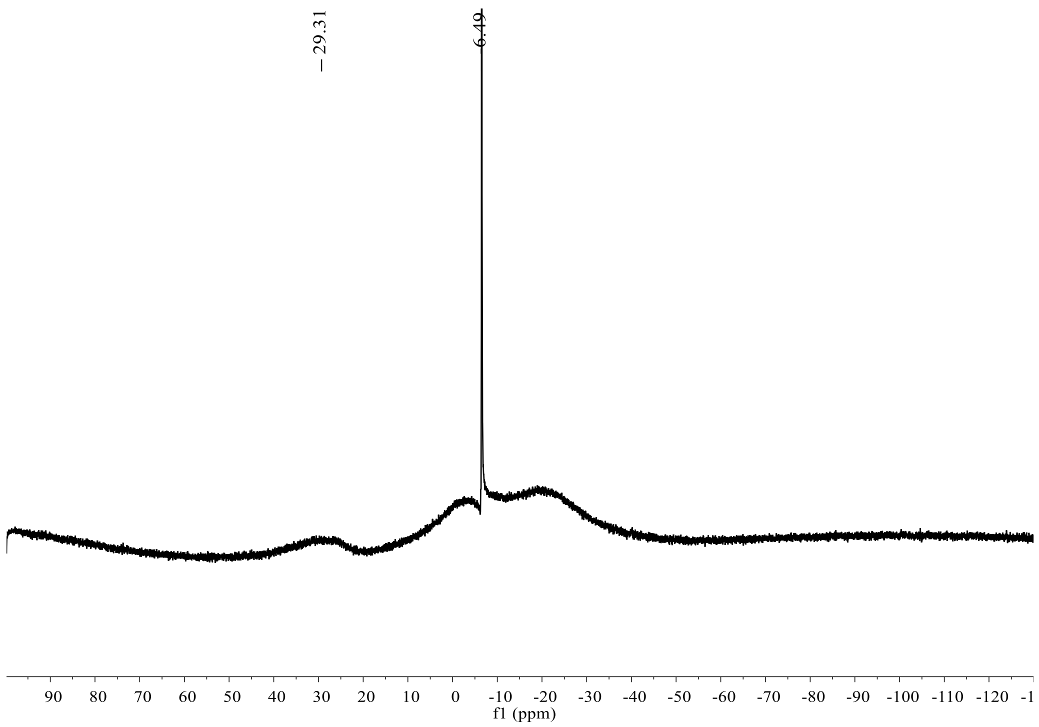

Supplementary Fig. 29. ^{11}B NMR spectrum of **5** in THF-d_8 at 298 K.

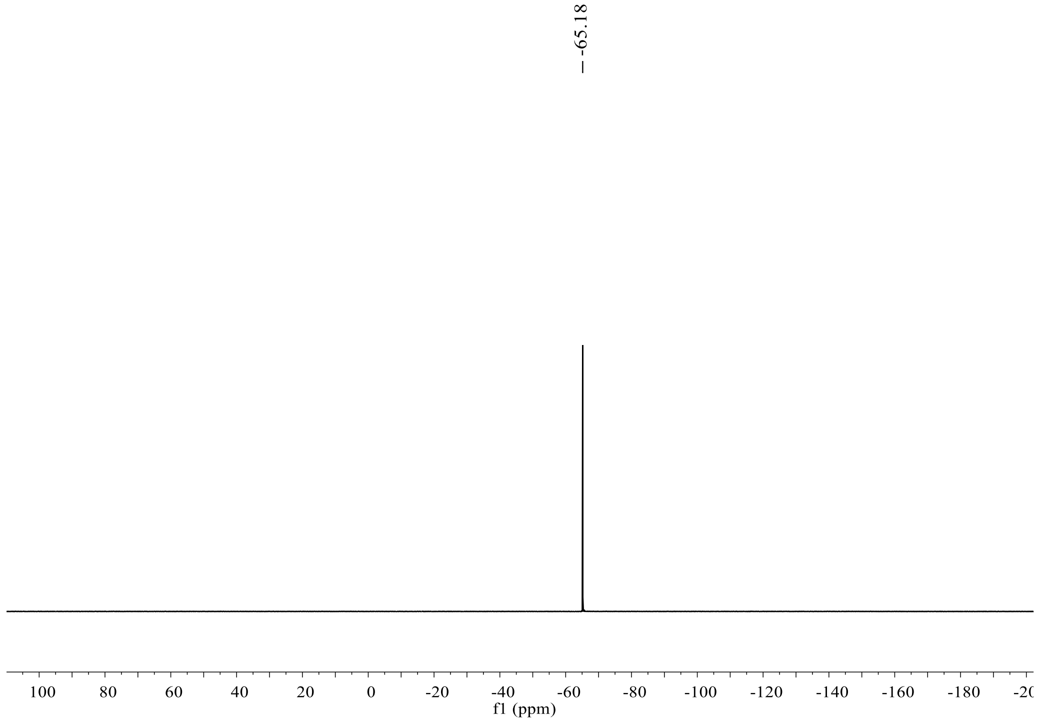

Supplementary Fig. 30. ^{19}F NMR spectrum of **5** in THF-d_8 at 298 K.

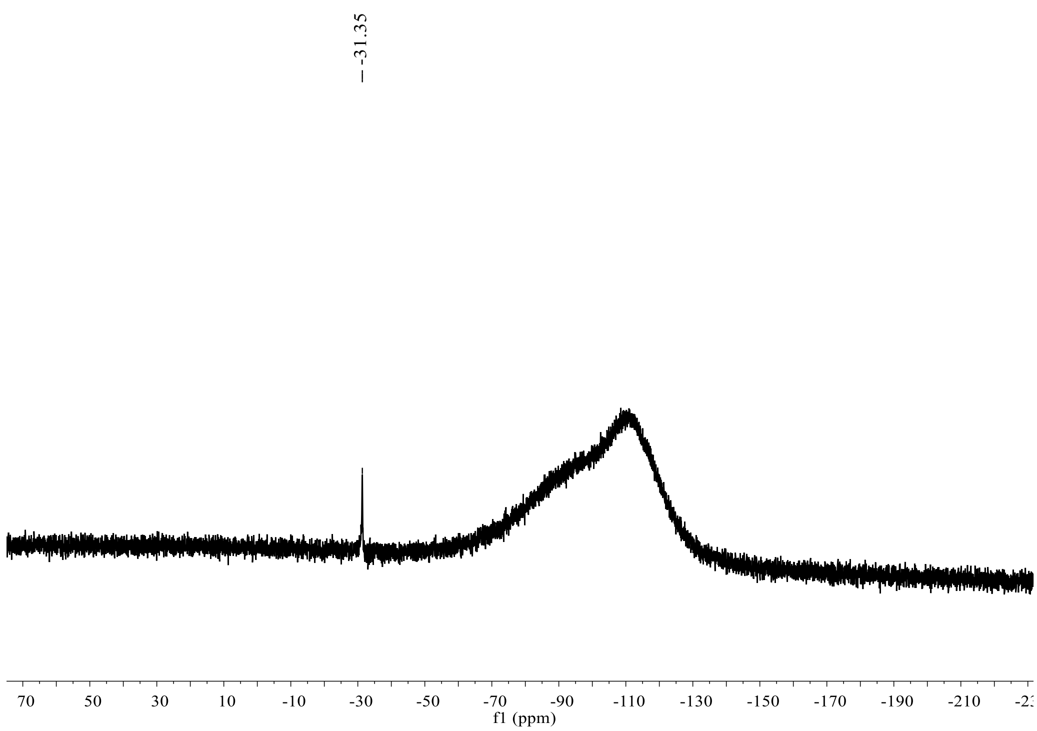

Supplementary Fig. 31. ^{29}Si NMR spectrum of **5** in THF-d_8 at 298 K.

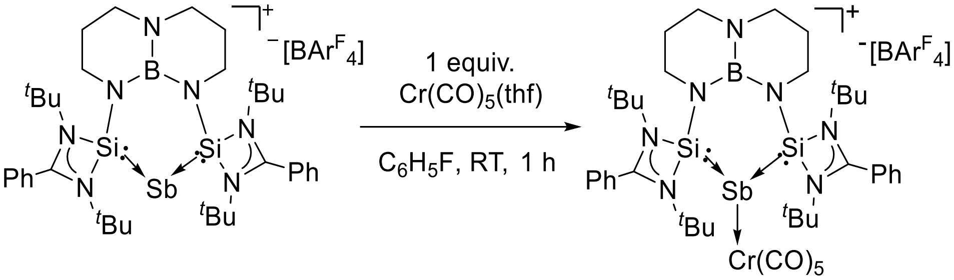

Supplementary Fig. 32. Synthesis of compound **6**.

Preparation of Compound 6: A sample of [$Cr(CO)_6$] (13 mg, 0.06 mmol) in THF (6 mL) was irradiated by UV-lamp for 1 h to generate yellow solution of [$Cr(CO)_5$(thf)], that was then added to a solution of complex **2** (100 mg, 0.06 mmol) in fluorobenzene (5 mL) at room temperature The resulting yellow solution was stirred for a further 1 h at room temperature. The solvent was removed in vacuum, and the residue was washed with hexane (10 mL) and extracted with Et_2O (5 mL). The solvent was evaporated to yield compound **6** as a yellow powder (88 mg, 0.048 mmol, 80%). Single crystals

suitable for X-ray diffraction studies were obtained by slow diffusion of hexane to the saturated fluorobenzene solutions at RT.

^{1}H NMR (400 MHz, $CDCl_3$, 298 K): δ 7.71 (s, 8H, Barf-Ar-*H*), 7.62-7.60 (m, 2H, Ar-*H*), 7.52-7.44 (m, 10H, Ar-*H*), 7.35-7.34 (m, 2H, Ar-*H*), 3.23 (br, 4H, NCH_2), 2.93 (br, 4H, NCH_2), 1.92 (br, 4H, CH_2-CH_2-CH_2), 1.27 (s, 36H, $C(CH_3)_3$)

^{13}C NMR (101 MHz, $CDCl_3$, 298 K): 222.7 (s, *C*O-ax.), 219.1 (s, *C*O-eq.) δ 178.3 (s, N*C*N), 160.7 (q, $J_{C\text{-}B}$= 51 Hz, Barf-Ar-*C*), 133.8 (s, Ar*C*), 131.2 (s, Ar*C*), 128.1 (s, Ar*C*), 127.8 (m, Barf-Ar-*C*), 127.7 (m, Barf-Ar-*C*), 127.6 (s, Ar*C*), 127.5 (s, Ar*C*), 127.4 (s, Ar*C*), 126.9 (s, Ar*C*), 125.7 (s, Ar*C*), 123.5 (q, $J_{C\text{-}F}$ = 274 Hz, Barf-CF_3), 116.5 (m, Barf-Ar-*C*), 55.2 (s, $NC(CH_3)_3$), 47.9 (s, NCH_2), 41.4 (s, NCH_2), 29.9. (s, $C(CH_3)_3$), 25.8 (s, CH_2-CH_2-CH_2).

^{11}B NMR (128 MHz, $CDCl_3$, 298 K): δ 29.0 ppm (br), -6.6 ppm (s, Barf-*B*).

^{19}F NMR (377 MHz, $CDCl_3$, 298 K): δ -62.4 ppm (s).

^{29}Si NMR (79 MHz, $CDCl_3$, 298 K): δ -15.6 ppm (s).

IR (CO, cm^{-1}): 2045, 1967, 1930, and 1909 cm^{-l}.

Anal. Calcd for $C_{73}H_{70}B_2CrF_{24}N_7O_5SbSi_2$: C, 47.84; H, 3.85; N, 5.35. Found: C, 47.52; H, 3.60; N, 4.93.

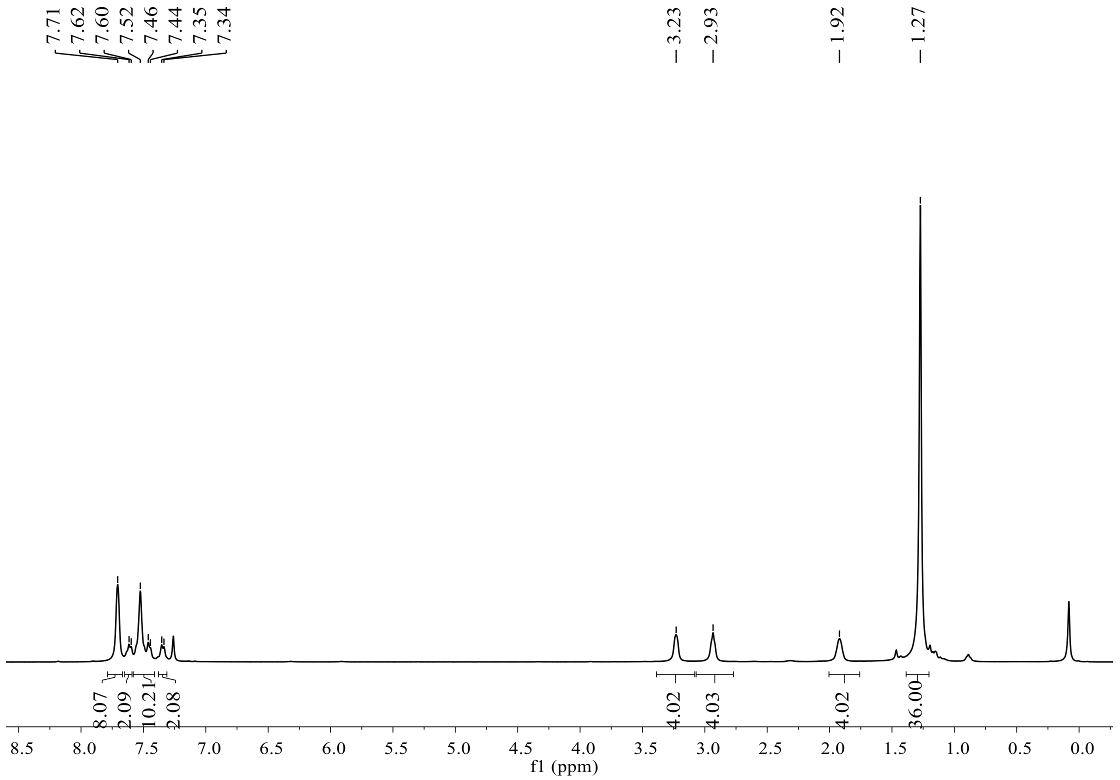

Supplementary Fig. 33.^{1}H NMR spectrum of **6** in $CDCl_3$ at 298 K.

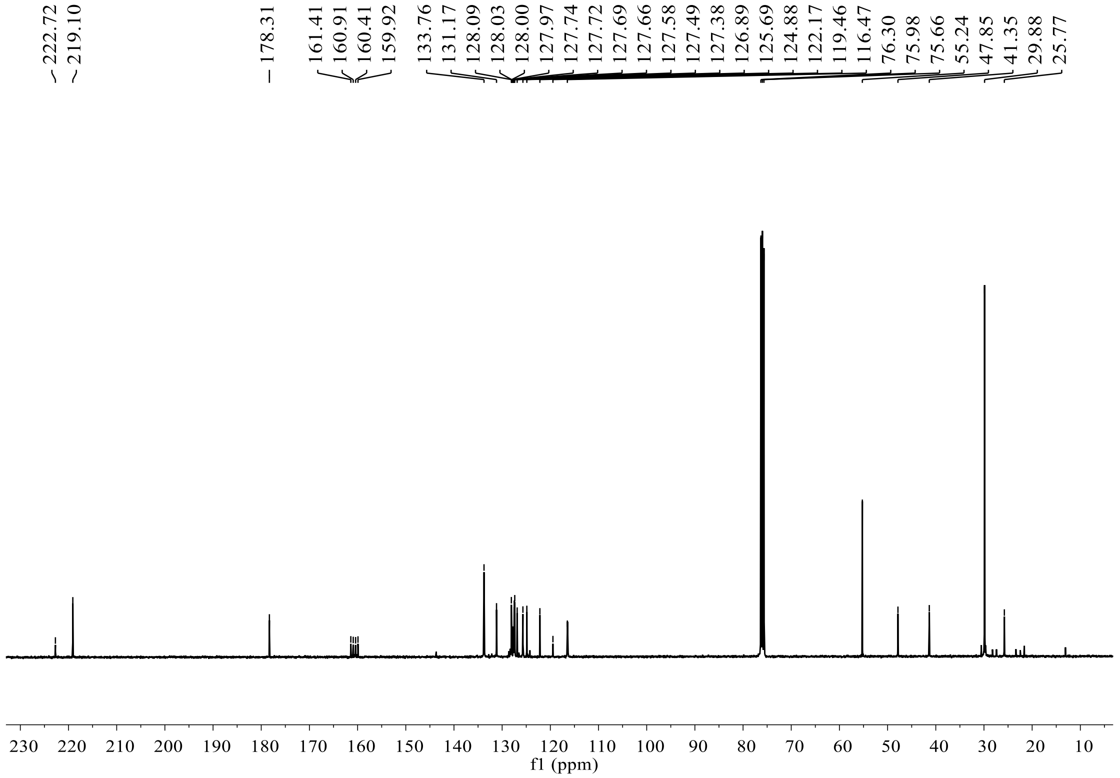

Supplementary Fig. 34. ^{13}C NMR spectrum of **6** in $CDCl_3$ at 298 K.

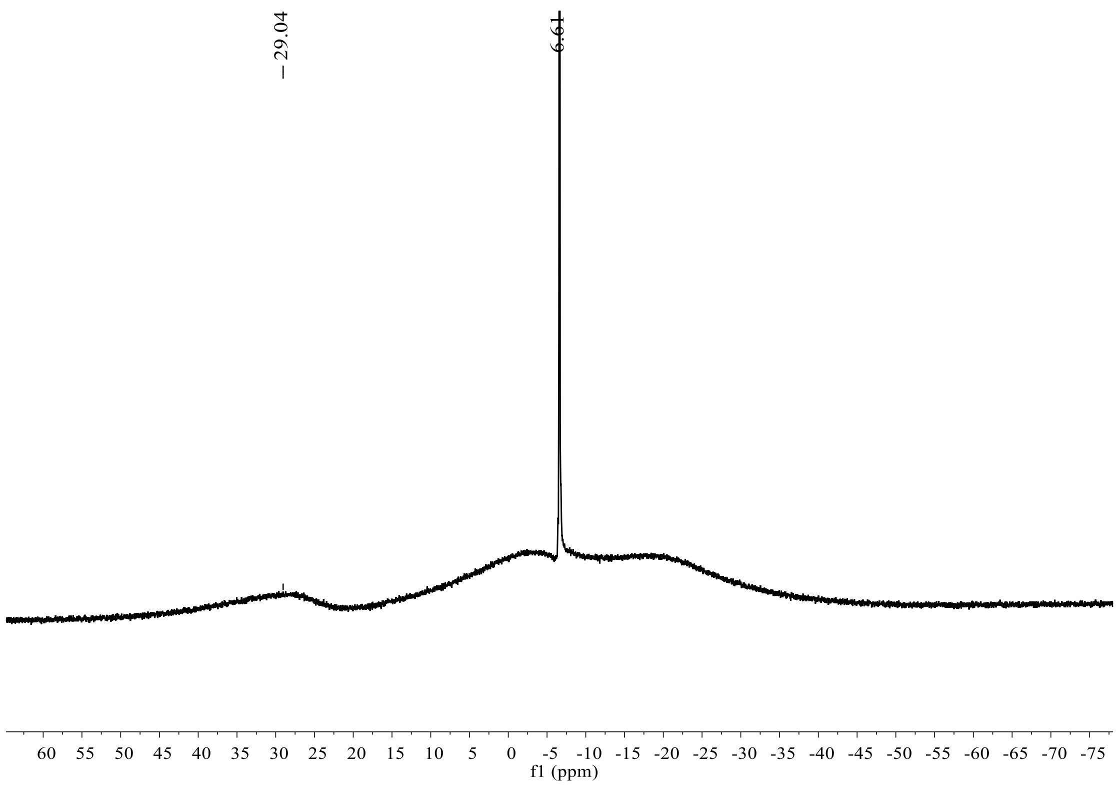

Supplementary Fig. 35. ^{11}B NMR spectrum of **6** in $CDCl_3$ at 298 K.

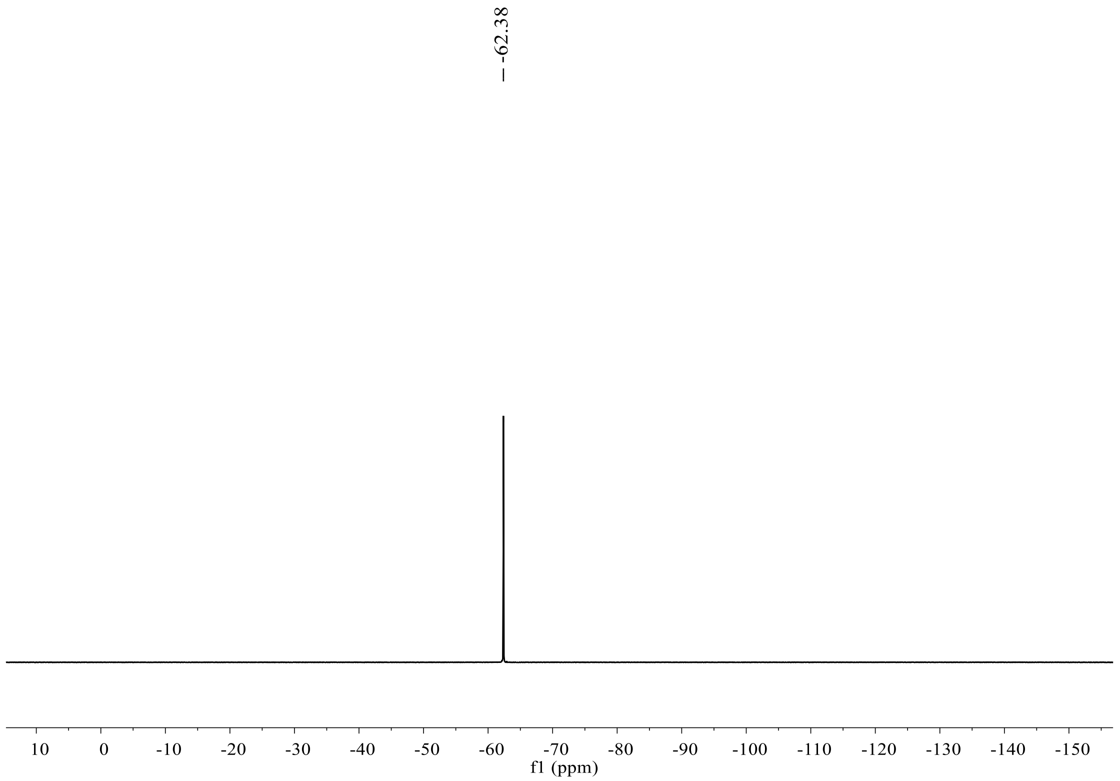

Supplementary Fig. 36. ^{19}F NMR spectrum of **6** in $CDCl_3$ at 298 K.

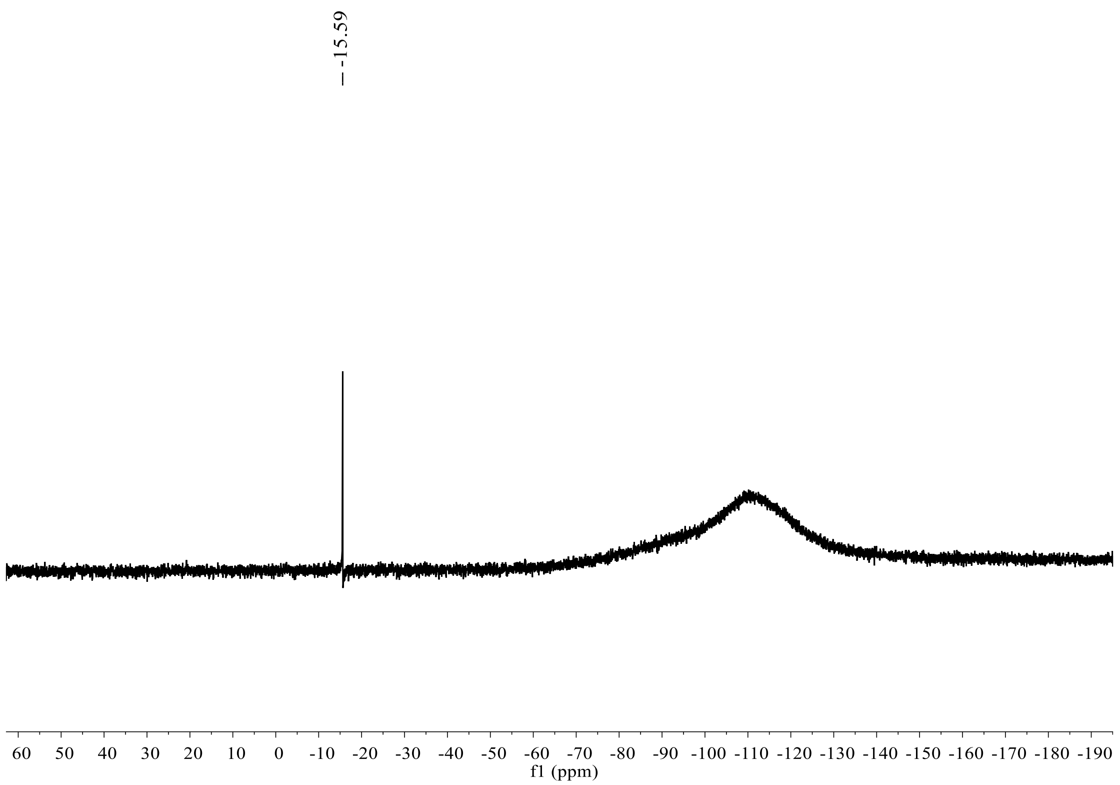

Supplementary Fig. 37. ^{29}Si NMR spectrum of **6** in $CDCl_3$ at 298 K.

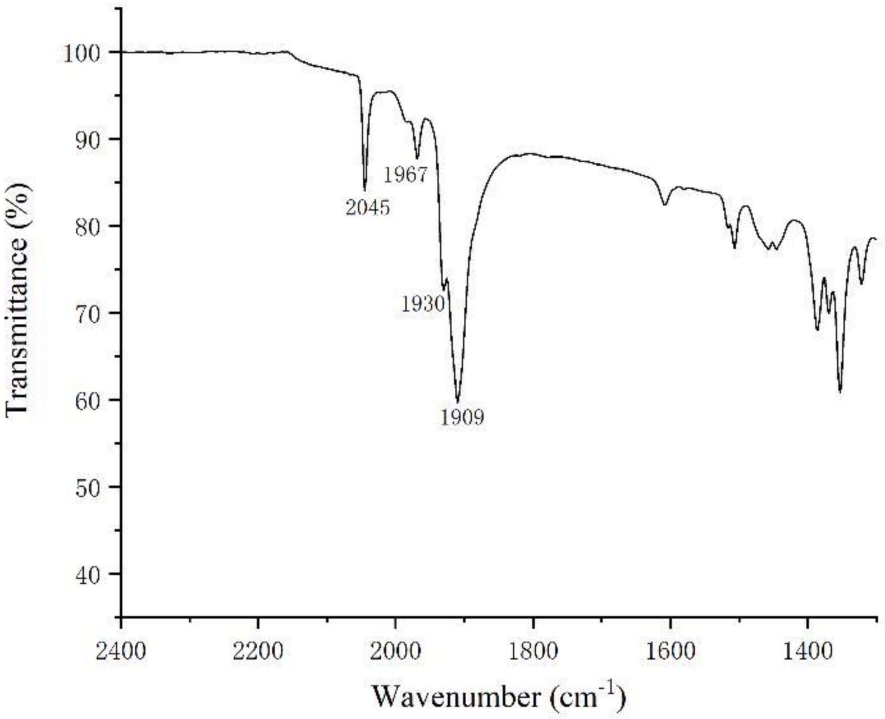

Supplementary Fig. 38. IR spectrum of **6**.

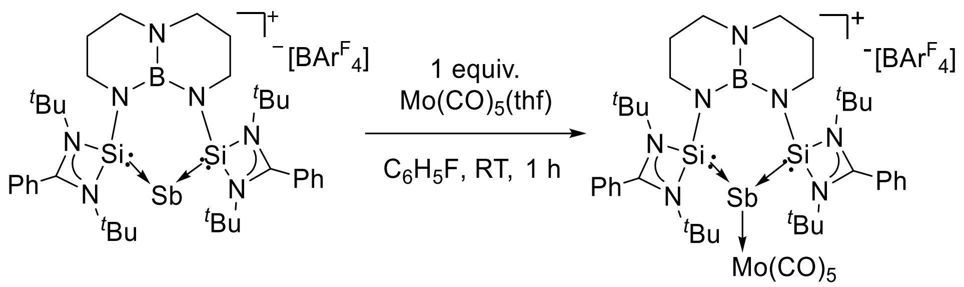

Supplementary Fig. 39. Synthesis of compound **7**.

Preparation of Compound 7: A sample of Mo[$(CO)_6$] (16 mg, 0.06 mmol) in THF (6 mL) was irradiated by UV-lamp for 1 h to generate yellow solution of [$Mo(CO)_5$(thf)], that was then added to a solution of complex **2** (100 mg, 0.06 mmol) in fluorobenzene (5 mL) at room temperature The resulting yellow solution was stirred for a further 1 h at room temperature. The solvent was removed in vacuum, and the residue was washed with hexane (10 mL) and extracted with Et_2O (5 mL). The solvent was evaporated to yield compound **7** as a yellow powder (81 mg, 0.043 mmol, 71%). Single crystals suitable for X-ray diffraction studies were obtained by slow diffusion of hexane to the saturated fluorobenzene solutions at RT.

^{1}H NMR (400 MHz, $CDCl_3$, 298 K): δ 7.70 (s, 8H, Barf-Ar-*H*), 7.63-7.60 (m, 2H, Ar-*H*), 7.56-7.45 (m, 10H, Ar-*H*), 7.38-7.33 (m, 2H, Ar-*H*), 3.23 (br, 4H, NCH_2), 2.93 (br, 4H, NCH_2), 1.92 (br, 4H, CH_2-CH_2-CH_2), 1.27 (s, 36H, C(CH_3)$_3$)

^{13}C NMR (101 MHz, $CDCl_3$, 298 K): 210.5 (s, *C*O-ax.), 208.0 (s, *C*O-eq.) δ 178.9 (s, N*C*N), 161.7 (q, $J_{C\text{-}B}$= 49 Hz, Barf-Ar-*C*), 134.8 (s, Ar*C*), 132.2 (s, Ar*C*), 129.0 (m, Barf-Ar-*C*), 128.7 (m, Barf-Ar-*C*), 128.6 (s, Ar*C*), 128.5 (s, Ar*C*), 128.4 (s, Ar*C*), 128.0 (s, Ar*C*), 126.5 (s, Ar*C*), 125.7 (s, Ar*C*), 124.5 (q, $J_{C\text{-}F}$ = 274 Hz, Barf-*C*F_3), 117.5 (m, Barf-Ar-*C*), 56.3 (s, N*C*(CH_3)$_3$), 48.9 (s, N*C*H_2), 42.4 (s, N*C*H_2), 30.9. (s, C(*C*H_3)$_3$), 26.8 (s, CH_2-*C*H_2-CH_2).

^{11}B NMR (128 MHz, $CDCl_3$, 298 K): δ 29.3 ppm (br), -6.6 ppm (s, Barf-*B*).

^{19}F NMR (377 MHz, $CDCl_3$, 298 K): δ -62.4 ppm (s).

^{29}Si NMR (79 MHz, $CDCl_3$, 298 K): δ -15.1 ppm (s).

IR (CO, cm^{-1}): 2063, 1987, 1936, and 1901 cm^{-1}.

Anal. Calcd for $C_{73}H_{70}B_2MoF_{24}N_7O_5SbSi_2$: C, 46.72.; H, 3.76; N, 5.22. Found: C, 46.31; H, 3.15; N, 4.93.

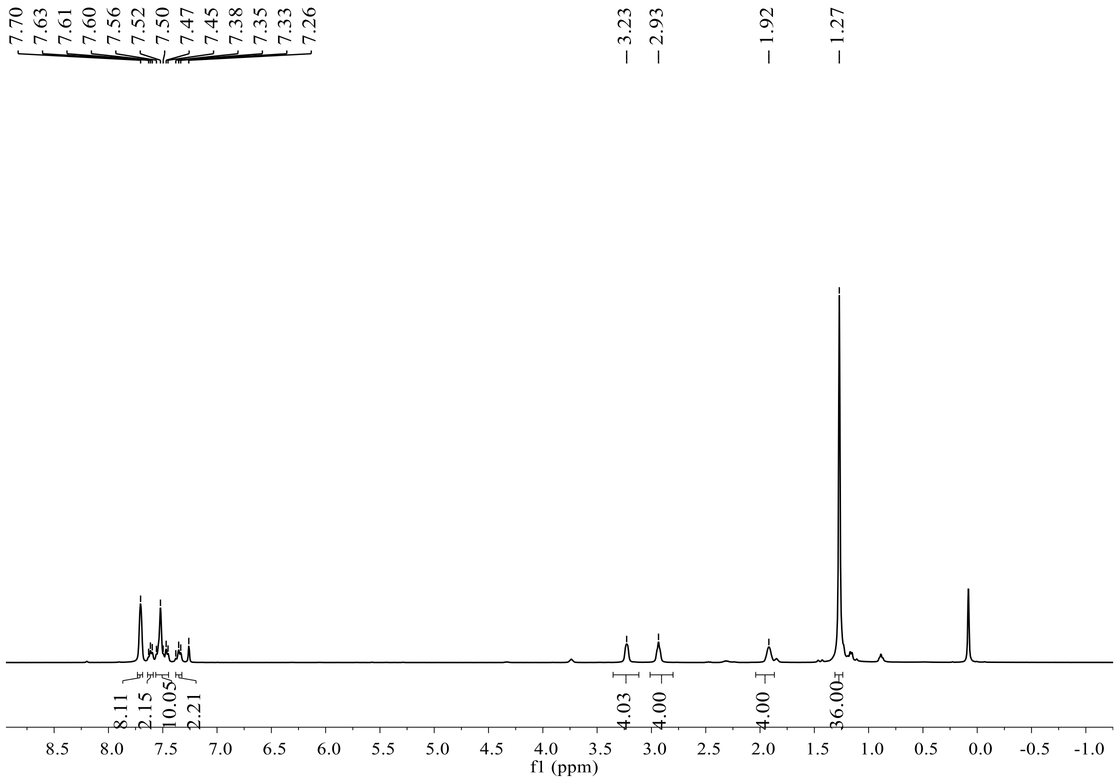

Supplementary Fig. 40. ^{1}H NMR spectrum of **7** in $CDCl_3$ at 298 K.

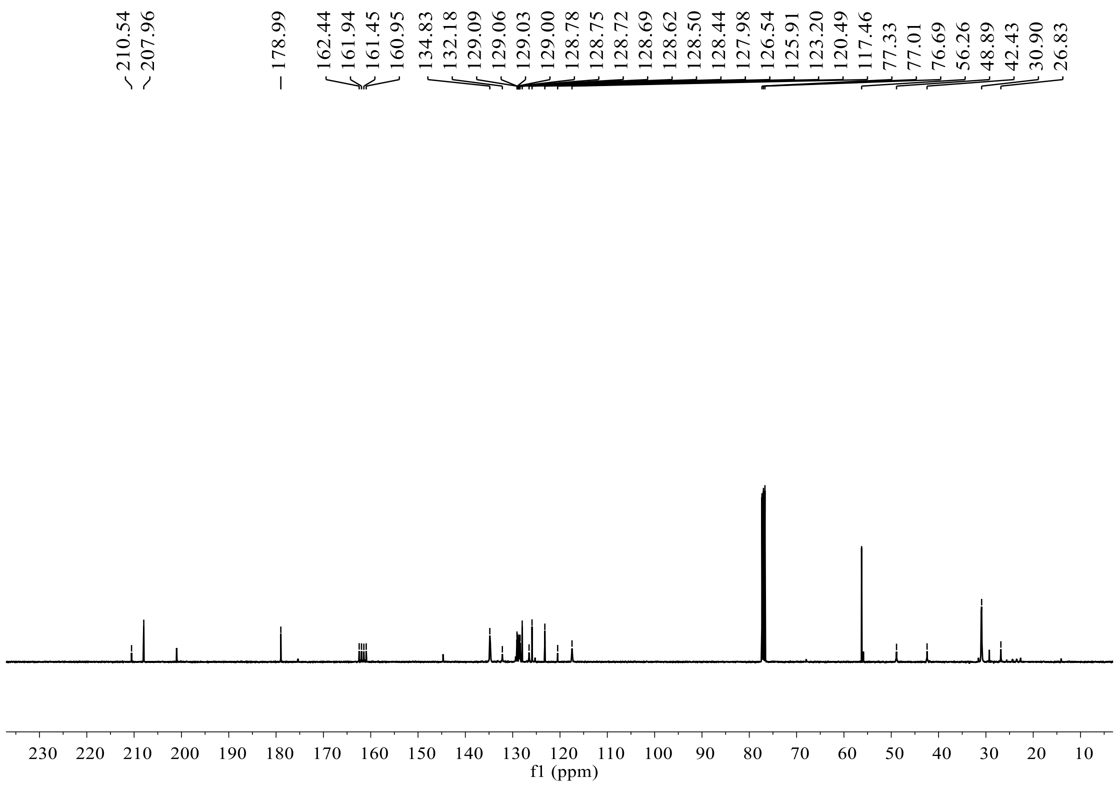

Supplementary Fig. 41. ^{13}C NMR spectrum of **7** in $CDCl_3$ at 298 K.

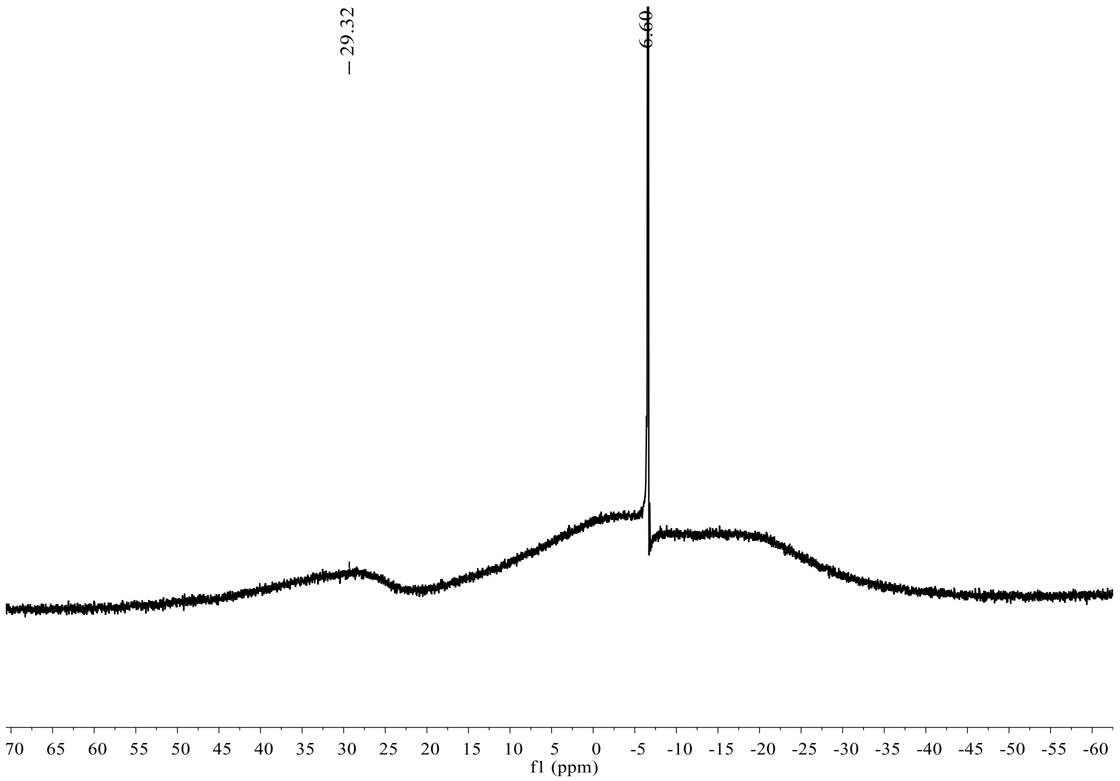

Supplementary Fig. 42. ^{11}B NMR spectrum of **7** in $CDCl_3$ at 298 K.

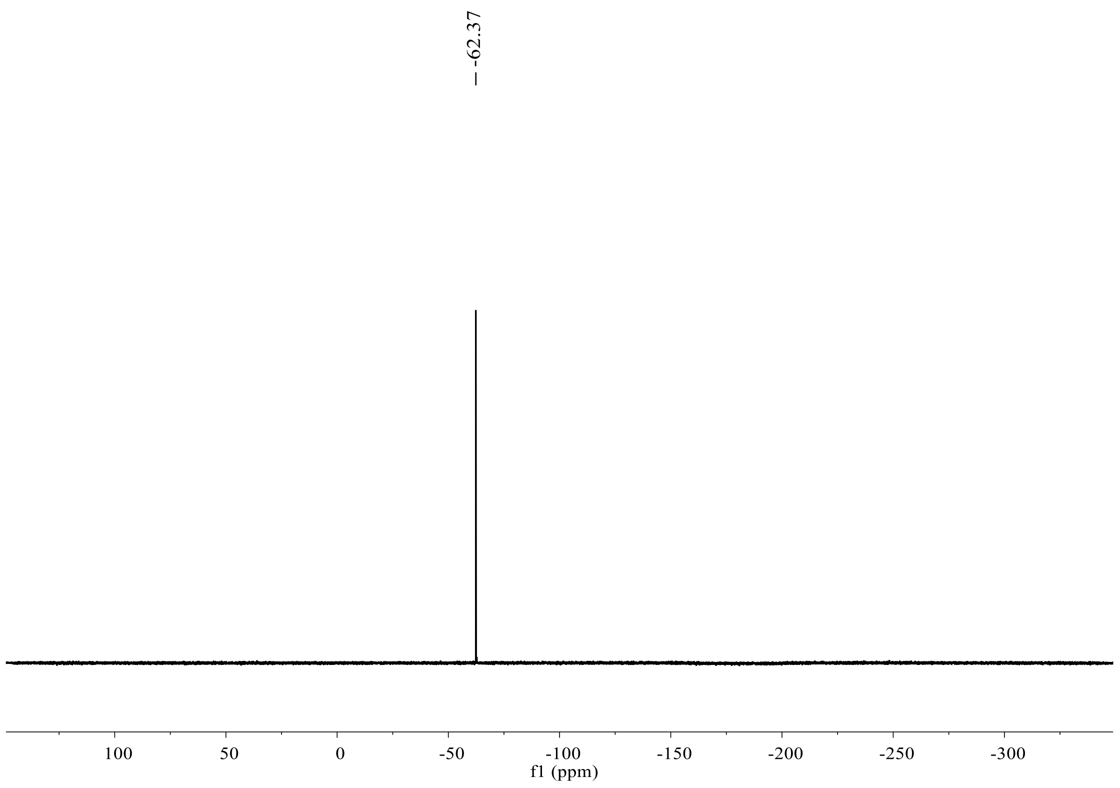

Supplementary Fig. 43. ^{19}F NMR spectrum of **7** in $CDCl_3$ at 298 K.

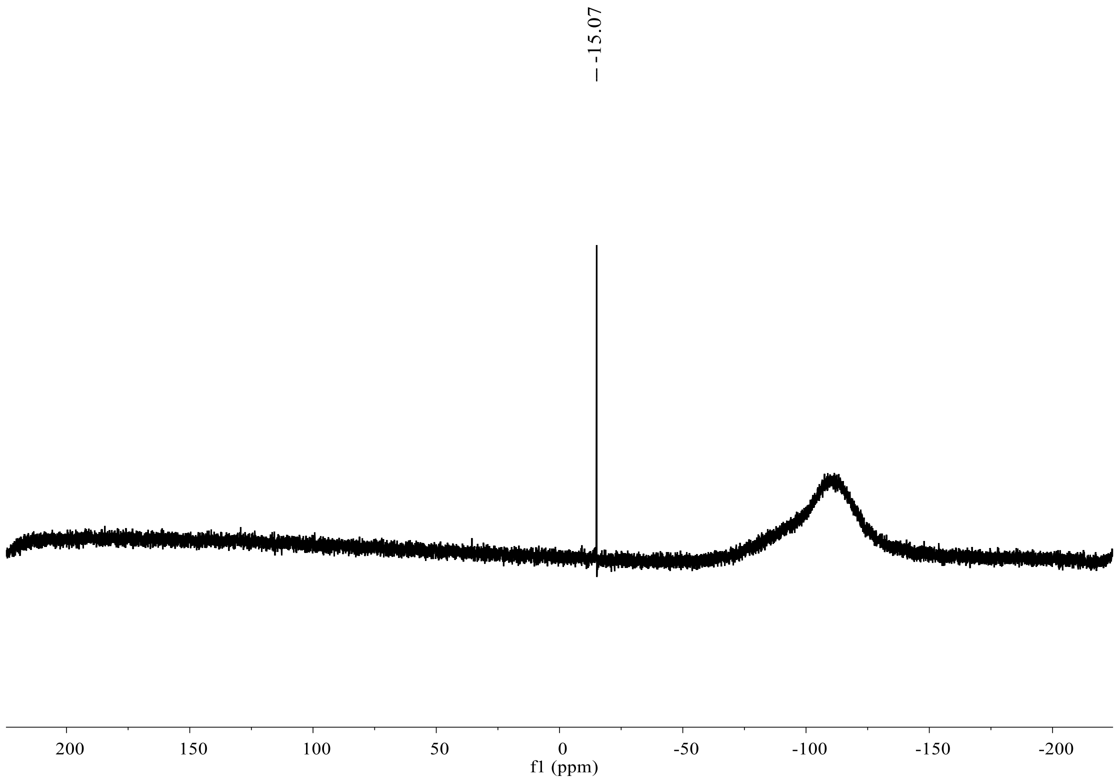

Supplementary Fig. 44. ^{29}Si NMR spectrum of **7** in $CDCl_3$ at 298 K.

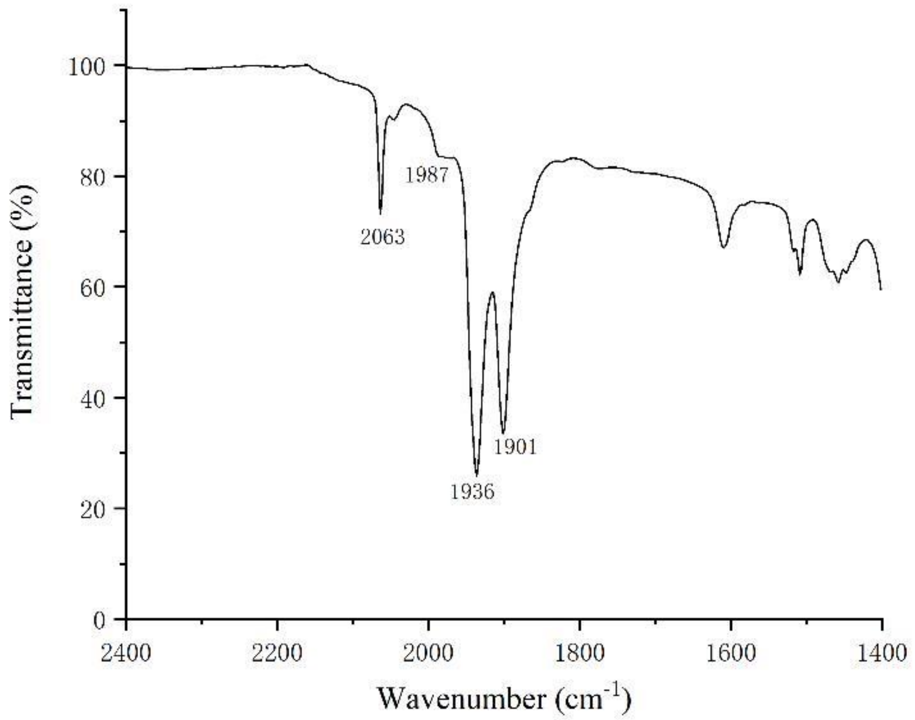

Supplementary Fig. 45 IR spectrum of **7**.

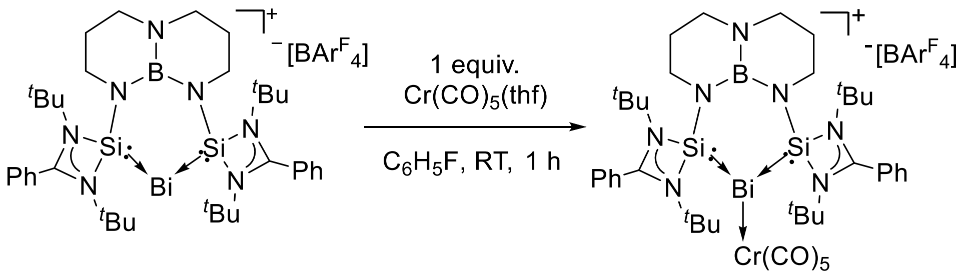

Supplementary Fig. 46. Synthesis of compound **8**.

Preparation of Compound 8: A sample of [$Cr(CO)_6$] (13 mg, 0.06 mmol) in THF (6 mL) was irradiated by UV-lamp for 1 h to generate yellow solution of [$Cr(CO)_5$(thf)], that was then added to a solution of complex **3** (100 mg, 0.06 mmol) in fluorobenzene (5 mL) at room temperature The resulting yellow solution was stirred for a further 1 h at room temperature. The solvent was removed in vacuum, and the residue was washed

with hexane (10 mL) and extracted with Et_2O (5 mL). The solvent was evaporated to yield compound **8** as a yellow powder (85 mg, 0.043 mmol, 75%). Single crystals suitable for X-ray diffraction studies were obtained by slow diffusion of hexane to the saturated fluorobenzene solutions at RT.

^{1}H NMR (400 MHz, $CDCl_3$, 298 K): δ 7.71 (s, 8H, Barf-Ar-*H*), 7.63-7.60 (m, 2H, Ar-*H*), 7.53-7.49 (m, 8H, Ar-*H*), 7.44-7.42 (m, 2H, Ar-*H*), 7.38-7.36 (m, 2H, Ar-*H*), 3.24 (br, 4H, NC*H2*), 2.93 (br, 4H, NC*H2*), 1.92 (br, 4H, CH_2-C*H2*-CH_2), 1.26 (s, 36H, C(C*H3*)3)

^{13}C NMR (101 MHz, $CDCl_3$, 298 K): 220.9 (s, *C*O-ax.), 220.8 (s, *C*O-eq.) δ 177.0 (s, N*C*N), 160.1 (q, $J_{C\text{-}B}$= 49 Hz, Barf-Ar-*C*), 133.8 (s, Ar*C*), 131.1 (s, Ar*C*), 128.1 (s, Ar*C*), 128.0 (m, Barf-Ar-*C*), 127.8 (s, Ar*C*), 127.7 (m, Barf-Ar-*C*), 127.6 (s, Ar*C*), 127.4 (s, Ar*C*), 127.2 (s, Ar*C*), 126.2 (s, Ar*C*), 123.5 (q, $J_{C\text{-}F}$ = 274 Hz, Barf-*C*F_3), 116.5 (m, Barf-Ar-*C*), 55.0 (s, N*C*(CH_3)3), 48.0 (s, N*C*H_2), 41.4 (s, N*C*H_2), 30.2. (s, C(*C*H_3)3), 26.0 (s, CH_2-*C*H_2-CH_2).

^{11}B NMR (128 MHz, $CDCl_3$, 298 K): δ 28.7 ppm (br), -6.6 ppm (s, Barf-*B*).

^{19}F NMR (377 MHz, $CDCl_3$, 298 K): δ -62.4 ppm (s).

^{29}Si NMR (79 MHz, $CDCl_3$, 298 K): δ -81.6 ppm (s).

IR (CO, cm-1): 2037, 1963, 1928, and 1905 cm^{-1}.

Anal. Calcd for $C_{73}H_{70}B_2CrF_{24}N7O_5BiSi_2$: C, 45.66; H, 3.67; N, 5.11. Found: C, 46.12; H, 3.40; N, 5.53.

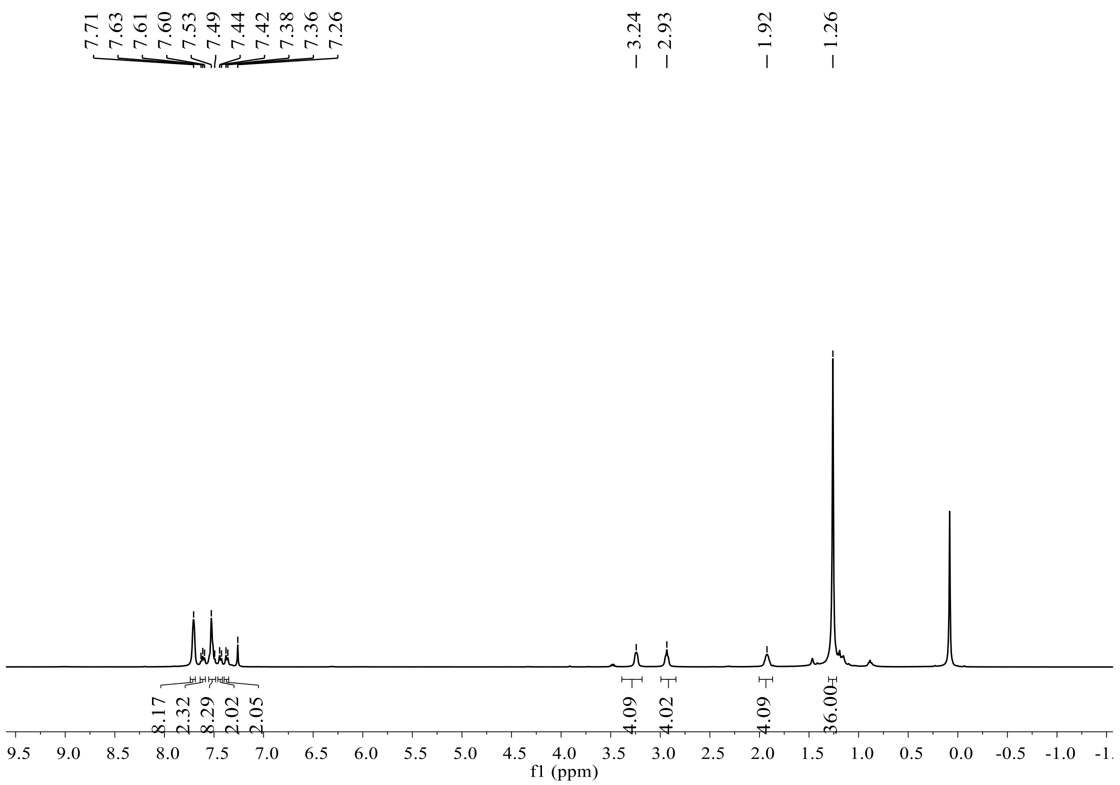

Supplementary Fig. 47. ^{1}H NMR spectrum of **8** in $CDCl_3$ at 298 K.

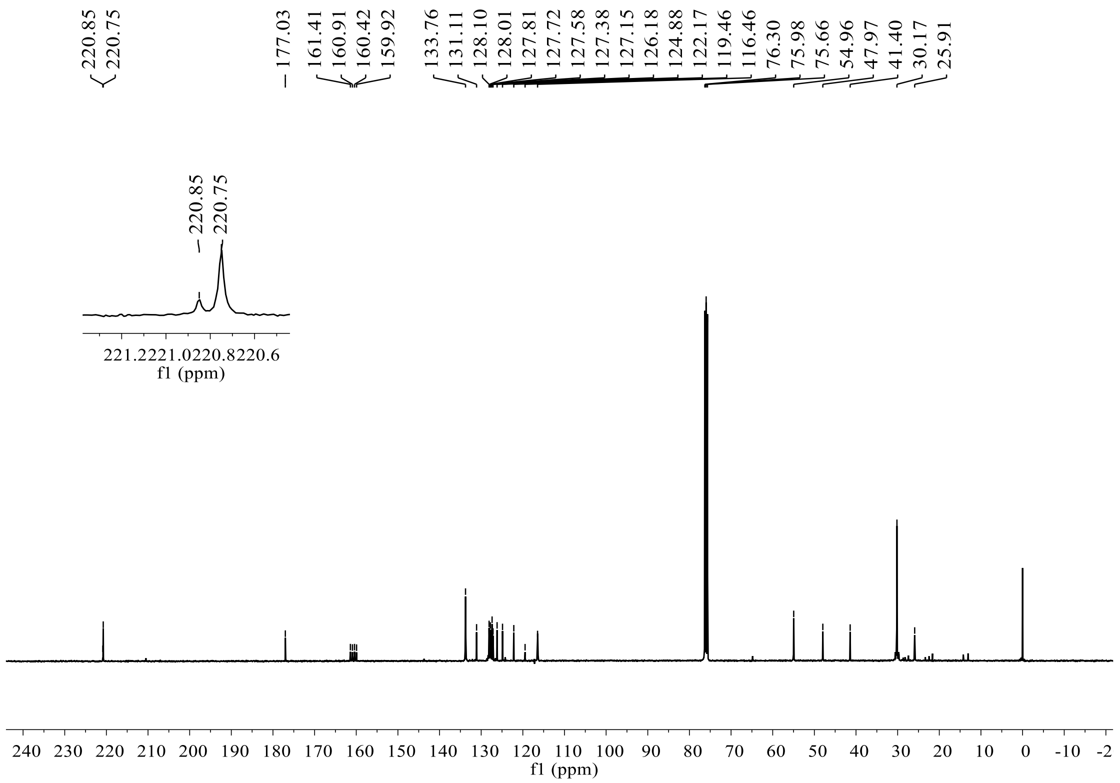

Supplementary Fig. 48. ^{13}C NMR spectrum of **8** in $CDCl_3$ at 298 K.

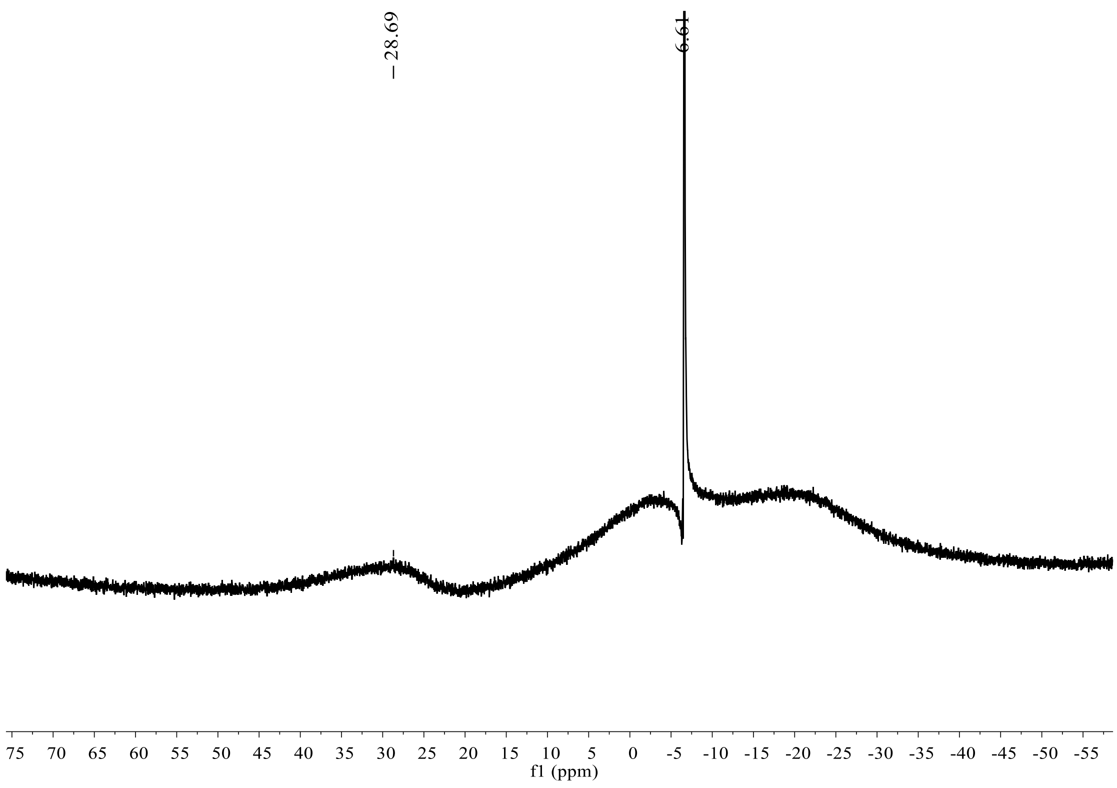

Supplementary Fig. 49. ^{11}B NMR spectrum of **8** in $CDCl_3$ at 298 K

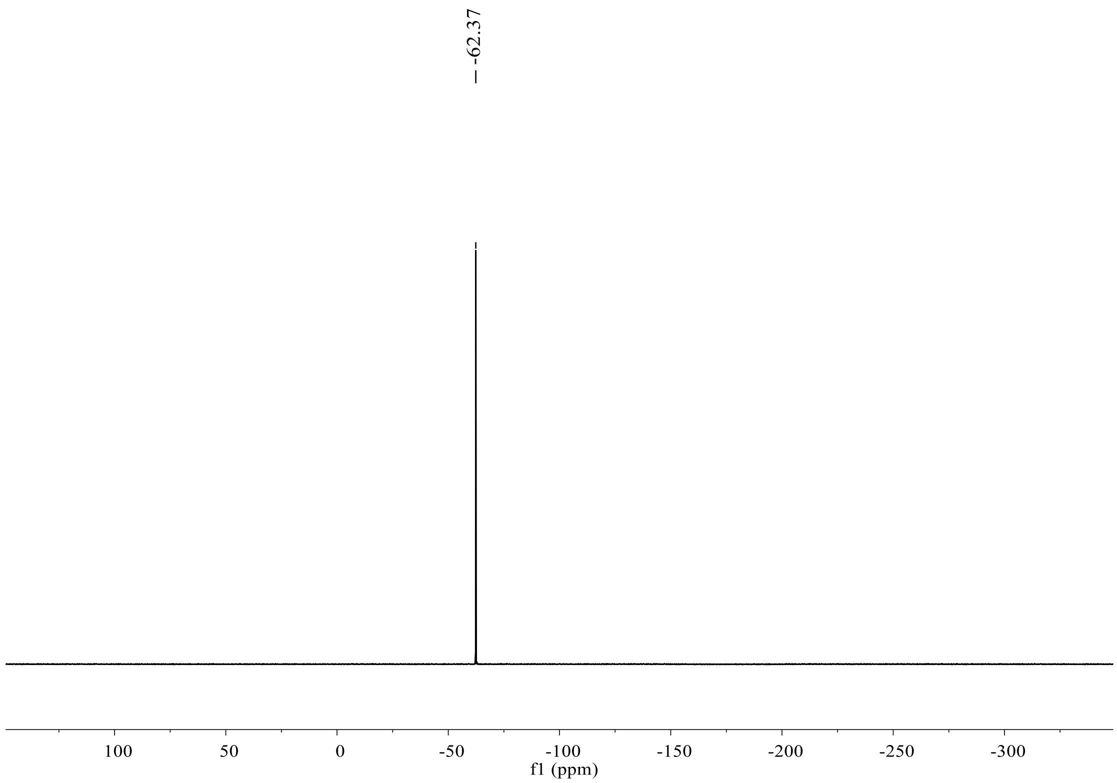

Supplementary Fig. 50. ^{19}F NMR spectrum of **8** in $CDCl_3$ at 298 K.

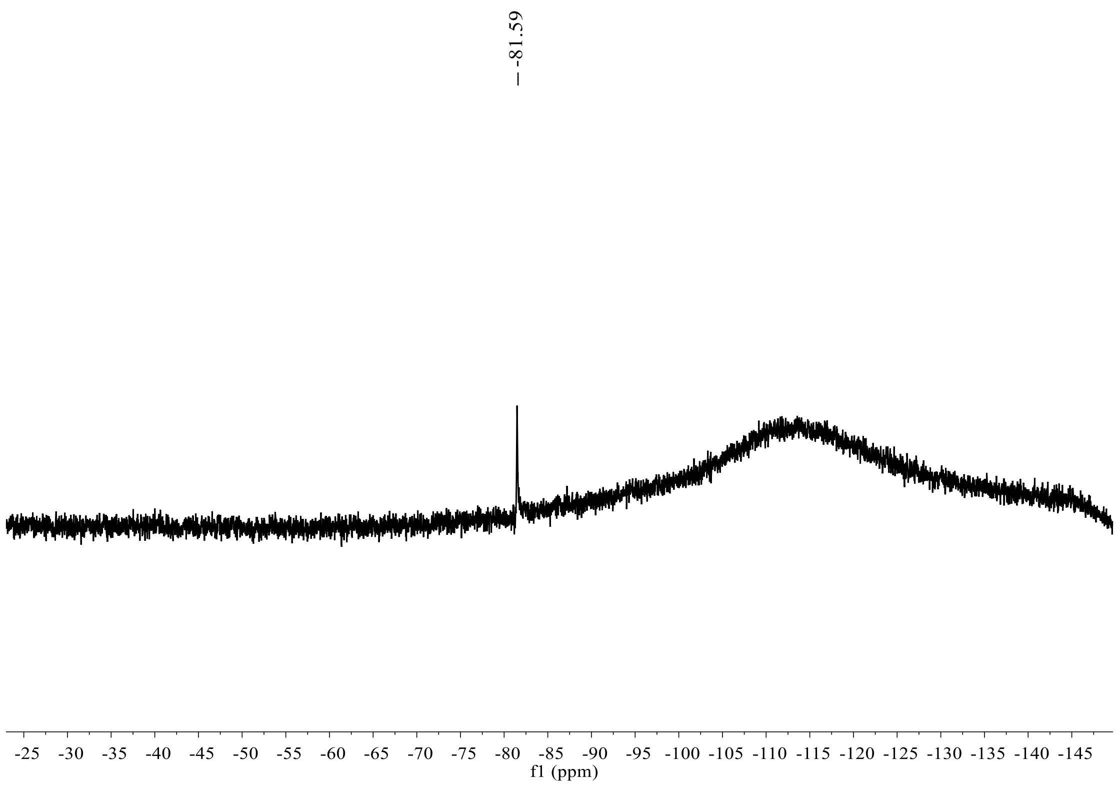

Supplementary Fig. 51. ^{29}Si NMR spectrum of **8** in $CDCl_3$ at 298 K.

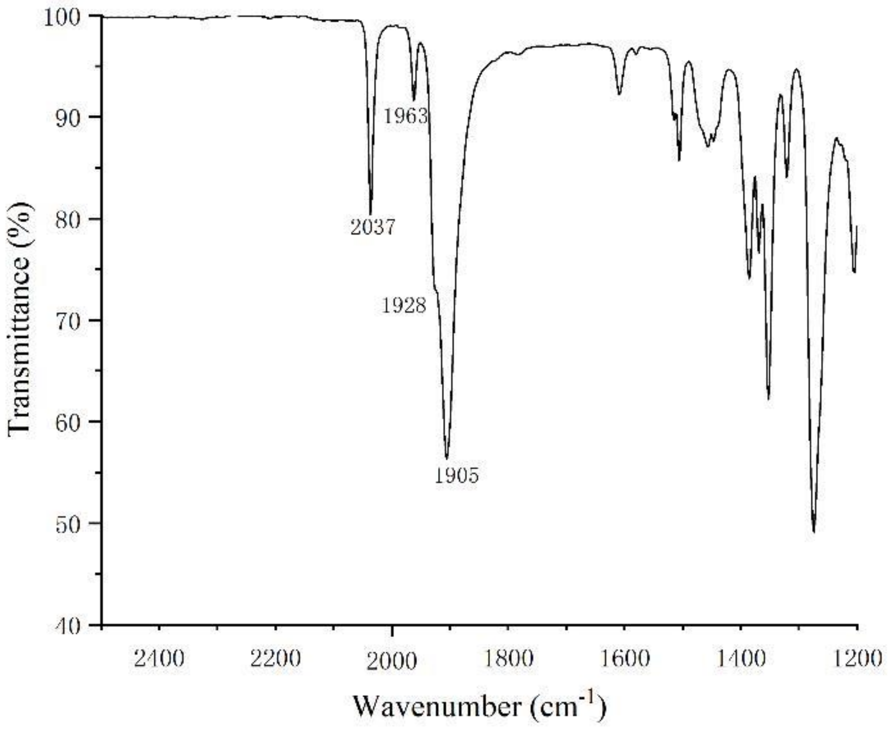

Supplementary Fig. 52 IR spectrum of **8**.

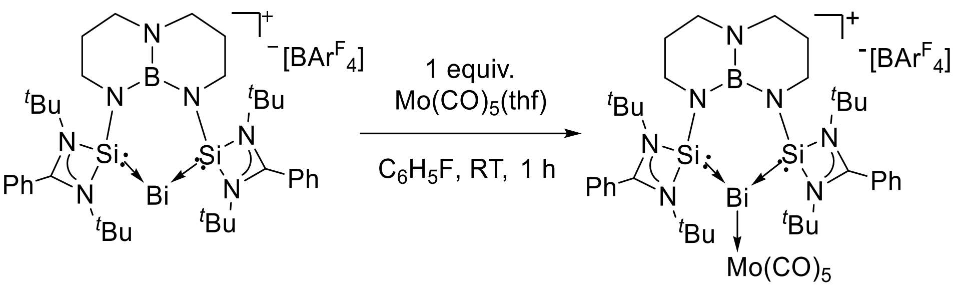

Supplementary Fig. 53. Synthesis of compound **9**.

Preparation of Compound 9: A sample of [Mo(CO)$_6$] (15 mg, 0.06 mmol) in THF (6 mL) was irradiated by UV-lamp for 1 h to generate yellow solution of [Mo(CO)$_5$(thf)], that was added to a solution of complex **3** (100 mg, 0.06 mmol) in fluorobenzene (5 mL) at room temperature The resulting yellow solution was stirred for a further 1 h at room temperature. The solvent was removed in vacuum, and the residue was washed with hexane (10 mL) and extracted with Et$_2$O (5 mL). The solvent was evaporated to yield compound **9** as a yellow powder (81 mg, 0.040 mmol, 70%). Single crystals suitable for X-ray diffraction studies were obtained by slow diffusion of hexane to the saturated fluorobenzene solutions at RT.

^{1}H NMR (400 MHz, CDCl$_3$, 298 K): δ 7.70 (s, 8H, Barf-Ar-*H*), 7.63-7.60 (m, 2H, Ar-*H*), 7.56-7.48 (m, 8H, Ar-*H*), 7.44-7.42 (m, 2H, Ar-*H*), 7.37-7.36 (m, 2H, Ar-*H*), 3.24 (br, 4H, NCH_2), 2.93 (br, 4H, NCH_2), 1.93 (br, 4H, CH$_2$-CH_2-CH$_2$), 1.26 (s, 36H, C(CH_3)$_3$)

^{13}C NMR (101 MHz, CDCl$_3$, 298 K): 208.5 (s, *C*O-ax.), 200.0 (s, *C*O-eq.) δ 176.7 (s, N*C*N), 160.7 (q, $J_{C\text{-}B}$= 51 Hz, Barf-Ar-*C*), 133.8 (s, Ar*C*), 131.1 (s, Ar*C*), 130.5 (s, Ar*C*), 128.3 (s, Ar*C*), 128.0 (m, Barf-Ar-*C*), 127.7 (m, Barf-Ar-*C*), 127.5 (s, Ar*C*), 127.4 (s, Ar*C*), 126.4 (s, Ar*C*), 126.0 (s, Ar*C*), 123.5 (q, $J_{C\text{-}F}$ = 274 Hz, Barf-*C*F$_3$), 116.4

(m, Barf-Ar-*C*), 54.5 (s, N*C*(CH_3)$_3$), 47.9 (s, N*C*H_2), 41.4 (s, N*C*H_2), 30.2. (s, C(*C*H_3)$_3$), 26.4 (s, CH_2-*C*H_2-CH_2).

^{11}B NMR (128 MHz, $CDCl_3$, 298 K): δ 29.8 ppm (br), -6.6 ppm (s, Barf-*B*).

^{19}F NMR (377 MHz, $CDCl_3$, 298 K): δ -62.4 ppm (s).

^{29}Si NMR (79 MHz, $CDCl_3$, 298 K): δ -78.6 ppm (s).

IR (CO, cm^{-1}): 2057, 1958, 1944, and 1913 cm^{-1}.

Anal. Calcd for$C_{73}H_{70}B_2MoF_{24}N_7O_5BiSi_2$: C, 44.64; H, 3.59; N, 4.99. Found: C, 45.32; H, 3.23; N, 4.73.

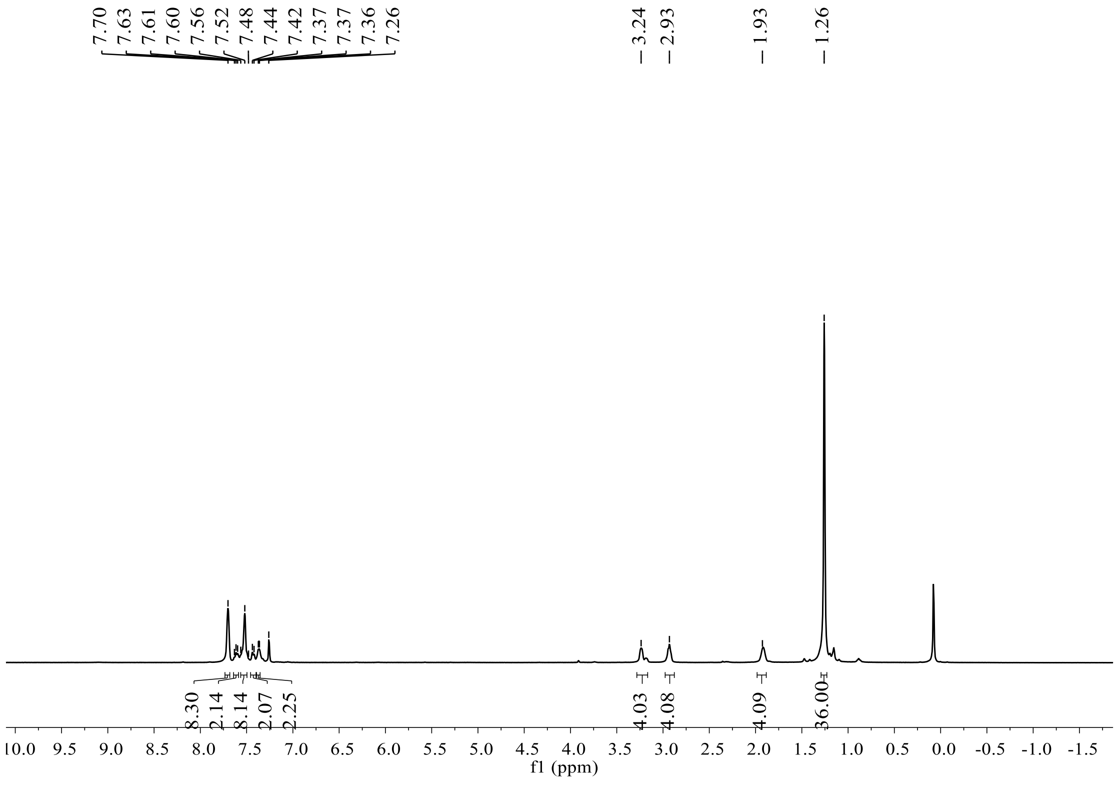

Supplementary Fig. 54. ^{1}H NMR spectrum of **9** in $CDCl_3$ at 298 K.

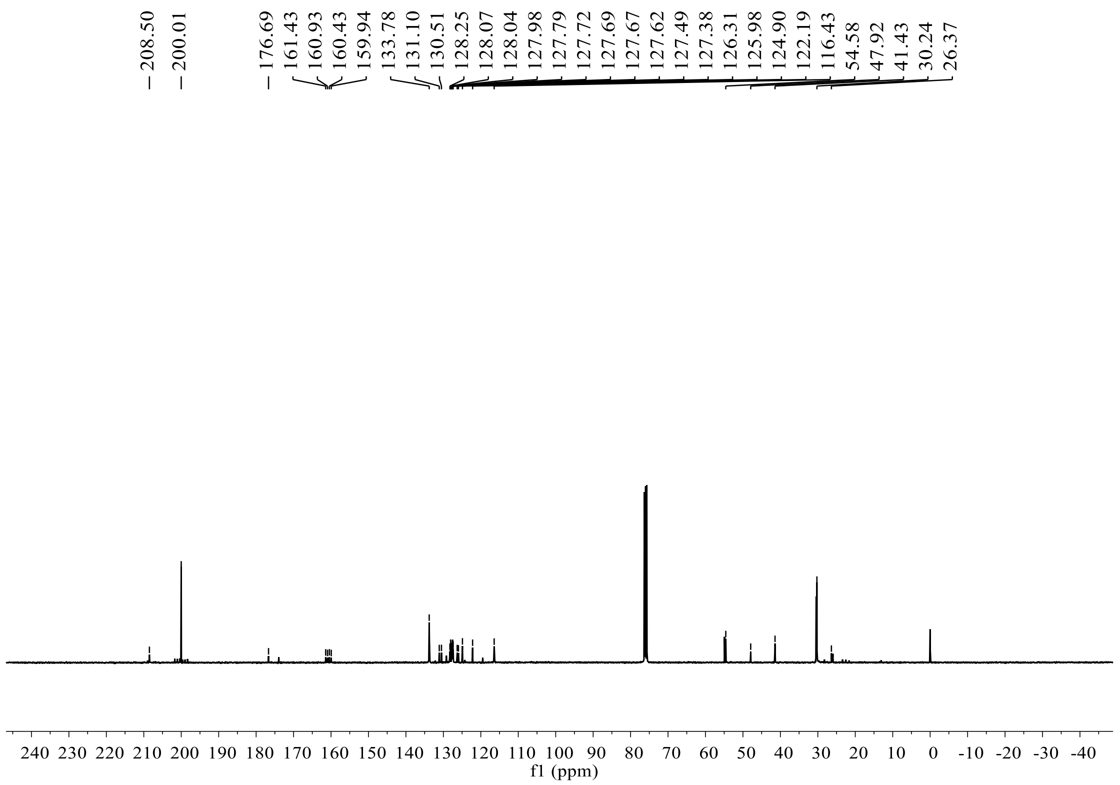

Supplementary Fig. 55. ^{13}C NMR spectrum of **9** in $CDCl_3$ at 298 K.

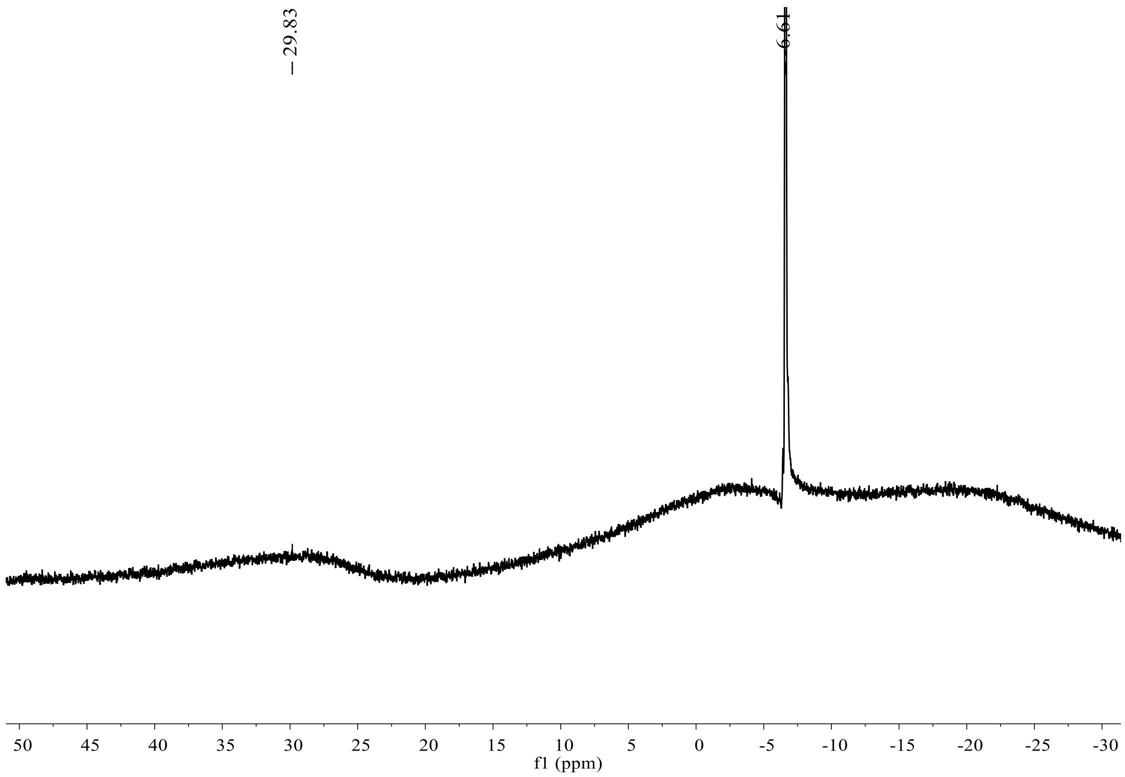

Supplementary Fig. 56. ^{11}B NMR spectrum of **9** in $CDCl_3$ at 298 K

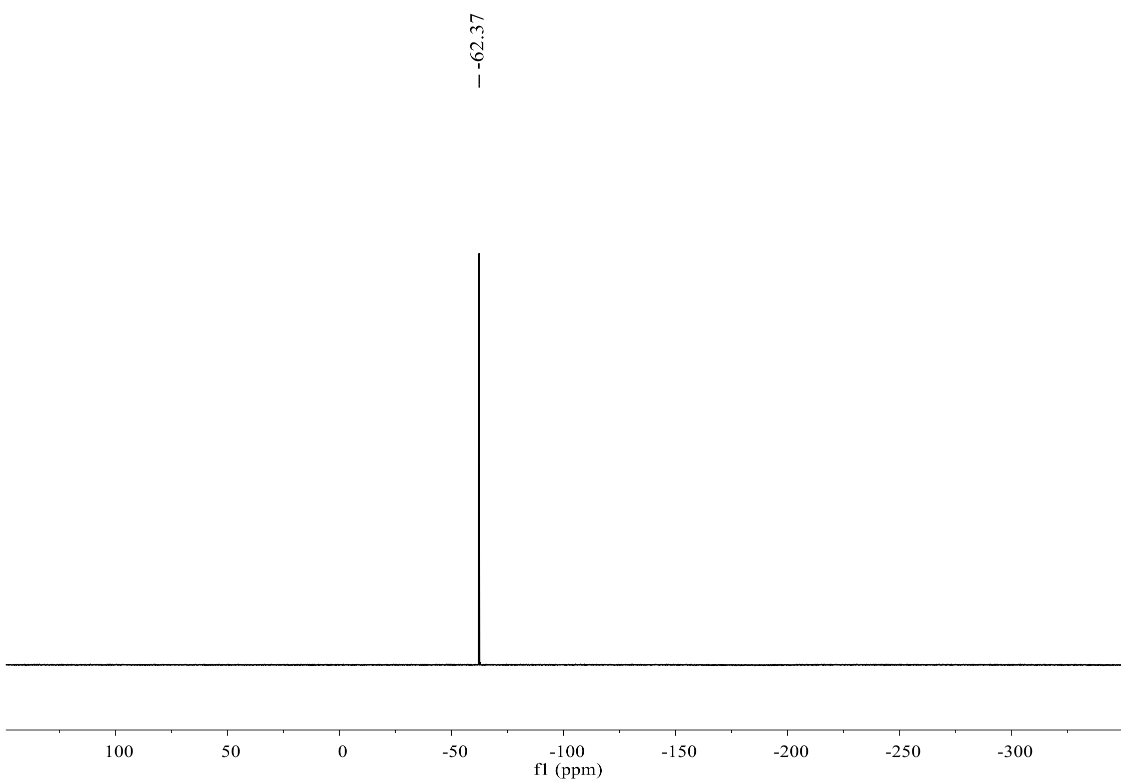

Supplementary Fig. 57. ^{19}F NMR spectrum of **9** in $CDCl_3$ at 298 K.

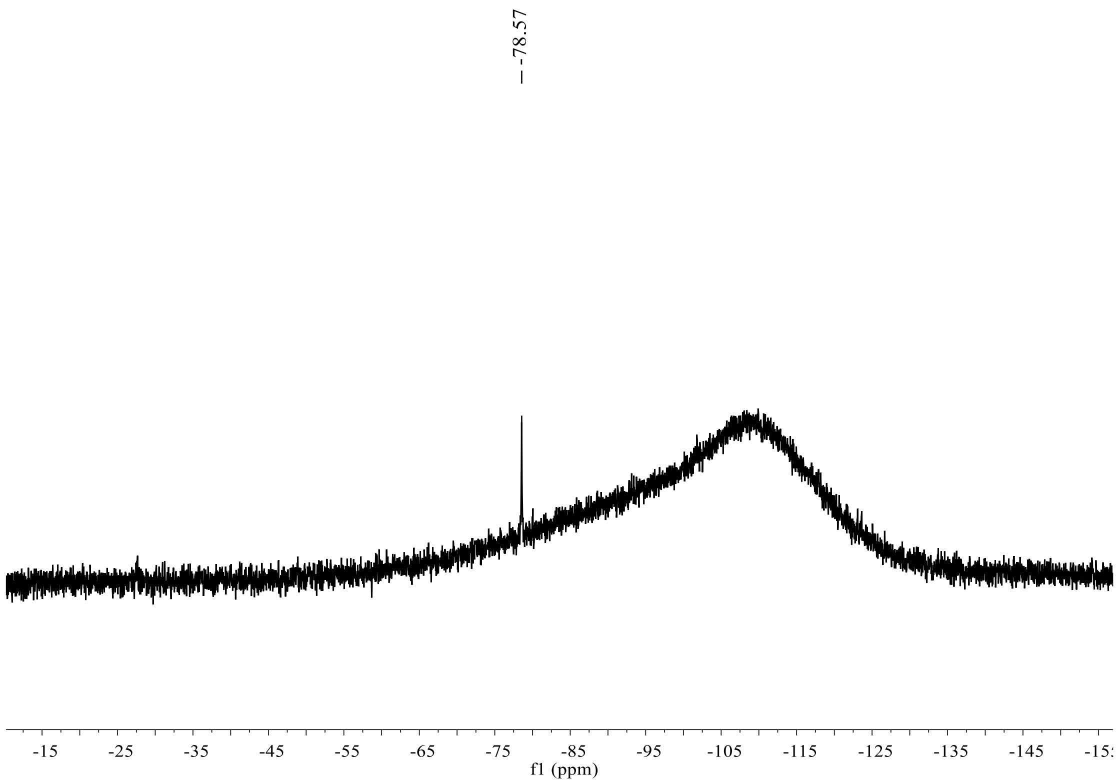

Supplementary Fig. 58. ^{29}Si NMR spectrum of **9** in $CDCl_3$ at 298 K.

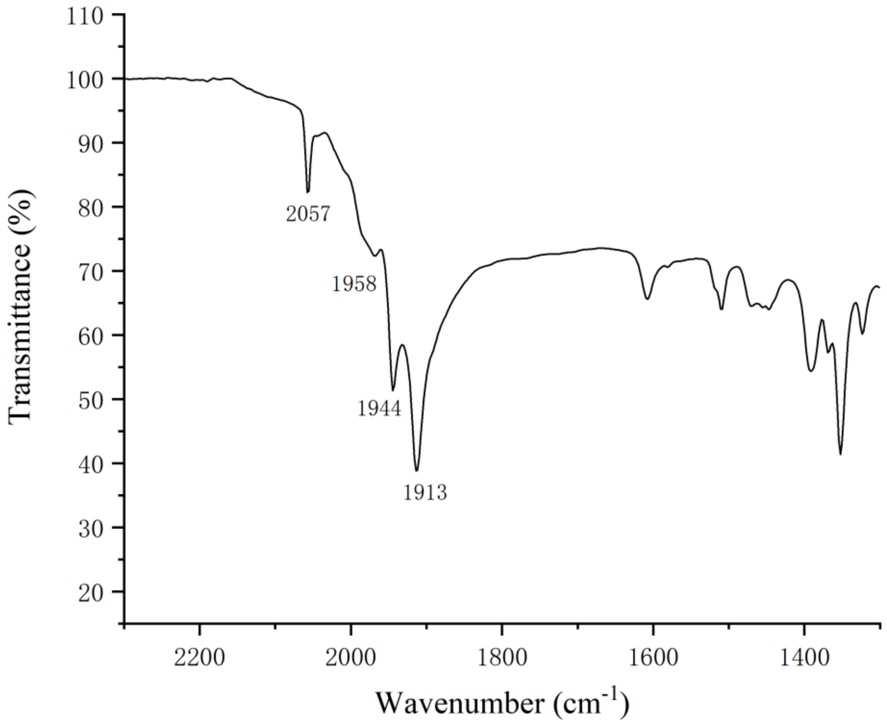

Supplementary Fig. 59 IR spectrum of **9**.

2. Supplementary Discussion

X-Ray Crystallography Data: Crystals for X-ray diffraction studies were obtained as described in the preparations. The crystals were manipulated in a glovebox under a microscope, and were sealed in thin-walled glass capillaries. Crystals for X-ray diffraction studies were obtained as described in the preparations. The X-ray crystallographic data for compounds **1-9** were collected with a Rigaku Saturn 724 CCD diffractometer using graphite-monochromated Mo Kα radiation (λ = 0.71073 Å) at 113(2) K. The structures were solved with the Olex2 and refined with the ShelXL refinement package using Least Squares minimization.[1,2] Refinement was performed on F^2 anisotropically for all the non-hydrogen atoms by the full-matrix least-squares method. The hydrogen atoms were placed at the calculated positions and were included in the structure calculation without further refinement of the parameters. The Olex2 program was utilized to draw the molecular structures.[1] Details of the crystallographic data and a summary of the intensity data collection parameters for **1-9** are listed in Table S1 and Table S2

For compound **5**:
Alert level B
PLAT090_ALERT_3_B Poor Data / Parameter Ratio (Zmax > 18) .... 5.54 Note
Response: A large number of parameters are increased due to large number of disordered groups in the structure, resulting in a decrease in the data/parameter ratio.

Refinement of 2: All the fluorine atoms of CF_3 groups on tetra(3,5-trifluoromethylphenyl)boron anion were refined as disorder on two sites with

occupancies of 0.437:0.563 (F1~F2:F1A~F3A), 0.681:0.319 (F4~F6:F4A~F6A), 0.256:0.744 (F7~F9:F7A~F9A), 0.167:0.833 (F10~F12:F10A~F12A), 0.822:0.178 (F13~F15:F13A~F15A), 0.604:0.396 (F16~F18:F16A~F18A), 0.876:0.124 (F19~F21:F19A~F21A), 0.682:0.318 (F22~F24:F22A~F24A), respectively. Some C-F bond lengths and F···F distances in same CF_3 group were restrained to be equal with an effective standard deviation 0.02 by applying the following SHELXL SADI instruction:

SADI 0.02 F5 C43 F5A C43

SADI 0.02 F4 C43 F4A C43

SADI 0.02 F6 C43 F6A C43

SADI 0.02 F14A F13A F15A F13A F15A F14A

SADI 0.02 F18A F16A F17A F16A F18A F17A

All the fluorine atoms closer than 3.8 Å were restrained with effective standard deviation 0.01 to have the same Uij components by applying the following SHELXL SIMU instruction:

SIMU 0.01 0.02 3.8 $F

Some fluorine atoms were restrained with effective standard deviation 0.005 so that their Uij components approximate to isotropic behavior by applying the following SHELXL ISOR instruction:

ISOR 0.005 0.01 F24A F24 F23A F23 F22A F22

The above SHELXL restraints were applied to make the refinement stable and the geometry of CF3 groups chemically reasonable.

Refinement of 3: All the fluorine atoms of CF_3 groups on tetra(3,5-trifluoromethylphenyl)boron anion were refined as disorder on two sites with occupancies of 0.118:0.882 (F1~F2:F1A~F3A), 0.681:0.319 (F4~F6:F4A~F6A), 0.675:0.326 (F7~F9:F7A~F9A), 0.967:0.033 (F10~F12:F10A~F12A), 0.728:0.272 (F13~F15:F13A~F15A), 0.491:0.509 (F16~F18:F16A~F18A), 0.876:0.124 (F19~F21:F19A~F21A), 0.305:0.695 (F22~F24:F22A~F24A), respectively. Some C-F bond lengths and F···F distances in same CF_3 group were restrained to be equal with an effective standard deviation 0.02 by applying the following SHELXL SADI instruction:

SADI 0.02 F8A F7A F9A F7A F9A F8A

All the fluorine atoms closer than 3.8 Å were restrained with effective standard deviation 0.01 to have the same Uij components by applying the following SHELXL SIMU instruction:

SIMU 0.01 0.02 3.8 $F

Some fluorine atoms were restrained with effective standard deviation 0.003 so that their Uij components approximate to isotropic behavior by applying the following SHELXL ISOR instruction:

ISOR 0.003 0.006 F13A F15 F14 F15A F13 F14A F19A F19 F21A F20 F20A F21

The above SHELXL restraints were applied to make the refinement stable and the geometry of CF_3 groups chemically reasonable.

Refinement of 5: Pseudomerohedral twin was detected by PLATON producing the twin law of -1 0 0 0 -1 0 0 0 -1 2 and batch scale factor of 0.121, which were included

in the refinement as following:

BASF 0.121

TWIN -1 0 0 0 -1 0 0 0 -1 2

The CF_3 groups of tetra(3,5-trifluoromethylphenyl)boron anion were disordered and refined over two sites with occupancies of 0.418:0.581 (C44, F2~F5:C44A, F2A~F4A), 0.648:0.352 (C52, F8~F10:C52A, F8A~F10A), 0.613:0.387 (C68, F20~F22:C68A, F20A~F22A), 0.616:0.384 (F26~F28:F26A~F28A), 0.469:0.531 (C77, F29~F31:C77A, F29A~F31A), 0.698:0.302 (C85, F35~F37:C85A, F35A~F37A), 0.604:0.396 (C92, F38~F40:C92A, F38A~F40A), 0.307:0.693 (C93, F41~F43:C93A, F41A~F43A), 0.590:0.410 (C100, F44~F46:C114, F44A~F4A6), 0.559:0.441 (C101, F47~F49:C115, F47A~F49A), respectively. The 3,5-trifluoromethylphenyl group of tetra(3,5-trifluoromethylphenyl)boron anion was disordered and refined over two sites with occupancy of 0.624:0.376 (C56~C61, F14~F19:C56A~C61A, F14A~F19A). The fluorobenzene solvent was disordered and refined over two sites with occupancy of 0.763:0.237 (F1, C102~C107:F1A, C108~C113). The Bi-Me moiety was disordered and refined over two sites with occupancy of 0.779:0.221 (Bi1-C1:Bi1A-C1A). The tBu groups were disordered and refined over two sites with occupancies of 0.340:0.660 (C15~C17:C15A~C17A), 0.900:0.100 (C35~C37:C35A~C37A), respectively. The phenyl group was disordered and refined over two sites with occupancy of 0.592:0.408 (C24~C29:C24A~C29A). In addition to the above treatment, much more restraints such as DFIX, FLAT, ISOR, RIGU, SADI and SIMU (see .ins file embedded in .cif file) were added to stabilize the refinement.

Refinement of 6: The CF_3 groups of tetra(3,5-trifluoromethylphenyl)boron anion were disordered and refined over two sites with occupancies of 0.511:0.489 (F10~F12:F10A~F12A), 0.652:0.348 (F13~F15:F13A~F15A), 0.659:0.341 (F22~F23:F22A~F23A), respectively. The Solvent Mask routine[Acta Cryst, 2005, D61, 1299−1301.] of the OLEX2 software[J Appl Cryst, 2009, 42, 339−341.] was implemented to remove the contributions of about two highly disordered n-hexanes, which couldn't be modelled, to the observed structure factors. In addition to the above treatment, much more restraints such as DELU, ISOR and SADI (see .ins file embedded in .cif file) were added to stabilize the refinement.

Refinement of 7: The entire tetra(3,5-trifluoromethylphenyl)boron anion were disordered and refined over two sites with occupancy of 0.654:0.346. The ^{t}Bu groups were disordered and refined over two sites with occupancies of 0.676:0.324 (C12~C15:C12A~C15A), 0.586:0.414 (C27~C30:C27A~C30A), respectively. The BN3 motif was disordered and refined over two sites with occupancy of 0.509:0.491 (B1, N1~N3, C6~C11:B1A, N1A~N3A, C6A~C11A). In addition to the above treatment, much more restraints such as DFIX, FLAT, ISOR, RIGU, SADI and SIMU (see .ins file embedded in .cif file) were added to stabilize the refinement.

Refinement of 8: The CF_3 groups of tetra(3,5-trifluoromethylphenyl)boron anion were disordered and refined over two sites with occupancies of 0.327:0.673 (F4~F6:F4A~F6A), 0.540:0.460 (F7~F9:F7A~F9A), 0.820:0.180 (F10~F12:F10A~F12A), 0.482:0.518 (F16~F18:F16A~F18A), 0.789:0.211 (F22~F24:F22A~F24A), 0.471:0.529 (F28~F30:F28A~F30A), 0.720:0.280

(F34~F36:F34A~F36A), 0.672:0.328 (F40~F42:F40A~F42A), respectively. In addition to the above treatment, much more restraints such as DELU, ISOR, SADI and SIMU (see .ins file embedded in .cif file) were added to stabilize the refinement.

Refinement of 9: The CF_3 groups of tetra(3,5-trifluoromethylphenyl)boron anion were disordered and refined over two sites with occupancies of 0.724:0.276 (F7~F9:F7A~F9A), 0.220:0.780 (C10C, F28~F30:C40DF28A~F30A), 0.645:0.355 (C56C, F40~F42:C56D, F40A~F42A), 0.783:0.213 (C64C, F46~F48:C64D, F46A~F48A), respectively. The 3,5-trifluoromethylphenyl group of tetra(3,5-trifluoromethylphenyl)boron anion was disordered and refined over two sites with occupancy of 0.591:0.409 (C2C~C8C, F1~F6:C2D~C8D, F1A~F6A).

Supplementary Tab. 1. X-Ray crystallographic data and structure refinement for 1-5.

	**1**	**2**	**3**
formula	$C_{36}H_{58}BN_7Si_2$	$C_{68}H_{70}B_2F_{24}N_7SbSi_2$	$C_{68}H_{70}B_2BiF_{24}N_7Si_2$
formula weight	655.88	1640.86	1728.09
crystal system	orthorhombic	triclinic	triclinic
space group	*P*nn2	*P*-1	*P*-1
a, Å	10.6104(6)	11.3550(3)	11.3426(3)
b, Å	15.4269(8)	19.2642(5)	19.2957(5)
c, Å	14.3584(10)	19.4635(6)	19.4802(5)
α, deg	90	118.460(3)	118.513(3)
β, deg	90	99.007(2)	98.951(2)
γ, deg	90	90.209(2)	90.351(2)
V, Å^3	2350.3(2)	3681.8(2)	3684.84(19)
Z	2	2	2
D_{calcd}, g/cm^3	0.927	1.480	1.557
temp, K	273(2)	273(2)	273(2)
μ, mm-1	0.103	0.513	2.531
reflections collected	21468	38823	39541
independent reflections (R_{int})	4139 (0.0579)	15040 (0.0316)	15051(0.0533)
$R1$ ($I > 2\sigma(I)$)	0.0748	0.0545	0.0416
$wR2$ ($I > 2\sigma(I)$)	0.2091	0.1365	0.1049
$wR2$ (all data)	0.2217	0.1421	0.1084
parameters	204	1061	1061
GOF	1.055	1.048	1.028

Supplementary Tab. 2. X-Ray crystallographic data and structure refinement for 6-9.

	**5**	**6**	**7**
formula	$C_{107}H_{90}B_3BiF_{49}N_7Si_2$	$C_{73}H_{70}B_2CrF_{24}N_7O_5SbSi_2$	$C_{73}H_{70}B_2F_{24}MoN_7O_5SbSi_2$
formula weight	2702.44	1832.91	1876.85
crystal system	monoclinic	monoclinic	monoclinic
space group	la	$P2_1/c$	$P2_1/c$
a, Å	26.1234(12)	12.4295(2)	21.5585(4)
b, Å	18.3135(5)	36.5769(5)	18.2567(4)
c, Å	27.0796(13)	21.1857(3)	19.4635(4)
α, deg	90	90	90
β, deg	115.744(6)	106.359(2)	111.832(2)
γ, deg	90	90	90
V, Å^3	11669.3(10)	9241.8 (3)	8189.0(3)
Z	4	4	4
D_{calcd}, g/cm^3	1.538	1.317	1.522
temp, K	273(2)	113(2)	113(2)
μ, mm-1	1.659	0.530	0.619
reflections collected	50909	69898	118545
independent reflections (R_{int})	22873 (0.0711)	16192 (0.0411)	16670 (0.0908)
$R1$ ($I > 2\sigma(I)$)	0.0653	0.0529	0.0538
$wR2$ ($I > 2\sigma(I)$)	0.1333	0.1357	0.1152
$wR2$ (all data)	0.1518	0.1417	0.1277
parameters	2146	1132	1637
GOF	1.023	1.049	1.028

Supplementary Tab. 3. X-Ray crystallographic data and structure refinement for 6-9.

	**8**	**9**
formula	$C_{73}H_{70}B_2CrF_{24}N_7O_5BiSi_2$	$C_{73}H_{70}B_2BiF_{24}MoN_7O_5Si_2$
formula weight	1956.69	2001.13
crystal system	triclinic	triclinic
space group	*P*-1	*P*-1
a, Å	19.1320(2)	19.3515(2)
b, Å	22.2314(2)	22.0255(3)
c, Å	122.5795(2)	22.3183(2)
α, deg	73.9260(10)	75.3980(10)
β, deg	88.8330(10)	89.9930(10)
γ, deg	67.8140(10)	69.2230(10)
V, Å^3	8506.74.(16)	8565.33(18)
Z	2	2
D_{calcd}, g/cm^3	1.528	1.552
temp, K	113(2)	113(2)
μ, mm-1	2.326	2.330
reflections collected	108909	146668
independent reflections (R_{int})	29669(0.0716)	34366 (0.0517)
$R1$ ($I > 2\sigma(I)$)	0.0407	0.0610
$wR2$ ($I > 2\sigma(I)$)	0.0985	0.1295
$wR2$ (all data)	0.1033	0.1357
parameters	2385	2485
GOF	1.034	1.114

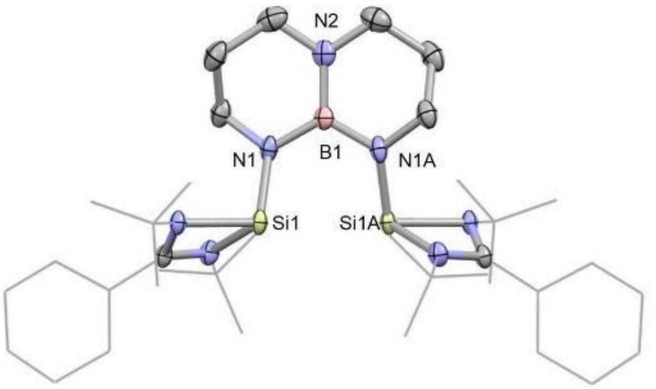

Supplementary Fig. 60. X-ray crystal structure of **1** at 30% probability ellipsoids. Hydrogen atoms are omitted for clarity. Selected bond distances (Å) and angles (º): Si1···Si1A 3.005(2); Si1−N1 1.761(5), B1−N1 1.452(6), N1−B1−N1A 121.4.

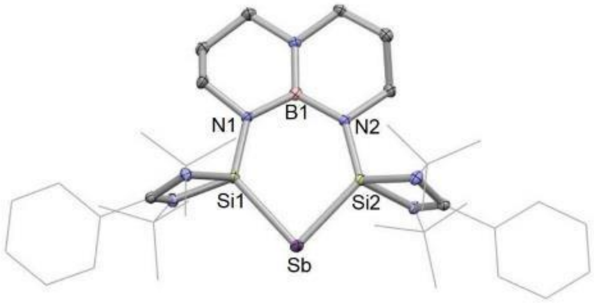

Supplementary Fig. 61. X-ray crystal structure of **2** at 30% probability ellipsoids. The anion [BAr^F$_4$] and hydrogen atoms are omitted for clarity. Selected bond lengths (Å) and angles (º): Sb1−Si1 2.4619(9), Sb1−Si2 2.455(5), Si1−N1 1.703(3), Si2−N2 1.706(2), Si1−Sb1−Si2 85.33(3), N1−B1−N2 123.1(2).

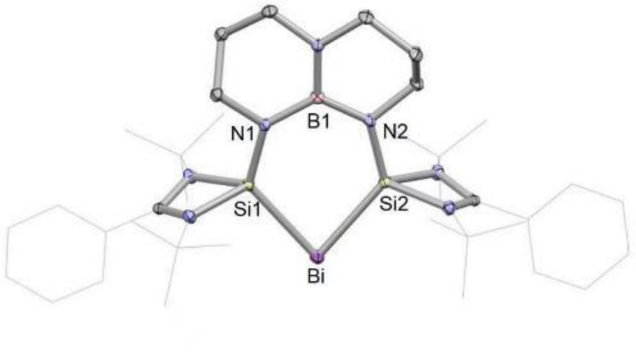

Supplementary Fig. 62. X-ray crystal structure of **3** at 30% probability ellipsoids. The anion [$BAr^F{}_4$] and hydrogen atoms are omitted for clarity. Selected bond lengths (Å) and angles (º): Bi1−Si1 2.561(8), Bi1−Si2 2.557(1), Si1−N1 1.710(3), Si2−N2 1.712(2), Si1−Bi1-Si2 82.10(3), N1−B−N2 123.5(3).

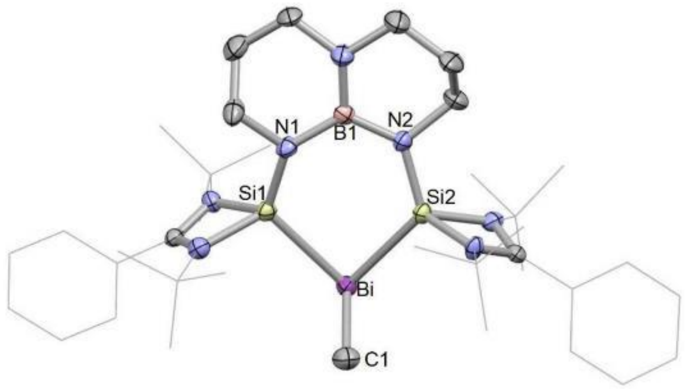

Supplementary Fig. 63. X-ray crystal structure of **5** at 30% probability ellipsoids. The anion [$BAr^F{}_4$] and hydrogen atoms are omitted for clarity. Selected bond lengths (Å) and angles (º): Bi1−Si1 2.651, Bi1−Si2 2.636, Bi1−C1 2.300, Si1−N1 1.681(8), Si2−N2 1.69(1), Si1−Bi1−Si2 81.70, Si1−Bi1−C1 98.76, Si2−Bi1−C1 95.55, N1−B1−N2 124(1).

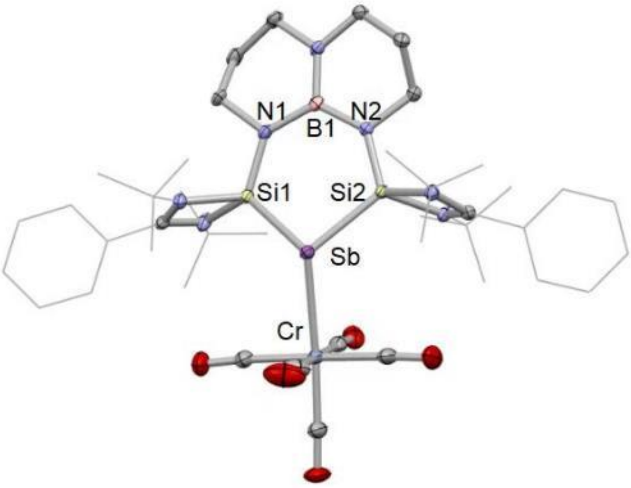

Supplementary Fig. 64. X-ray crystal structure of **6** at 30% probability ellipsoids. The anion [BAr$^F{}_4$] and hydrogen atoms are omitted for clarity. Selected bond lengths (Å) and angles (°):Sb1−Si1 2.495(9), Sb1−Si2 2.498(1), Sb1−Cr1 2.702(1), Si1−N1 1.70(3), Si2−N2 1.701(3), Si1−Sb1−Si2 88.02(3)°, Si1−Sb1−Cr1 118.65(3)°, Si2−Sb1−Cr1 118.80(3)°, N1−B1−N2 124.0(3)°.

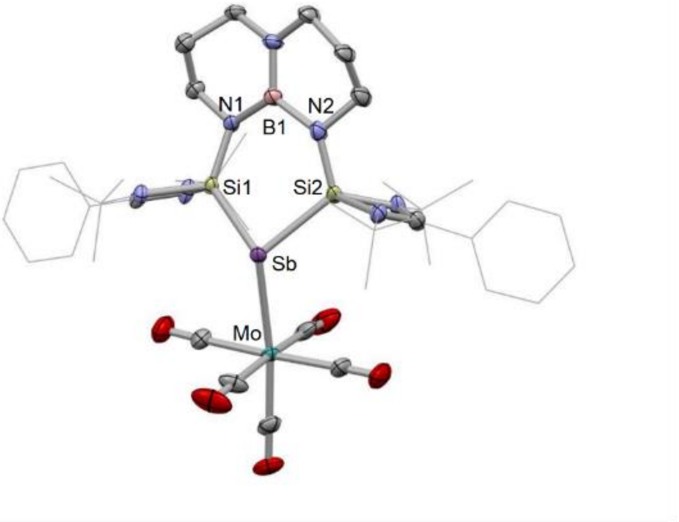

Supplementary Fig. 65. X-ray crystal structure of **7** at 30% probability ellipsoids. The anion [BAr$^F{}_4$] and hydrogen atoms are omitted for clarity. Selected bond lengths (Å) and angles (°): Sb1−Si1 2.485(1), Sb1−Si2 2.498(1), Sb1−Mo1 2.8584(6), Si1−N1 1.68(3), Si2−N2 1.67(2), Si1−Sb1−Si2 87.76(4)°, Si1−Sb1−Mo1 120.16(3)°, Si2−Sb1−Mo1 117.53(3)°, N1−B1−N2 116(1)°.

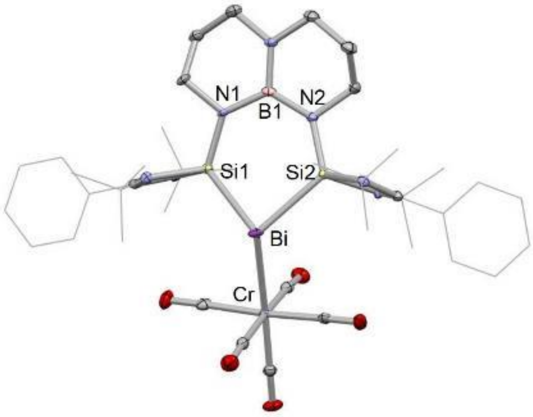

Supplementary Fig. 66. X-ray crystal structure of **8** at 30% probability ellipsoids. The anion $[BAr^F{}_4]$ and hydrogen atoms are omitted for clarity. Selected bond lengths (Å) and angles (º): Bi1−Si1 2.609(3), Bi1−Si2 2.588(4), Bi1−Cr1 2.802(3), Si1−N1 1.698(4), Si2−N2 1.709(3), Si1−Bi1−Si2 84.0(1)°, Si1−Bi1−Cr1 114.6(1)°, Si2−Bi1−Cr1 115.9(1)°, N1−B1−N2 123.9(3)°.

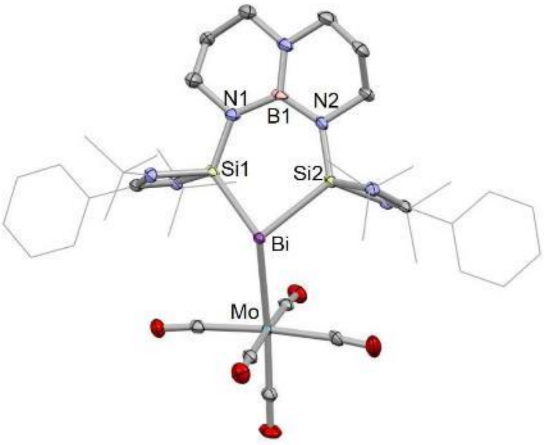

Supplementary Fig. 67. X-ray crystal structure of **9** at 30% probability ellipsoids. The anion $[BAr^F{}_4]$ and hydrogen atoms are omitted for clarity. Selected bond lengths (Å) and angles (º): Bi1−Si1 2.600(2), Bi1−Si2 2.599(1), Bi1−Mo1 2.9450(7), Si1−N1 1.707(5), Si2−N2 1.711(5), Si1−Bi1−Si2 83.69(6)°, Si1−Bi1−Mo1 114.69(3)°, Si2−Bi1−Mo1 116.90(4)°, N1−B1−N2 124.4(7)°.

Computational Studies: General details: All DFT calculations were carried out with Gaussian16-C.01 quantum chemical package.[3] Geometry optimizations were performed in the gas phase with the BP86+D3(BJ)/def2-TZVPP level of theory. Vibrational frequency calculations were carried out at BP86+D3(BJ)/def2-SVP level of theory (No imaginary frequency for local minima) and to provide the thermal corrections for Gibbs free energy determinations at 298.15 K and 1 atm. The UV-vis spectra of **2** and **3** were calculated using TD- PBE1PBE method at def2SVP basis set. Natural Bond Orbital (NBO) analyses were performed using the NBO 7.0 program at BP86+D3(BJ)/def2-TZVPP level.[4] Wiberg bond indexes were determined from the Natural Atomic Orbital basis. The multiwfn[5,6] program was used for ETS-NOCV analyses.[7] Graphical structures are visualized with VMD[8] and CYLview[9].

The bonding situation was analyzed by means of an energy decomposition analysis (EDA)[10,11] together with the natural orbitals for chemical valence (NOCV)[12,13] method by using the ADF 2019.103 program package[14,15]. The EDA-NOCV calculations were carried out at the BP86-D3(BJ)/TZ2P-Zora level[16,17] by using the optimized geometries obtained at the BP86/def2-TZVPP level. In this analysis, the intrinsic interaction energy (ΔE_{int}) between two fragments can be divided into four energy components as follows:

$$\Delta E_{int} = \Delta E_{elstat} + \Delta E_{Pauli} + \Delta E_{orb} + \Delta E_{disp} \qquad (1)$$

While the electrostatic ΔE_{elstat} term represents the quasiclassical electrostatic interaction between the unperturbed charge distributions of the prepared fragments, the Pauli repulsion ΔE_{Pauli} corresponds to the energy change associated with the transformation from the superposition of the unperturbed electron densities of the isolated fragments to the wavefunction[18], which properly obeys the Pauli principle through explicit antisymmetrization and renormalization of the production wavefunction.

The orbital term ΔEorb can be further decomposed into contributions from each irreducible representation of the point group of the interacting system as follows:

$$\Delta E_{orb} = \sum_{r} \Delta E_{r} \qquad (2)$$

The addition of ΔE_{prep} to the intrinsic interaction energy ΔEint gives the total energy ΔE, which has opposite sign compared with the bond dissociation energy De [Eq. (3)].

$$\Delta E(-D_e) = \Delta E_{int} + \Delta E_{prep} \qquad (3)$$

The combination of the EDA with NOCV enables the partition of the total orbital interactions into pairwise contributions of the orbital interactions which is very vital to get a complete picture of the bonding. The charge deformation $\Delta\rho_{k(r)}$, resulting from the mixing of the orbital pairs $\psi_{k(r)}$ and $\psi_{-k(r)}$ of the interacting fragments presents the amount and the shape of the charge flow due to the orbital interactions [Eq. (4)], and the associated energy term ΔEorb provides with the size of stabilizing orbital energy originated from such interaction [Eq. (5)].

$$\Delta\rho_{orb}(r) = \sum_{k} \Delta\rho_{k}(r) = \nu_{k}\,[-\psi^{2}_{-k}(r) + \psi^{2}_{k}(r)] \qquad (4)$$

$$\Delta E_{orb} = \sum_{k} \Delta E^{k}_{orb} = \sum_{k=1}^{N/2} \nu_{k}[F^{TS}_{-k,-k} + F^{TS}_{k,k}] \qquad (5)$$

More details about the EDA-NOCV method and its application are given in recent reviews articles[19-22].

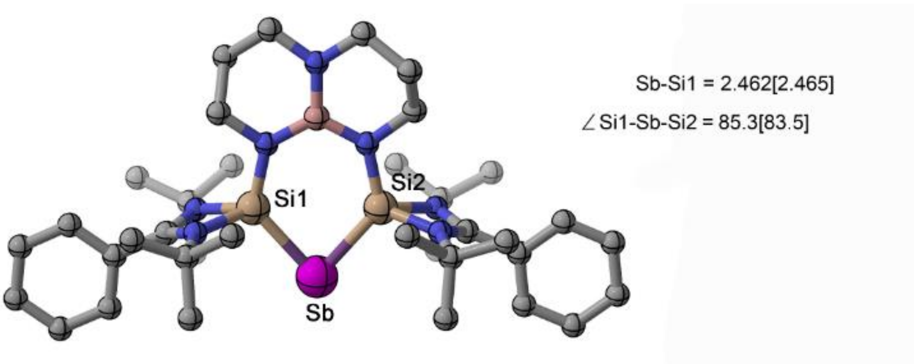

Singlet: ΔE = 0.0 kcal mol^{-1}
Triplet: ΔE = 47.0 kcal mol^{-1}

2

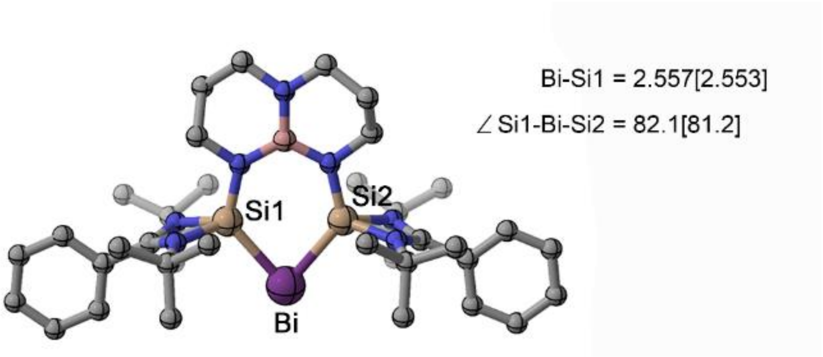

Singlet: ΔE = 0.0 kcal mol^{-1}
Triplet: ΔE = 36.8 kcal mol^{-1}

3

Supplementary Fig. 68. Optimized geometry of the complex **2** and **3** at the BP86+(D3BJ)/def2-TZVPP level. Selected bond lengths in Å, the experimental bond are compared in parentheses. Hydrogen atoms are omittes clarity(color code, Sb: hot pink, Bi: purple, Si: khaki, C: gray, N:blue, B: pink)

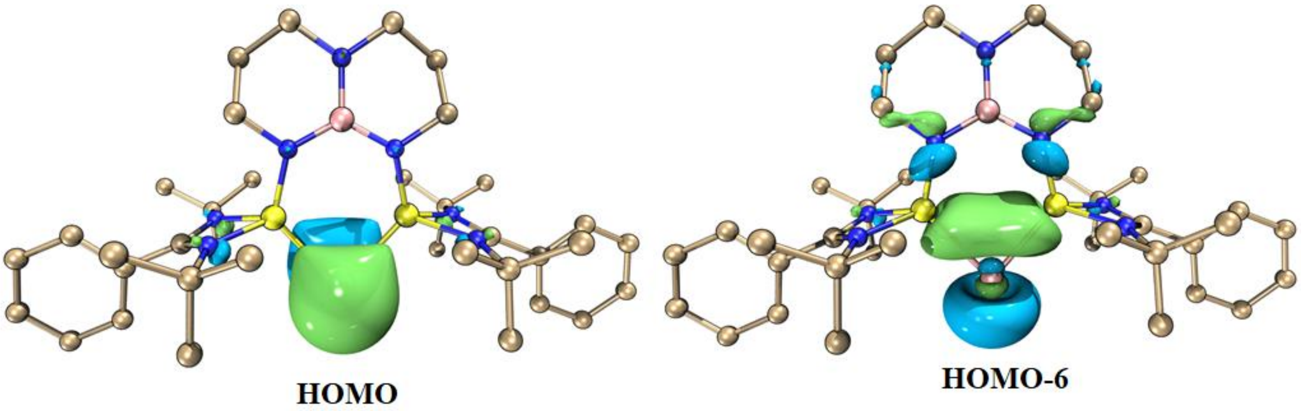

Supplementary Fig. 69. Selected Kohn-Sham isosurfaces (0.05 au) showing HOMO and HOMO–6 in **3**.

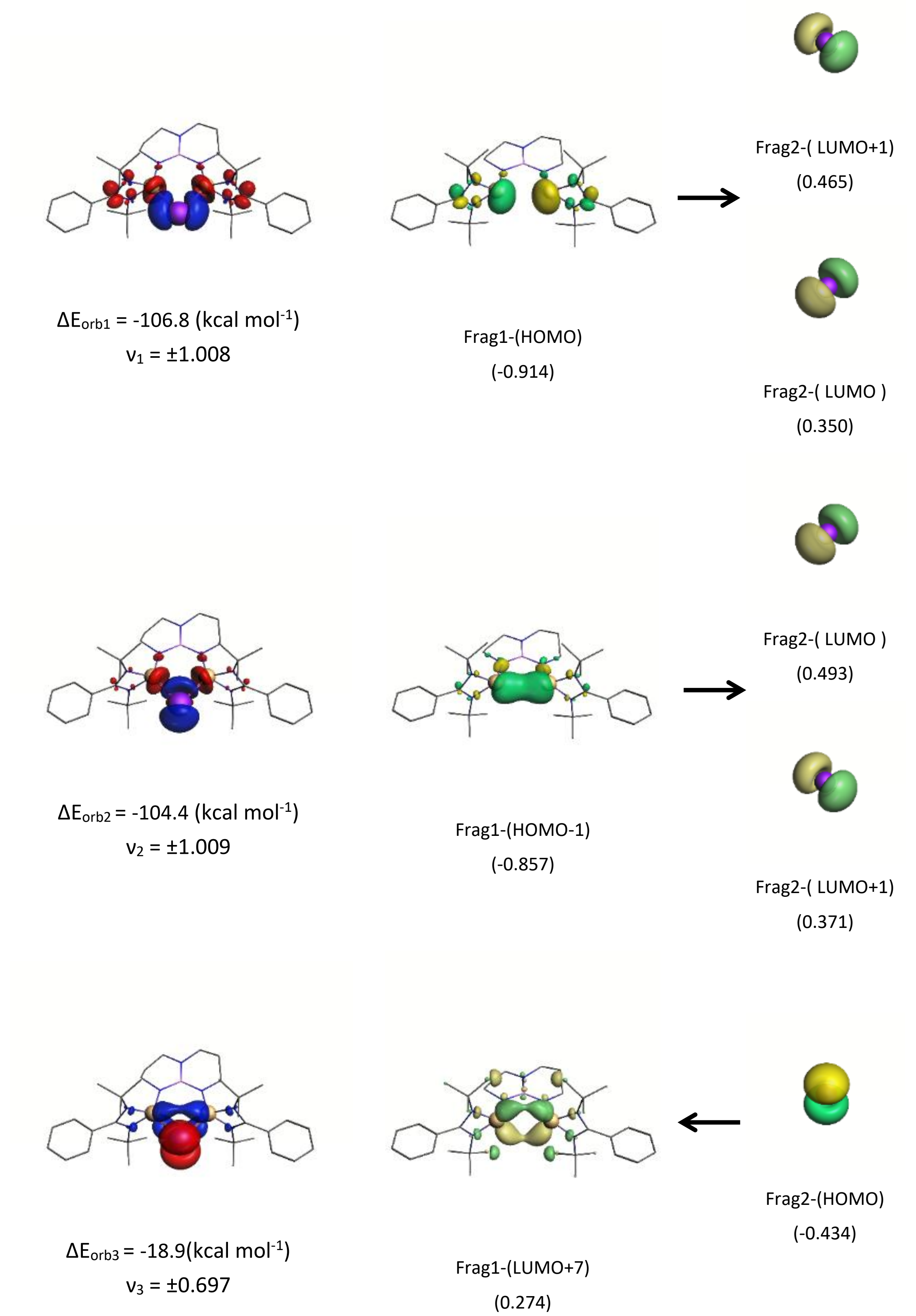

Supplementary Fig. 70. Plot of deformation densities Δρ of the pairwise orbital interactions and the associated interaction energies (ΔE$_{orb}$) between fragments, as well as the shape of the most important interacting MOs of the two fragments [(TBDSi$_2$)Pn][BArF$_4$] and Bi^+ in **3**. The direction of the charge

flow is red to blue.

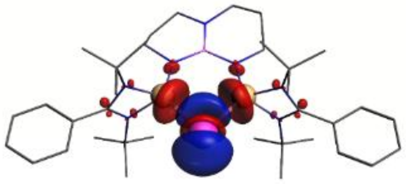

ΔE_{orb1} = -125.4 (kcal mol^{-1})
ν_1 = ±1.085

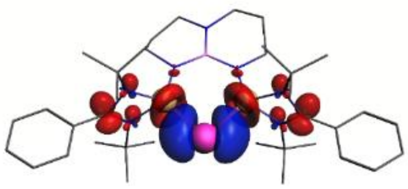

ΔE_{orb2} = -121.4 (kcal mol^{-1})
ν_2 = ±1.031

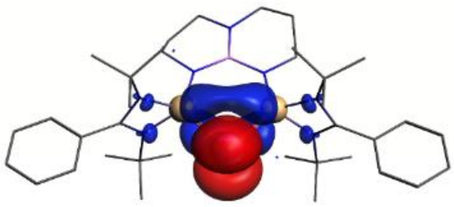

ΔE_{orb3} = -20.7(kcal mol^{-1})
ν_3 = ±0.714

Supplementary Fig. 71. Plot of deformation densities Δρ of the pairwise orbital interactions between the two fragments of [(TBDSi$_2$)Pn][BArF$_4$] and Sb^+ in **2** together with the associated interaction energies ΔE_{orb} (in kcal mol^{-1}). The eigenvalues ν are a measure for the relative amount of charge transfer. The direction of the charge flow is from red to blue.

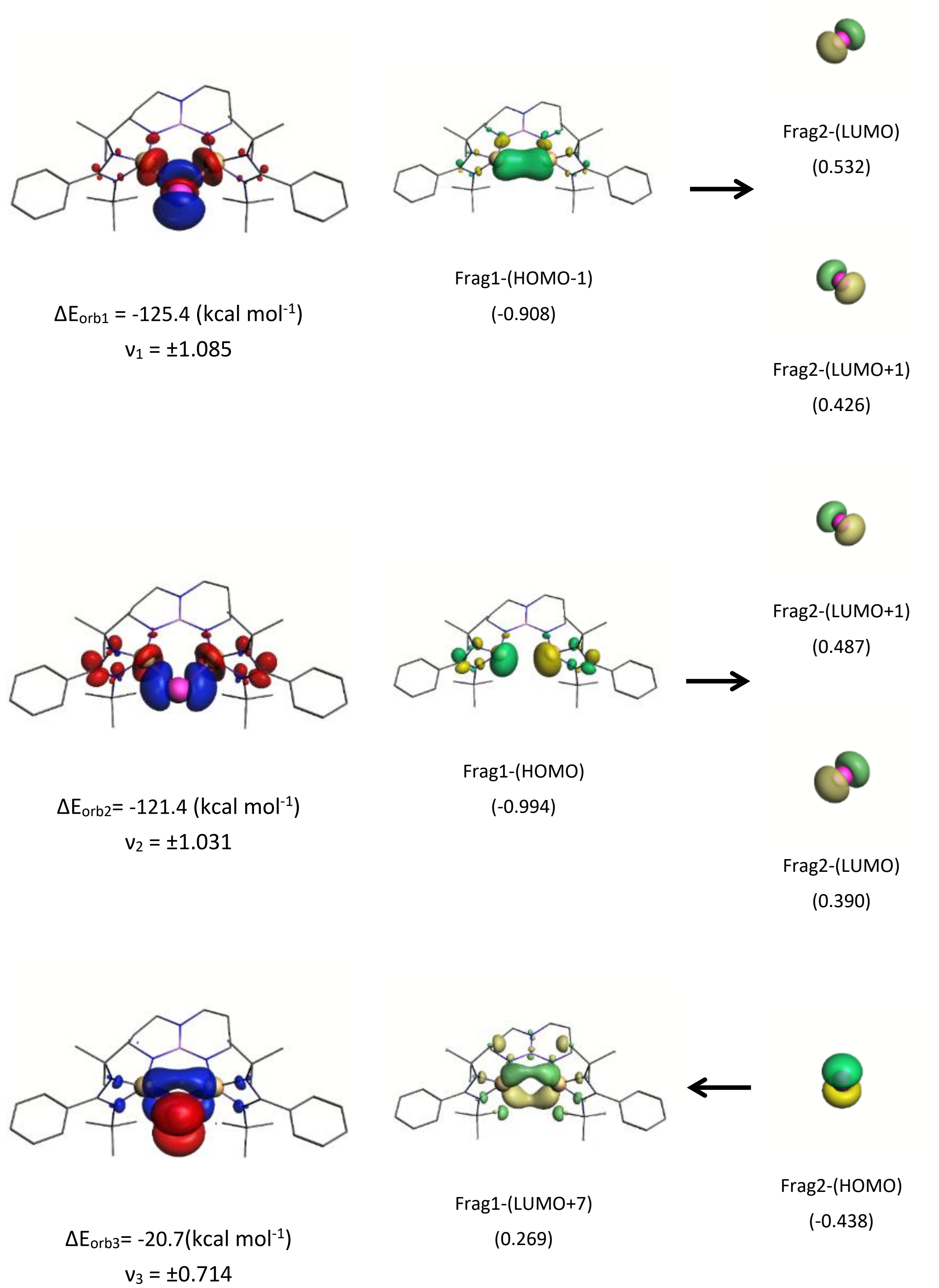

Supplementary Fig. 72. Plot of deformation densities Δρ of the pairwise orbital interactions and the associated interaction energies (ΔE_{orb}) between fragments, as well as the shape of the most important interacting MOs of the two fragments [(TBDSi$_2$)Pn][BArF$_4$] and Sb^+ in **2**. The direction of the charge flow is red to blue.

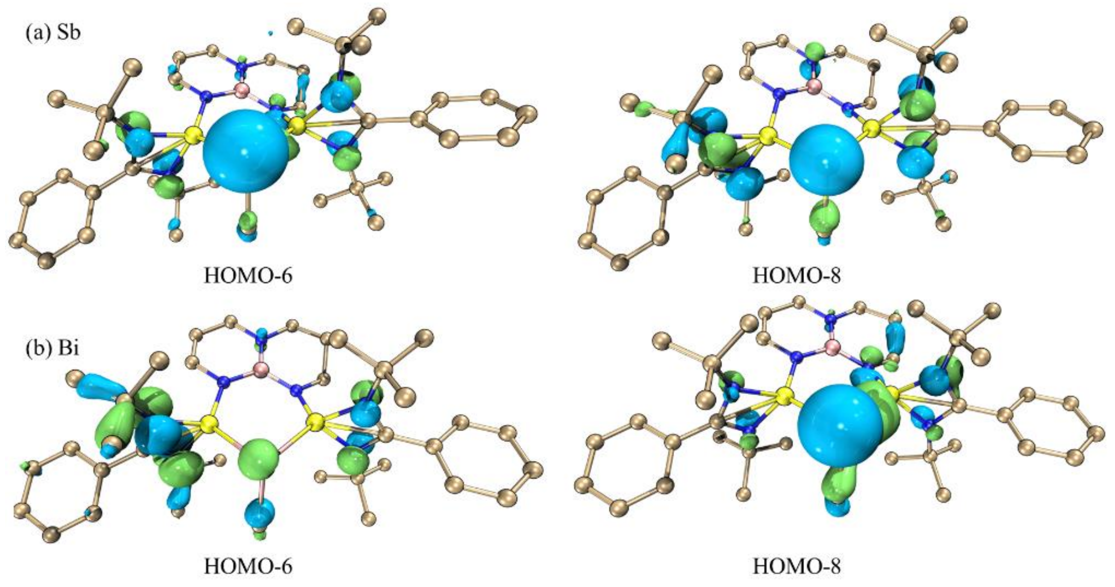

Supplementary Fig. 73. Selected Kohn-Sham isosurfaces (0.05 au) showing HOMO-6 and HOMO–8 in **4** and **5**.

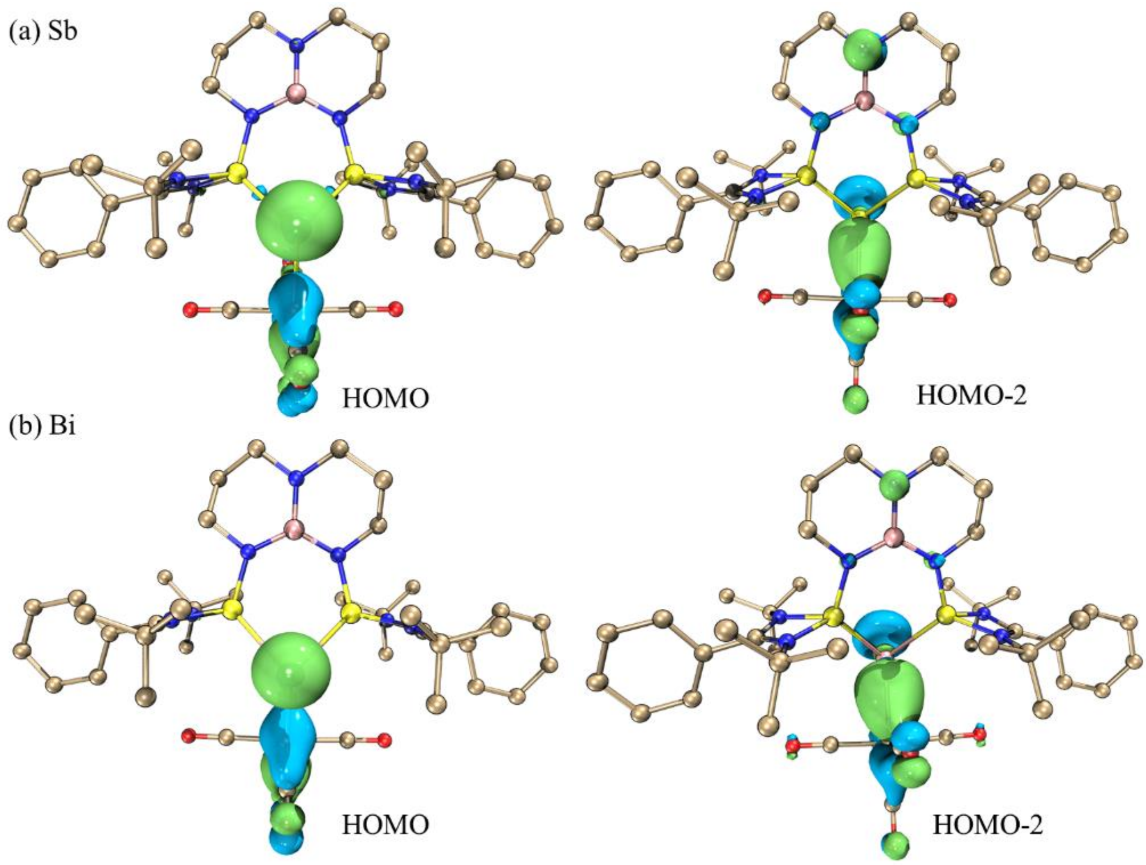

Supplementary Fig. 74. Selected Kohn-Sham isosurfaces (0.05 au) showing HOMO and HOMO–2 in **6** and **8**.

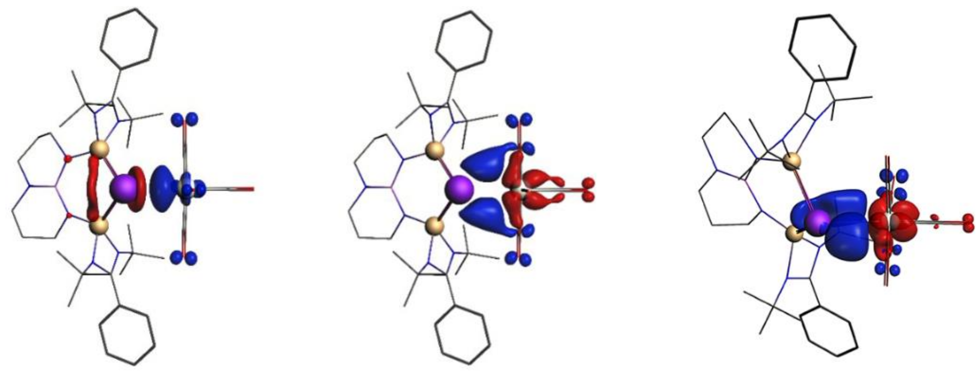

Δρ1 ΔE_{orb1} = -43.7 kcal mol^{-1} $\nu_1 = \pm 0.850$

Δρ2 ΔE_{orb2} = -3.5 kcal mol^{-1} $\nu_2 = \pm 0.239$

Δρ3 ΔE_{orb3} = -3.1 kcal mol^{-1} $\nu_3 = \pm 0.199$

Supplementary Fig. 75. Plot of deformation densities Δρ1-Δρ3 of **8** by using the singlet(S) Bi^{+}[bis(silylene)] and $Cr(CO)_5$ interacting fragments in their Singlet (S) states, with the associated interaction energies ΔE_{orb} (in kcal·mol^{-1}). The eigenvalues ν are a measure for the relative amount of charge transfer. The direction of the charge flow is from red → blue.

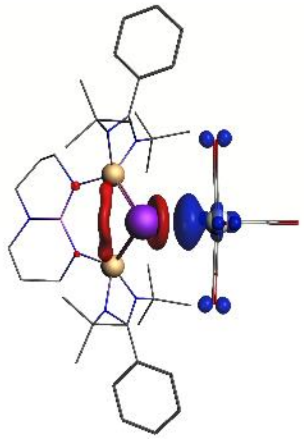

ΔE_{orb1} = -43.7(kcal mol^{-1})

$\nu_1 = \pm 0.850$

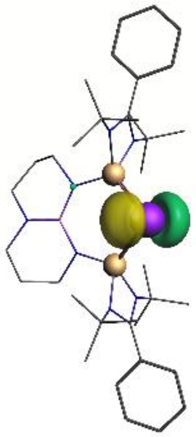

Frag1-(HOMO)

(-0.641)

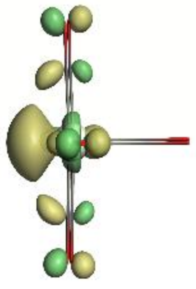

Frag2-(LUMO)

(0.663)

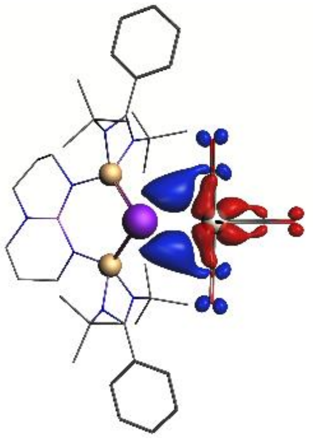

ΔE_{orb2} = -3.5 (kcal mol^{-1})

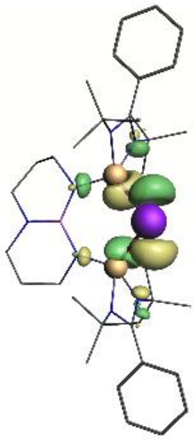

Frag1-(LUMO+6)

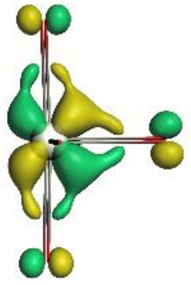

Frag2-(HOMO-1)

(-0.046)

$v_2 = \pm 0.239$	(0.033)

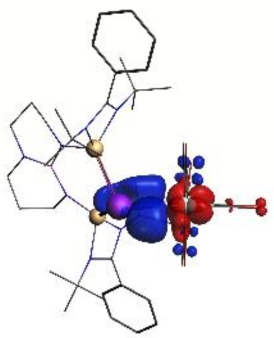

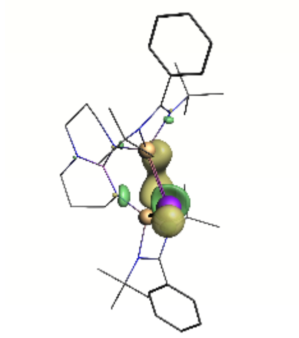

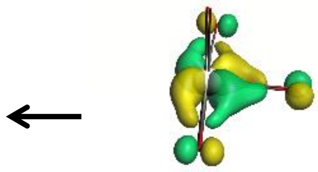

ΔE_{orb3} = -3.1(kcal mol^{-1})	Frag1-(LUMO+7)	Frag2-(HOMO)
$v_3 = \pm 0.199$	(0.012)	(-0.037)

Supplementary Fig. 76. Plot of deformation densities Δρ of the pairwise orbital interactions and the associated interaction energies (ΔE_{orb}) between fragments, as well as the shape of the most important interacting MOs of the two fragments Bi^{+}[bis(silylene)] and $Cr(CO)_5$ in **8**. The direction of the charge flow is red to blue.

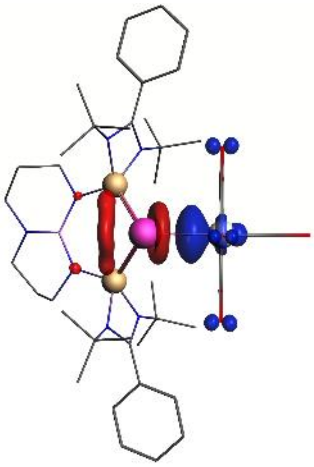

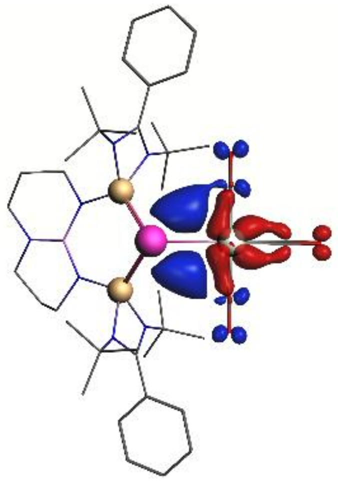

ΔE_{orb1} = -42.1 (kcal mol^{-1})	ΔE_{orb2} = -4.2 (kcal mol^{-1})
$v_1 = \pm 0.804$	$v_2 = \pm 0.251$

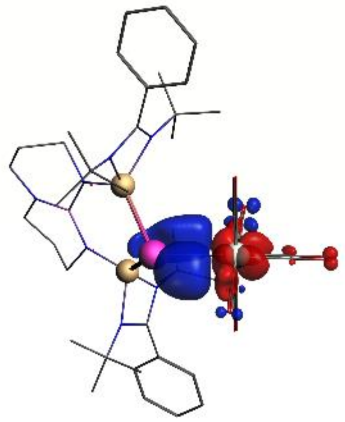

ΔE_{orb3} = -3.4 (kcal mol^{-1})

ν_3 = ±0.198

Supplementary Fig. 77. Plot of deformation densities Δρ of the pairwise orbital interactions between the two fragments of Sb^+[bis(silylene)] and $Cr(CO)_5$ in **6** together with the associated interaction energies ΔE_{orb} (in kcal mol^{-1}). The eigenvalues ν are a measure for the relative amount of charge transfer. The direction of the charge flow is from red to blue.

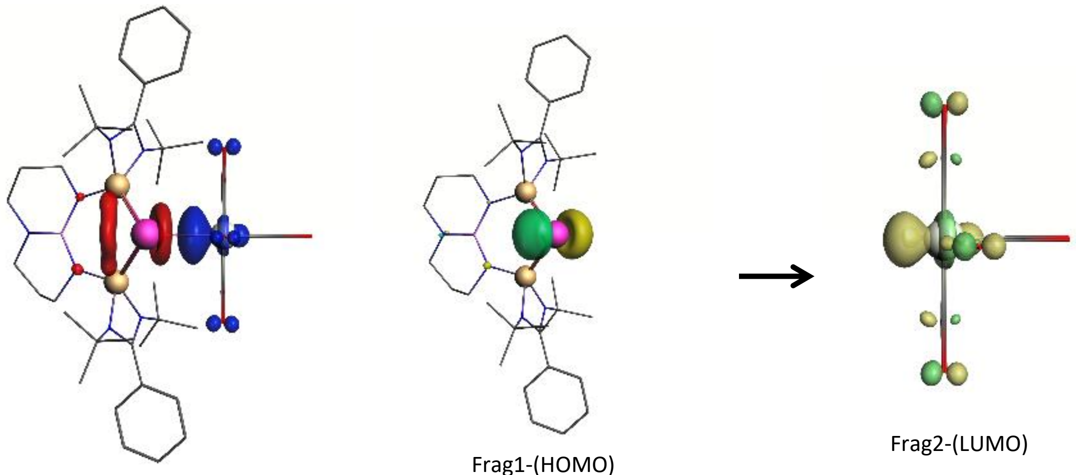

ΔE_{orb1} = -42.1 (kcal mol^{-1})

ν_1 = ±0.804

Frag1-(HOMO)

(-0.542)

Frag2-(LUMO)

(0.626)

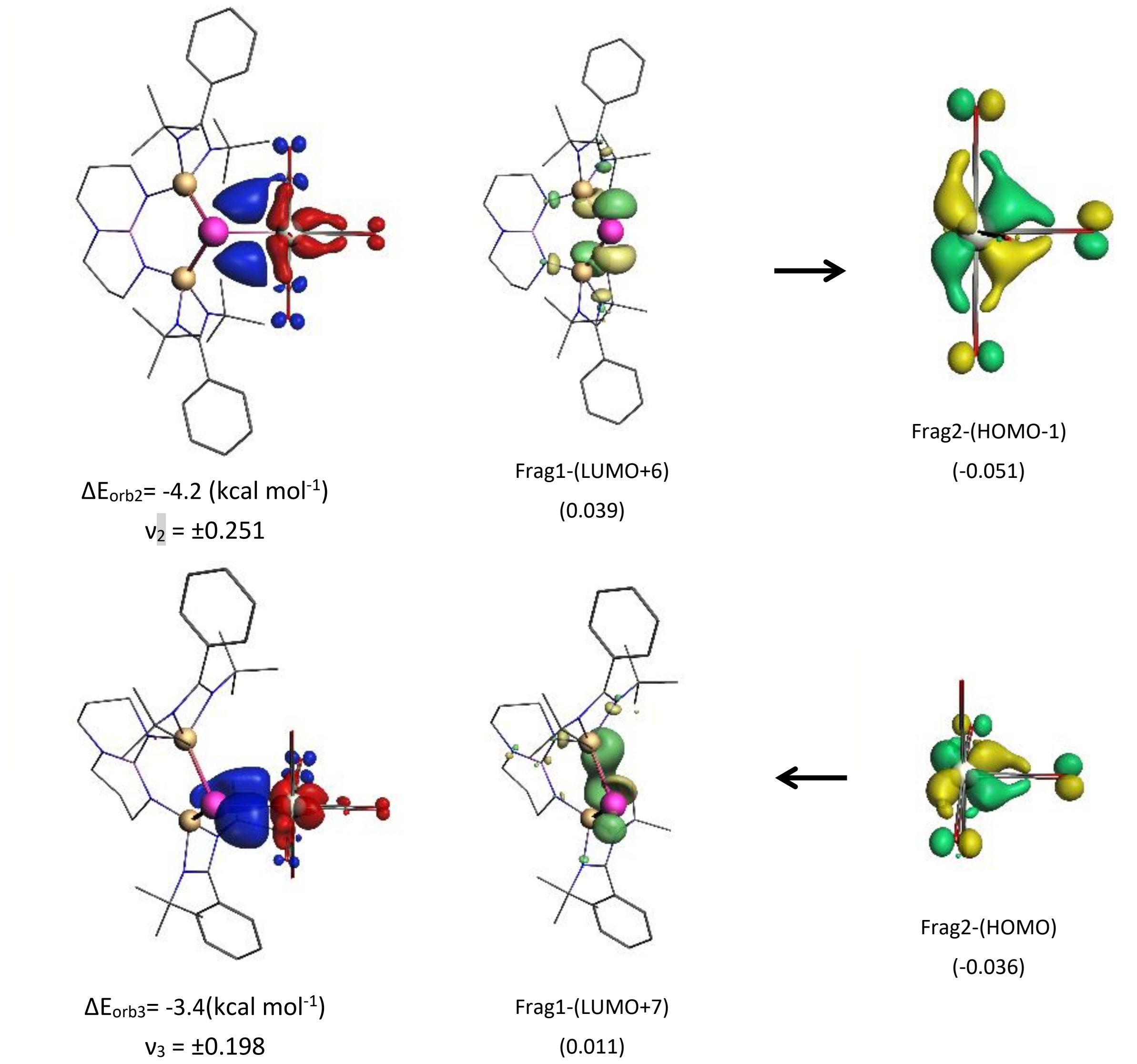

Supplementary Fig. 78. Plot of deformation densities Δρ of the pairwise orbital interactions and the associated interaction energies (ΔE_{orb}) between fragments, as well as the shape of the most important interacting MOs of the two fragments Sb^{+}[bis(silylene)] and $Cr(CO)_5$ in **6**. The direction of the charge flow is red to blue.

Supplementary Tab. 4. DFT calculations of fluoride ion affinity (FIA) and hydride ion affinity (HIA) analyses of compounds **2–5** at the BP86+D3(BJ)/def2-TZVPP level. Energy values are given in kcal/mol.

	FIA	HIA
**2**	114.2	113.0
**3**	115.3	113.7
**4**	192.8	201.4
**5**	190.2	196.4

Supplementary Tab. 5. ^{31}P NMR Chemical Shifts (parts per million) and Acceptor Numbers (ANs) of Compounds 2, 3, 4, and 5 Determined with the Gutmann−Beckett Method with 1 equiv of $OPEt_3$ as a Lewis Base.

entry	compound	δ $^{31}P^{1}\{H\}$ [ppm]	AN
**1**	2	50.8	22
**2**	3	51.3	23
**3**	4	54.0	29
**4**	5	55.2	31

Supplementary Tab. 6. Cartesian coordinates of the optimized geometries.

2

Sb	-0.000045	-1.567096	-0.000543
Si	-1.642122	0.272020	0.004090
Si	1.642103	0.271938	-0.004554
N	-1.284826	1.951646	0.029599
N	3.090096	-0.079463	-1.093565
N	3.112418	-0.069023	1.057850
N	1.284857	1.951590	-0.030329
N	-3.089823	-0.079229	1.093480
N	-3.112652	-0.069274	-1.057936
N	0.000039	4.078448	-0.000188
C	-2.490483	2.811890	0.149016
H	-2.634753	3.110306	1.202172
H	-3.378347	2.239073	-0.150871
C	3.842618	-0.401418	-0.025167
C	5.183231	-1.025078	-0.034911
C	-3.842600	-0.401413	0.025329
C	3.328157	-0.292359	2.504291
C	-5.183190	-1.025122	0.035631
C	-2.351945	4.048368	-0.726481
H	-2.182539	3.732695	-1.768156
H	-3.278616	4.639331	-0.703640
C	3.284850	-0.300261	-2.543632
C	2.490505	2.811836	-0.149873
H	2.634614	3.110294	-1.203035
H	3.378413	2.239008	0.149848
C	-3.284238	-0.299444	2.543679
C	6.331650	-0.220812	-0.052406
H	6.232547	0.864771	-0.061138
C	-1.188134	4.893560	-0.234733
H	-0.945134	5.676850	-0.974720
H	-1.470862	5.420808	0.697089
C	-6.331606	-0.220838	0.053170
H	-6.232484	0.864746	0.061410
C	5.303227	-2.421687	-0.023959
H	4.407434	-3.042878	-0.011522
C	3.197240	-1.802134	-2.862294
H	4.022924	-2.353322	-2.394449
H	3.256286	-1.960342	-3.947936
H	2.243534	-2.213403	-2.502546
C	1.188262	4.893514	0.234158
H	0.945429	5.676839	0.974168
H	1.470876	5.420738	-0.697723
C	2.352119	4.048272	0.725709
H	2.182847	3.732552	1.767387
H	3.278802	4.639217	0.702776
C	-3.328815	-0.293064	-2.504257
C	4.693382	0.251858	2.946395
H	4.770494	0.195291	4.040613
H	4.807944	1.303675	2.648935
H	5.521410	-0.326330	2.520163
B	0.000035	2.648634	-0.000303
C	7.594178	-0.814337	-0.058086
H	8.485715	-0.187224	-0.071516
C	-2.126834	0.442787	3.228404
H	-1.159825	0.046795	2.884390
H	-2.186638	0.308674	4.315944
H	-2.162849	1.518682	3.008523
C	-4.624834	0.284514	3.010199
H	-4.711756	1.340194	2.717473
H	-4.686402	0.226159	4.105313
H	-5.476493	-0.267071	2.595231
C	-3.197474	-1.801261	2.862824
H	-4.023904	-2.352062	2.395850
H	-3.255752	-1.959011	3.948576
H	-2.244335	-2.213321	2.502491
C	3.202929	-1.790505	2.828383

H	4.002968	-2.365827	2.345145
H	2.230896	-2.174761	2.488290
H	3.279126	-1.948965	3.912916
C	2.205747	0.485842	3.206784
H	2.276628	0.349722	4.293407
H	1.221087	0.122002	2.877008
H	2.272554	1.559505	2.983776
C	-5.303195	-2.421727	0.025233
H	-4.407411	-3.042931	0.012741
C	2.128099	0.442483	-3.228927
H	1.160749	0.047458	-2.884769
H	2.187876	0.307667	-4.316385
H	2.164974	1.518479	-3.009678
C	-2.206359	0.484600	-3.207303
H	-1.221737	0.120419	-2.877834
H	-2.272694	1.558325	-2.984413
H	-2.277717	0.348384	-4.293886
C	4.625933	0.282670	-3.010015
H	4.713658	1.338273	-2.717249
H	4.687498	0.224325	-4.105131

H	5.477155	-0.269578	-2.595043
C	-7.594136	-0.814352	0.059481
H	-8.485667	-0.187230	0.072924
C	6.570073	-3.008914	-0.029871
H	6.661247	-4.095164	-0.021515
C	7.714860	-2.207483	-0.046609
H	8.702616	-2.668630	-0.051004
C	-3.204089	-1.791323	-2.827896
H	-3.280487	-1.950104	-3.912370
H	-4.004199	-2.366302	-2.344375
H	-2.232101	-2.175756	-2.487853
C	-4.694007	0.251417	-2.946138
H	-4.808080	1.303413	-2.649143
H	-5.522074	-0.326284	-2.519319
H	-4.771536	0.194338	-4.040299
C	-7.714823	-2.207504	0.048591
H	-8.702577	-2.668648	0.053527
C	-6.570047	-3.008942	0.031774
H	-6.661228	-4.095195	0.023836

3

Bi	0.000132	-1.550673	-0.000007
Si	-1.662166	0.387139	0.002326
Si	1.662190	0.387376	-0.002277
N	-3.141585	0.069336	-1.058686
N	3.141527	0.069290	1.058735
N	-0.000144	4.189812	0.000117
N	-1.287290	2.064744	0.030636
N	3.116735	0.055299	-1.092368
N	1.287261	2.064931	-0.030516
N	-3.116699	0.055032	1.092416
C	-3.873787	-0.258118	0.024739
C	3.873774	-0.258017	-0.024709
C	-3.360830	-0.152592	-2.504723
C	1.184885	5.007087	0.242813
H	0.937611	5.784932	0.987245
H	1.467361	5.541359	-0.685113
C	-5.217958	-0.874651	0.035405
C	-3.314623	-0.159484	2.542831
C	3.360814	-0.152689	2.504757
C	2.350541	4.162539	0.729816
H	2.183297	3.840704	1.769937
H	3.276176	4.755223	0.708764

C	5.217882	-0.874688	-0.035472
C	2.488558	2.931601	-0.153051
H	2.624204	3.236412	-1.205520
H	3.380946	2.361523	0.137636
C	-5.345514	-2.270660	0.030596
H	-4.452979	-2.896632	0.021335
C	3.314667	-0.158915	-2.542823
C	-2.488617	2.931353	0.153449
H	-2.624073	3.236110	1.205959
H	-3.381031	2.361256	-0.137095
C	-1.185228	5.007002	-0.242554
H	-0.938067	5.784818	-0.987059
H	-1.467662	5.541317	0.685359
C	-2.225958	0.605735	-3.208983
H	-2.274124	1.680429	-2.986242
H	-2.300670	0.470411	-4.295471
H	-1.247175	0.225200	-2.880238
C	-6.362459	-0.064767	0.048215
H	-6.257840	1.020336	0.052222
C	-2.350862	4.162333	-0.729401
H	-2.183753	3.840550	-1.769556
H	-3.276567	4.754906	-0.708186

C	2.149166	0.570858	-3.227285
H	2.172557	1.646688	-3.005635
H	1.186446	0.163170	-2.884576
H	2.211436	0.439107	-4.314999
B	-0.000070	2.758932	0.000060
C	-3.246586	-1.661204	2.867220
H	-4.077456	-2.204207	2.399103
H	-3.310302	-1.814699	3.953280
H	-2.296376	-2.085254	2.512406
C	-3.261412	-1.652756	-2.828576
H	-2.294281	-2.052507	-2.491547
H	-3.342552	-1.810433	-3.912863
H	-4.069275	-2.215110	-2.343187
C	6.362464	-0.064914	-0.048259
H	6.257952	1.020200	-0.052060
C	-4.716947	0.414548	-2.946363
H	-5.554284	-0.148484	-2.517966
H	-4.796649	0.357788	-4.040396
H	-4.812484	1.468591	-2.650119
C	-2.149247	0.570404	3.227394
H	-1.186455	0.163068	2.884488
H	-2.211387	0.438338	4.315076
H	-2.172951	1.646291	3.006040
C	-6.615213	-2.851588	0.037954
H	-6.711928	-3.937402	0.034123
C	3.261737	-1.652881	2.828511
H	4.069845	-2.215017	2.343287
H	2.294784	-2.052867	2.491233
H	3.342660	-1.810601	3.912808
C	4.716798	0.414779	2.946400
H	4.796641	0.357769	4.040408
H	4.811950	1.468923	2.650400
H	5.554262	-0.147886	2.517767
C	5.345303	-2.270711	-0.030885
H	4.452704	-2.896594	-0.021619
C	-7.628082	-0.651780	0.055750
H	-8.516399	-0.020035	0.065806
C	-4.648779	0.441788	3.006052
H	-4.722447	1.497564	2.710092
H	-4.712612	0.387384	4.101248
H	-5.506447	-0.100665	2.591342
C	4.648712	0.442707	-3.005906
H	4.712731	0.388210	-4.101084
H	5.506469	-0.099466	-2.591009
H	4.722039	1.498532	-2.710029
C	2.225749	0.605333	3.209045
H	1.247068	0.224454	2.880427
H	2.273559	1.680025	2.986190
H	2.300614	0.470163	4.295543
C	-7.755957	-2.044288	0.050387
H	-8.746057	-2.500378	0.056130
C	7.628032	-0.652051	-0.056017
H	8.516410	-0.020390	-0.066024
C	3.246956	-1.660590	-2.867488
H	2.296895	-2.084918	-2.512627
H	4.078008	-2.203491	-2.399575
H	3.310572	-1.813863	-3.953585
C	6.614943	-2.851759	-0.038476
H	6.711553	-3.937582	-0.034803
C	7.755766	-2.044570	-0.050916
H	8.745819	-2.500761	-0.056903

6

Sb	-0.016111	-0.729031	-1.056507
Cr	0.001371	-3.132227	0.139466
Si	-1.719338	0.868706	-0.124941
Si	1.711857	0.860229	-0.159954
N	0.005292	4.637749	0.135329
N	-3.036976	0.402682	1.061337
N	-1.283551	2.518568	0.068915
N	1.308148	2.529329	-0.061564
N	-3.300252	0.704072	-1.058424
O	0.045057	-2.037403	2.972758
N	3.042718	0.430408	1.023474
N	3.274529	0.636787	-1.111269
C	-1.178123	5.475562	-0.048026
H	-0.949743	6.262995	-0.788052
H	-1.408443	5.995790	0.901425
O	-0.000805	-5.912891	1.295061
O	3.040592	-3.136560	0.071729
C	1.187535	5.444871	0.425709
H	0.931064	6.181355	1.207570
H	1.475120	6.026918	-0.470821

C	-2.378467	4.658300	-0.492217
H	-2.287574	4.374572	-1.552285
H	-3.296590	5.252587	-0.382790
C	-2.453781	3.395052	0.350231
H	-2.473630	3.655535	1.422635
H	-3.380617	2.852346	0.127027
C	2.348168	4.576585	0.874995
H	2.165843	4.184797	1.888430
H	3.271587	5.171874	0.909448
C	-3.644499	0.496669	-2.488703
C	-3.186873	0.300485	2.533997
C	-3.941305	0.316778	0.066424
C	2.506285	3.414388	-0.090641
H	2.660543	3.792455	-1.115645
H	3.398110	2.831789	0.172041
C	-6.318223	0.990708	0.229841
H	-5.991932	2.029162	0.167804
C	-5.368908	-0.043452	0.191958
C	-2.630617	1.331650	-3.284179
H	-2.727725	2.398523	-3.039811
H	-2.805562	1.204943	-4.360074
H	-1.599200	1.015507	-3.069432
C	3.933274	0.302912	0.018846
C	-1.855508	0.796477	3.109908
H	-1.022049	0.206609	2.708755
H	-1.849561	0.681618	4.200721
H	-1.687229	1.855250	2.869651
O	-3.037722	-3.158349	0.015386
C	5.365007	-0.043023	0.141647
C	5.788762	-1.372906	0.259207
H	5.056669	-2.177240	0.258385
C	-7.672577	0.685428	0.352495
H	-8.406998	1.490203	0.387870
C	3.213729	0.406677	2.496370
C	3.604258	0.384567	-2.537814
C	-4.331731	1.192996	3.035780
H	-4.205645	2.227386	2.687072
H	-4.326996	1.200459	4.133984
H	-5.310469	0.826433	2.706298
C	2.595080	1.208439	-3.350984
H	2.762248	1.047989	-4.423622
H	2.707007	2.281077	-3.140278
H	1.561589	0.910806	-3.121477
C	3.443051	-1.115099	-2.835797
H	2.418243	-1.451301	-2.622926
H	4.135740	-1.711642	-2.228135
H	3.651342	-1.316378	-3.895230
B	0.012118	3.210422	0.036885
C	0.017471	-2.421009	1.875138
O	0.035535	-4.289213	-2.685394
C	-8.085807	-0.649082	0.426224
H	-9.145977	-0.886328	0.516854
C	-3.426185	-1.162466	2.933740
H	-4.382498	-1.527465	2.539770
H	-3.451666	-1.248935	4.028284
H	-2.623158	-1.806048	2.557828
C	-5.063253	0.986975	-2.808683
H	-5.833553	0.356043	-2.352339
H	-5.208525	0.961637	-3.897010
H	-5.207854	2.021885	-2.469630
C	7.153305	-1.653885	0.363892
H	7.482170	-2.689858	0.447158
C	-3.504233	-0.995483	-2.831838
H	-2.481670	-1.349697	-2.637886
H	-3.724330	-1.163396	-3.894687
H	-4.199046	-1.601298	-2.235819
C	-5.779724	-1.380159	0.272542
H	-5.040892	-2.177681	0.237384
C	1.878670	-3.108767	0.102365
C	1.867280	0.867719	3.066151
H	1.885139	0.822950	4.162000
H	1.060514	0.210429	2.718879
H	1.639612	1.898574	2.761467
C	5.026389	0.846644	-2.883854
H	5.161055	0.785917	-3.972112
H	5.792889	0.220048	-2.415335
H	5.187367	1.889450	-2.578262
C	6.305456	0.999905	0.136988
H	5.969415	2.032876	0.043197
C	4.319181	1.378945	2.934957
H	4.128015	2.389563	2.547083
H	5.308719	1.051809	2.595964
H	4.339363	1.433822	4.031678
C	8.089883	-0.617403	0.363867
H	9.153100	-0.842613	0.449367
C	7.663900	0.710464	0.253508
H	8.391376	1.522300	0.254597
C	-1.877368	-3.121338	0.076334

C	-7.140507	-1.676683	0.383303
H	-7.459445	-2.717674	0.437478
C	0.000482	-4.845206	0.844358
C	0.019495	-3.841848	-1.618445
C	3.525728	-1.021582	2.965394
H	3.565645	-1.051306	4.062537
H	4.494206	-1.362138	2.579456
H	2.748971	-1.719096	2.631520

8

Bi	0.015657	-0.747313	-1.099860
Cr	-0.002936	-3.138595	0.282441
Si	-1.733725	0.924642	-0.111925
Si	1.739671	0.932779	-0.079655
O	-3.040207	-3.080973	0.149569
N	-3.312224	0.725890	-1.050064
N	-1.313346	2.591646	-0.009323
N	3.337772	0.798720	-0.996000
O	-0.031552	-4.457439	-2.468966
O	-0.007849	-5.858697	1.570106
O	-0.045235	-1.949310	3.078228
N	-3.061726	0.509618	1.080650
N	3.052050	0.476082	1.117352
O	3.034464	-3.114243	0.108473
N	1.284320	2.577925	0.125753
C	-5.794538	-1.315288	0.316441
H	-5.052800	-2.110709	0.297310
C	3.966229	0.397168	0.131806
C	-3.960332	0.383238	0.083991
C	-1.878260	-3.082015	0.213102
C	-5.387461	0.021306	0.216376
N	-0.004506	4.696335	0.189420
C	-0.006225	-4.813393	1.068947
C	5.388574	0.019655	0.266516
C	-6.339814	1.053013	0.238734
H	-6.016762	2.091297	0.158838
C	6.351929	1.039977	0.319316
H	6.040715	2.083274	0.261215
C	3.187500	0.365470	2.590887
C	5.779862	-1.323341	0.342377
H	5.029827	-2.110091	0.297316
C	-7.154543	-1.614050	0.430466
H	-7.470395	-2.655020	0.500864
C	-0.017876	-3.945527	-1.430170
C	-2.508152	3.482603	-0.028045
H	-2.665000	3.866894	-1.050361
H	-3.401048	2.902085	0.235009
C	1.874269	-3.101054	0.195766
C	3.418478	-1.099429	2.987771
H	2.616582	-1.739027	2.603219
H	3.434074	-1.189165	4.082224
H	4.376757	-1.466674	2.600848
C	1.177291	5.535553	0.000747
H	1.401220	6.066487	0.945758
H	0.949451	6.314560	-0.748404
C	-0.019035	-2.362381	1.990846
C	1.852976	0.863883	3.156937
H	1.692631	1.925412	2.924113
H	1.834279	0.738863	4.246455
H	1.020937	0.281684	2.741996
C	-3.215834	0.467965	2.554595
C	-1.866870	0.934693	3.113355
H	-1.866668	0.867892	4.208173
H	-1.656005	1.973655	2.825017
H	-1.057696	0.294058	2.741077
C	-3.661869	0.485505	-2.472949
B	-0.013651	3.268144	0.089685
C	7.135262	-1.639865	0.462962
H	7.438970	-2.685582	0.513627
C	2.679082	1.415163	-3.227901
H	2.866310	1.292865	-4.302203
H	2.753112	2.483273	-2.981384
H	1.650555	1.081646	-3.024325
C	2.447129	3.459640	0.423755
H	2.445791	3.726294	1.494798
H	3.379536	2.918802	0.221380
C	-7.693650	0.745650	0.363909
H	-8.430470	1.548724	0.385785
C	-1.182988	5.505922	0.488376
H	-1.474628	6.090621	-0.405145
H	-0.919428	6.240200	1.269997
C	3.699904	0.598762	-2.422345
C	7.700772	0.714747	0.452083
H	8.446138	1.508776	0.499404

C	2.382681	4.717578	-0.427186
H	2.303829	4.427217	-1.486402
H	3.299220	5.313088	-0.311190
C	-8.103033	-0.588750	0.456668
H	-9.162627	-0.827798	0.549331
C	-3.537013	-1.015445	-2.781998
H	-3.763195	-1.206594	-3.839629
H	-4.233351	-1.601385	-2.168400
H	-2.517638	-1.376847	-2.584257
C	-2.342474	4.639399	0.942195
H	-2.154730	4.242869	1.952784
H	-3.264466	5.236401	0.984770
C	4.330840	1.252507	3.105750
H	5.311155	0.883853	2.783274
H	4.316739	1.255855	4.203884
H	4.210893	2.288572	2.759797
C	8.094469	-0.625916	0.520805
H	9.150334	-0.878669	0.619494
C	-2.647119	1.293767	-3.294613
H	-1.615613	0.979389	-3.077461
H	-2.737184	2.367396	-3.079117
H	-2.827237	1.139858	-4.366074
C	3.595960	-0.894942	-2.771785
H	2.579562	-1.273385	-2.590826
H	4.295669	-1.488474	-2.169337
H	3.831694	-1.055535	-3.832399
C	-3.511623	-0.967207	3.012527
H	-2.732301	-1.654786	2.664930
H	-4.480553	-1.312247	2.631692
H	-3.541215	-1.007917	4.109621
C	5.111675	1.119519	-2.726685
H	5.269492	1.102246	-3.813445
H	5.889527	0.502557	-2.264267
H	5.230566	2.155537	-2.381170
C	-4.324004	1.427147	3.014581
H	-4.330843	1.473275	4.111865
H	-5.314780	1.093753	2.685405
H	-4.146385	2.442286	2.632029
C	-5.078353	0.979085	-2.800390
H	-5.226930	0.930966	-3.887497
H	-5.214160	2.022036	-2.483340
H	-5.851705	0.363965	-2.328103

3. Supplementary References

[1] Dolomanov O. V., Bourhis L. J., Gildea R. J., Howard J. A. K., Puschmann H., OLEX2: A Complete Structure Solution, Refinement and Analysis Program. J. Appl. Cryst. **42**, 339-341 (2009).

[2] Sheldrick G.M., SHELXS-90/96, Program for Structure Solution, Acta Crystallogr. Sect. A ,**46**, 467 (1990).

[3] Frisch, MJ. Trucks GW., Schlegel HB., Scuseria GE, Robb MA, Cheeseman JR., Scalmani G, Barone V, Petersson GA, Nakatsuji H, Caricato X. Li, M., Marenich AV., Bloino J, Janesko BG, Gomperts R., Mennucci B, Hratchian HP, Ortiz JV, Izmaylov AF, Sonnenberg JL, Williams-Young D, Ding F, Lipparini F, Egidi F, Goings J, Peng B, Petrone A, Henderson T, Ranasinghe D, Ranasinghe, Zakrzewski VG., Gao J, Rega N, Zheng G, Liang W, Hada M, Ehara M, Toyota K, Fukuda R, Hasegawa J, Ishida M, Nakajima T, Honda Y, Kitao O, Nakai H, Vreven T, Throssell K, Montgomery JA, Jr, Peralta JE, Ogliaro F, Bearpark MJ, JJ, Brothers EN, Kudin KN, Staroverov VN, Keith TA, Kobayashi R, Normand J, Raghavachari K, Rendell AP, Burant JC, Iyengar SS, Tomasi J, Cossi M, Millam JM, Klene M, Adamo C, Cammi R, Ochterski JW, Martin RL, Morokuma K, Farkas O, Foresman JB, and Fox DJ, Gaussian, Inc., Wallingford CT, (2016).

[4] Glendening ED, Landis CR., Weinhold F, NBO 7.0: New vistas in localized and delocalized chemical bonding theory. *J. Comput. Chem.* **40**, 2234–2241 (2019).

[5] Lu T, Multiwfn 3.5 - A Multifunctional Wavefunction Analyzer, School of Chemical and Biological Engineering, University of Science and Technology, Beijing (2018).

[6] Lu T, Chen F. Multiwfn: A multifunctional wavefunction analyzer. *J Comput Chem* **33**, 580-592 (2012).

[7] Mitoraj MP, Michalak A, Ziegler T. A Combined Charge and Energy Decomposition Scheme for Bond Analysis. *J Chem Theory Comput* **5**, 962-975 (2009).

[8] Humphrey W, Dalke A, Schulten K. VMD: Visual molecular dynamics. *J Mol Graph* **14**, 33-38 (1996).

[9] Legault CY. CYLview, 1.0b, Université de Sherbrooke, Sherbrooke (Québec) Canada, http://www.cylview.org (2009).

[10] Ziegler T, Rauk A. On the calculation of bonding energies by the Hartree Fock slater method. *Theor Chim Acta* **46**, 1-10 (1977).

[11] Zhao L, Hopffgarten MV, Andrada DM, Frenking G. Energy decomposition analysis. *WIREs Comput Mol Sci* **8**, e1345(2018).

[12] Mitoraj M, Michalak A. Donor–acceptor properties of ligands from the natural orbitals for chemical valence. *Organometallics* **26**, 6576-6580 (2007).

[13] Mitoraj M, Michalak A. Applications of natural orbitals for chemical valence in a description of bonding in conjugated molecules. *J Mol Model* **14**, 681-687 (2008).

[14] Amsterdam S. Density Functional 2019 (ADF2019), Theoretical Chemistry, Vrije Universiteit, Amsterdam, Netherlands, http://www.scm.com.

[15] Velde GT, Bickelhaupt FM, Baerends EJ, Guerra CF, van Gisbergen SJA, Snijders J G, Ziegler T. Chemistry with ADF. *J Comput Chem* **22**, 931-967 (2001).

[16] Lenthe EV, Baerends EJ. Optimized slater-type basis sets for the elements 1–118. *J Comput Chem* **24**, 1142-1156 (2003).

[17] van Lenthe E, Baerends EJ, Snijders JG. Relativistic regular two-component Hamiltonians. *J Chem Phys* **99**, 4597 (1993)

[18] Bickelhaupt FM, Nibbering NMM, van Wezenbeek EM, Baerends EJ. Central bond in the three CN.cntdot.dimers NC-CN, CN-CN and CN-NC: Electron pair bonding and Pauli repulsion effects. *J Phys Chem* **96**, 4864-4873(1992).

[19] Krapp A, Bickelhaupt FM, Frenking G. Orbital overlap and chemical bonding. *Chem Eur J* **12**, 9196-9216 (2006).

[20] Bickelhaupt FM, Frenking G. The chemical bond - fundamental aspects of chemical bonding, Frenking G, Shaik SS. Eds. (Wiley-VCH, Weinheim), pp. 121-157 (2014).

[21] Zhao L, Hermann M, Schwarz WHE, Frenking G. The Lewis electron-pair bonding model: Modern energy decomposition analysis. *Nat Rev Chem* **3**, 48-63 (2019).

[22] Zhao L, Hermann M, Holzmann N, Frenking G. Dative bonding in main group compounds. *Coord Chem Rev* **344**, 163-204 (2017).
